# A Stable-Isotope Mass Spectrometry-Based Metabolic Footprinting Approach to Analyze Exudates from Phytoplankton

**DOI:** 10.3390/md11114158

**Published:** 2013-10-29

**Authors:** Ralf J. M. Weber, Erik Selander, Ulf Sommer, Mark R. Viant

**Affiliations:** 1 School of Biosciences, College of Life and Environmental Sciences, University of Birmingham, Edgbaston, Birmingham, B15 2TT, UK; E-Mail: r.j.weber@bham.ac.uk; 2 Centre for Ocean Life, National Institute of Aquatic Resources, Technical University of Denmark, Kavalergården 6, 2920 Charlottenlund, Denmark; E-Mail: erik.selander@bioenv.gu.se; 3 NERC Biomolecular Analysis Facility-Metabolomics Node (NBAF-B), University of Birmingham, Edgbaston, Birmingham, B15 2TT, UK; E-Mail: u.sommer@bham.ac.uk

**Keywords:** metabolomics, stable isotope, algal exudate, dinoflagellate, correlation analysis, DIMS, exometabolome, FT-ICR, ^13^C, chemical ecology

## Abstract

Phytoplankton exudates play an important role in pelagic ecology and biogeochemical cycles of elements. Exuded compounds fuel the microbial food web and often encompass bioactive secondary metabolites like sex pheromones, allelochemicals, antibiotics, or feeding attractants that mediate biological interactions. Despite this importance, little is known about the bioactive compounds present in phytoplankton exudates. We report a stable-isotope metabolic footprinting method to characterise exudates from aquatic autotrophs. Exudates from ^13^C-enriched alga were concentrated by solid phase extraction and analysed by high-resolution Fourier transform ion cyclotron resonance mass spectrometry. We used the harmful algal bloom forming dinoflagellate *Alexandrium tamarense* to prove the method. An algorithm was developed to automatically pinpoint just those metabolites with highly ^13^C-enriched isotope signatures, allowing us to discover algal exudates from the complex seawater background. The stable-isotope pattern (SIP) of the detected metabolites then allowed for more accurate assignment to an empirical formula, a critical first step in their identification. This automated workflow provides an effective way to explore the chemical nature of the solutes exuded from phytoplankton cells and will facilitate the discovery of novel dissolved bioactive compounds.

## 1. Introduction

Phytoplankton exude large amounts of organic compounds to the surrounding water [1]. The exuded compounds, or exometabolome, contain primary metabolites such as lipids [2], carbohydrates, amino acids and secondary metabolites. Exudates mediate a variety of biological interactions [3], for example, sexual pheromones that assist mate finding [4], allelopathic compounds that deter or intoxicate grazers and suppress competing species [5], and metabolites that guide microbes and grazers to algal cells and patches of elevated resources [6,7]. Exudates are also an important carbon source for heterotrophic bacteria [8]. All of these small scale molecular processes and biological interactions between species ultimately drive important, larger scale ecological mechanisms. In addition, phytoplankton contribute *ca.* 50% of the global carbon fixation [9]. Despite these facts there is surprisingly little known about the chemical nature of bioactive compounds produced and released by phytoplankton.

At low cell densities, phytoplankton exudates form a solute cloud around the cell, limited by the release rate and molecular diffusion of these exudates, with high concentration only occurring close to the cell surface. If cell density increases the background concentrations of exudates build up and may have far reaching effects [10]. Unknown toxic exudates from a *Chrysochromulina poylepis* bloom in the Kattegatt and Skagerrak Sea, for example, resulted in mass mortality of fish and benthic invertebrates [11,12]. Allelochemicals from *Alexandrium tamarense* structure pelagic communities [5]. Chemical compounds from algal blooms or biofilms also guide other organisms, such as nematodes, fish and seabirds, to productive hotspots in the sea [13,14]. These types of chemically mediated effects may often be as, or more important than, direct trophic interactions in structuring the pelagic community [15]. Identifying and characterizing the phytoplankton “footprint” from the complex background of dissolved organic matter in seawater is, however, a considerable analytical challenge and many bioactive compounds of phytoplankton origin still remain unknown.

Mass spectrometry (MS)-based metabolomics has advanced rapidly in the past decade and is increasingly routinely used for the identification and quantification of small molecules within complex mixtures, such as cells, tissues and whole organisms [16,17]. A variety of conceptual targeted and untargeted MS approaches have been developed, in particular metabolic fingerprinting, an untargeted method that combines analytical and computational analyses to reveal intracellular metabolism [18]. Conversely, metabolic footprinting is an approach to simultaneously study metabolites consumed from, and/or released into, the cell’s local environment [19]. Metabolic footprinting is a relatively rapid and information-rich method for studying multiple released compounds under different environmental conditions and therefore offers a potential short-cut in relation to traditional bioassay guided fractionation, in which samples are partitioned and biotested sequentially in the search for active compounds. Nonetheless, characterising low concentration algal exudates in seawater against the highly complex dissolved organic background is extremely challenging, requiring an analytical technique with high resolution, high mass accuracy and the ability to span a wide dynamic range. Fourier transform ion cyclotron resonance (FT-ICR) MS has the highest mass accuracy and mass resolution of all commercially available mass spectrometers and has proven to be powerful platform across a large variety of metabolomics studies [20,21,22]. Nevertheless, there remain considerable challenges in annotating the *m/z* (mass-to-charge) measurements in mass spectrometry-based metabolomics experiments [17].

Stable isotope labelling is a well-established method in biochemistry and is routinely used to study biochemical pathways in plants [23,24], cells [25,26] and organisms [27,28]. More recently, the method has been used to assist metabolic profiling in pelagic cyanobacteria [29,30]. Stable isotopes are readily distinguishable from their more abundant natural isotopes by high resolution MS, even within a complex mixture. This approach provides an opportunity to focus selectively on biologically synthesised metabolites. By increasing the proportion of ^13^C in the inorganic carbon sources utilized solely by photoautotrophic organisms, metabolites produced by these autotrophs will obtain a unique isotope signature, hereafter referred to as a stable isotope pattern (SIP). Using high resolution MS-based approaches the ^13^C-labelled released organic compounds of algal origin can be discriminated from the highly complex dissolved organic compounds and other contaminants. Using a combination of unlabelled, ^13^C labelled, and ^15^N labelled alga, Baran and colleagues were able to eliminate approximately 90% of the features detected in *Synechococcus* cell extracts and growth media (*i.e*., compounds of non-biogenic origin). They were only able to detect a limited number of exudates in the cell media due to their low concentrations but concluded that the method is well suited for metabolic profiling of pelagic autotrophs [29].

**Figure 1:**
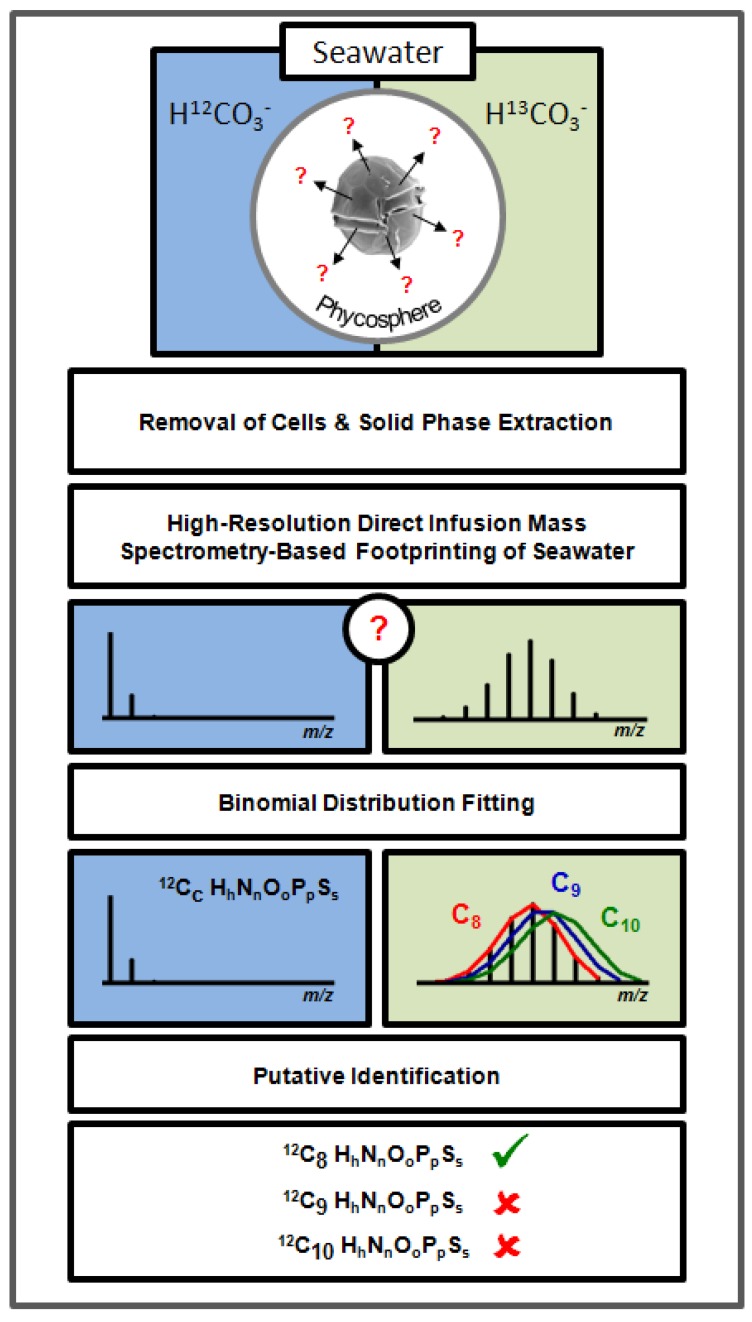
Flow scheme for the stable isotope metabolic footprinting approach for marine microalgae. Algal cells are cultured in either ^12^C (control with natural isotopic distribution) or ^13^C-enriched media. Cells are removed by filtration and metabolites in the cell free filtrate concentrated onto solid phase extraction columns. Eluted compounds are then analyzed using FT-ICR mass spectrometry and a novel algorithm is used to automatically locate the stable isotope patterns, compare them to theoretical isotope intensity profiles, and output the empirical formula(e) of the exuded metabolite(s).

Here we report the development of a methodological framework to selectively analyze and annotate exudates from aquatic autotrophs using both high resolution direct infusion (DI) FT-ICR MS and stable isotope labelling experiments (Figure 1). *Alexandrium tamarense* cultures were ^13^C-enriched by addition of ^13^C-bicarbonate to the seawater. Then the compounds exuded from these algae, as well as controls with natural isotope composition, were measured using DI FT-ICR MS. A novel computational workflow was developed to subsequently annotate, in an automated manner, only those released compounds that were selectively ^13^C-enriched in the mass spectral footprints. Building on the method described by Baran *et al*. (2010) [29], we describe a method that can also handle scenarios where near 100% labelling is not achievable, and methods to sample and desalt larger quantities of exudates.

## 2. Results and Discussion

### 2.1. High-Resolution Mass Spectrometry-Based Metabolic Footprinting

**Figure 2:**
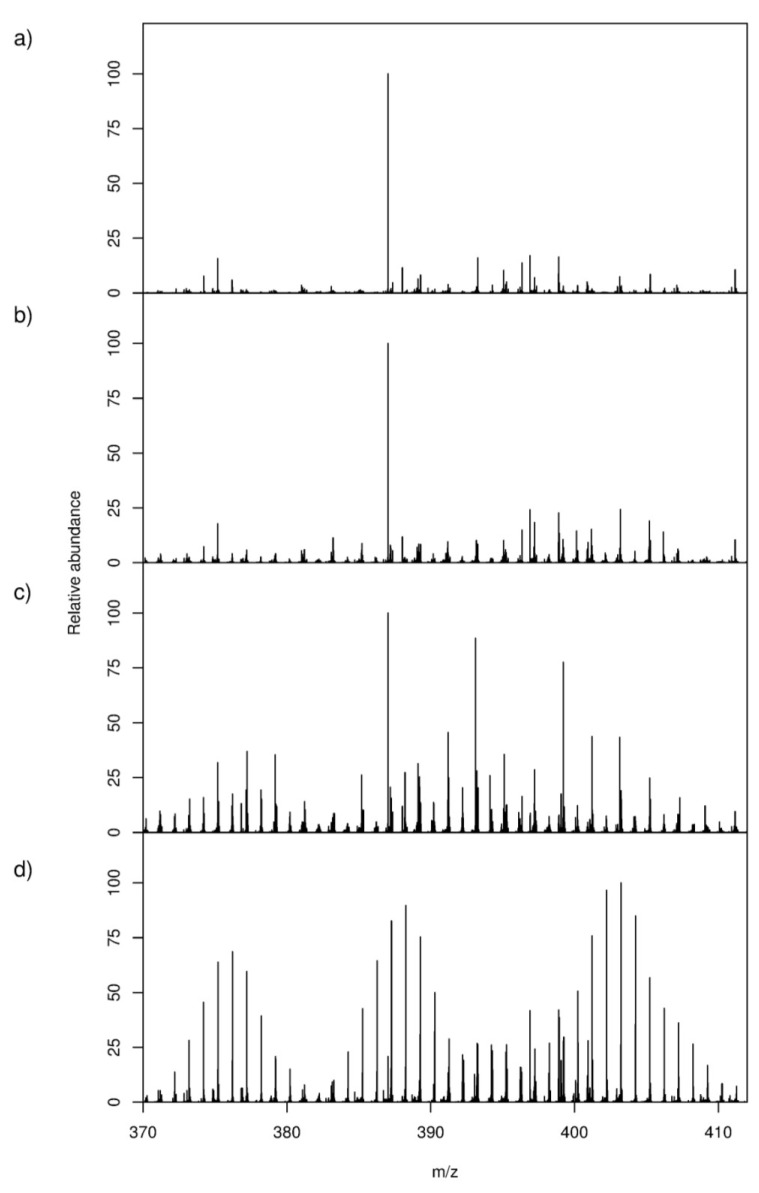
Representative region of mass spectra (*m/z* 370–412) from negative ion direct infusion (DI) FT-ICR metabolic footprinting analyses across four different sample groups: (**a**,**b**) unlabelled and ^13^C-labelled seawater without algal cells, respectively, as controls, and (**c**,**d**) unlabelled and ^13^C-labelled cultures of *Alexandrium tamarense*, respectively; all according to the workflow described in Figure 1. The bottom panel shows several prominent isotope patterns arising from ^13^C-labelled exudates that are absent from all other spectra, indicating successful incorporation of the stable isotope into *A. tamarense*’s biochemical pathways and subsequent transfer to the culture media.

**Figure 3:**
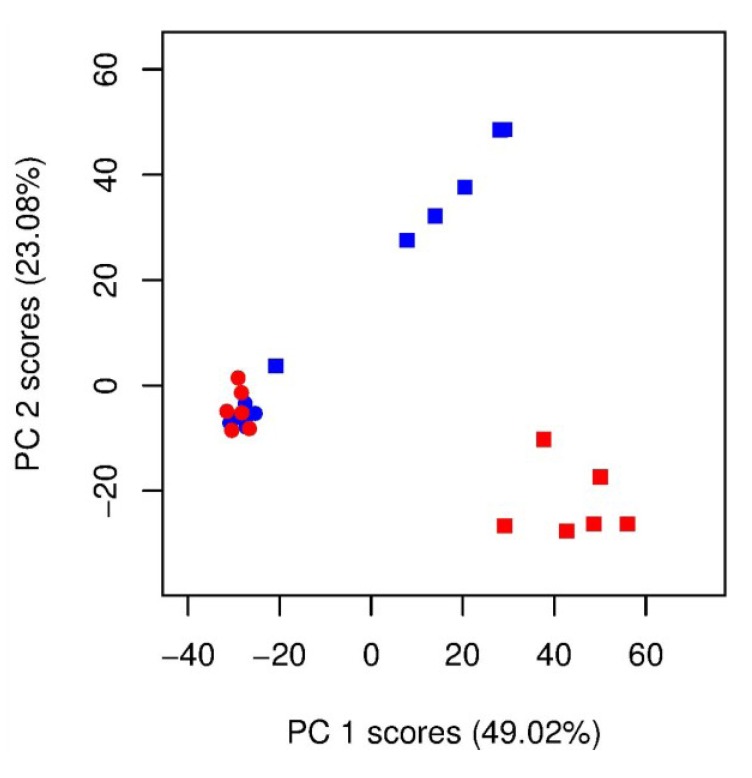
Principal component analysis scores plot from analysis of the DI FT-ICR mass spectra (*m/z* 70–590) from a metabolic footprinting study of (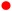) unlabelled and ^13^C-labelled (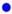) seawater without algal cells (as controls, *n* = 6 each), and (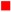) unlabelled and (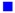) ^13^C-labelled cultures of *Alexandrium tamarense* (*n* = 6 each). The major separation along the PC1 axis corresponds to the differences between the metabolic footprints of seawater samples with *versus* without algal cells present. Separation along PC2 corresponds to differences between the metabolic footprints of ^13^C-labelled *vs.* unlabelled *A. tamarense* cultures*.*

Four groups of samples (*i.e*., derived from unlabelled and ^13^C-labelled seawater without cells, and from unlabelled and ^13^C-labelled cultures of *Alexandrium tamarense*) were studied so as to provide a robust experimental design with appropriate controls. *A. tamarense* and the seawater-only controls were cultured in parallel under identical conditions and differed only in the carbon source that was added to the system, either unlabelled or ^13^C-labelled bicarbonate. An optimized DI FT-ICR MS selective ion monitoring (SIM) stitching method was used to maximize the detection and putative annotation of the *A. tamarense* exometabolome. This approach combines multiple, narrow mass spectra into a single wide-scan spectrum to maximize sensitivity and mass accuracy. The mass spectra of the unlabelled and ^13^C-labelled seawater controls were highly similar (Figure 2a,b), which indicates the expected lack of incorporation of ^13^C into dissolved organic matter in the seawater. However, several prominent SIPs (*i.e*., multiple stable-isotope patterns) were observed in the mass spectra of ^13^C-labelled cultures that were absent in the unlabelled cultures (Figure 2c,d), confirming successful incorporation of the stable isotope into *A. tamarense*’s biochemical pathways and subsequent transfer to the culture media. Collectively these findings demonstrate that we can visually discriminate biochemically synthesised exudates from the milieu of chemicals in seawater. Cultures were not axenic, and it is possible that algal metabolites were modified by associated bacteria. Mass spectra of the four sample groups were further investigated using principal component analysis (PCA) to visualize any differences in the overall metabolic footprints (Figure 3). The seawater control samples cluster together regardless of stable isotope labelling and are separated along the PC1 axis of the scores plot from both the unlabelled and ^13^C-labelled cultures. Samples from ^13^C-labelled and unlabelled cultures are clearly separated along the PC2 axis (Figure 3), with this metabolic difference caused by the presence of SIPs that were only present in ^13^C-labelled cultures (Figure 2d). A single outlier can be observed in the ^13^C-labelled algal samples, closer to the control samples, which we deduced was caused by a lower concentration of exudates in that particular sample, potentially due to a problem during sample preparation. Quality control (QC) samples are relatively well grouped which demonstrates the consistency of the mass spectrometric analyses (Supplementary Figure S1). These results confirm the successful uptake of ^13^C bicarbonate into the algal metabolism that shifts the overall metabolic footprints, or exudates, of those samples.

### 2.2. Locating Stable Isotope Patterns

**Figure 4:**
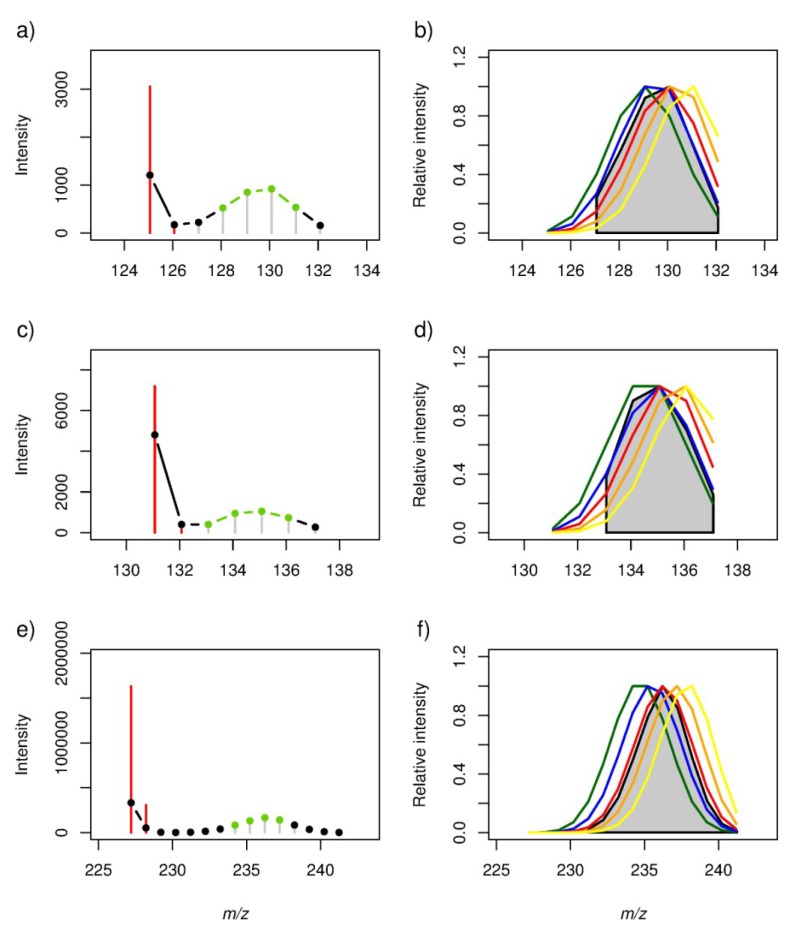
(**a**,**c**,**e**) A representative subset of the stable isotope patterns used for estimating the labelling efficiency observed in DI FT-ICR mass spectra collected from ^13^C-labelled cultures of *Alexandrium tamarense*. The “signal intensity template” (see Methods) is presented in green, additional ^13^C-labelled peaks in black, and isotopic peaks observed in mass spectra of unlabelled *A. tamarense* samples in red. Based on accurate mass measurements and the number of peaks, each SIP was annotated with a single formula: [C_7_H_10_O_2_ − H]^−^, [C_6_H_12_O_3_ − H]^−^ and [C_14_H_28_O_2_ − H]^−^ for the three plots, respectively; (**b**,**d**,**f**) Simulated stable isotope patterns (SIPs) modelled for each empirical formula applying five different labelling efficiencies: 50% (green), 55% (blue), 60% (red), 65% (orange) and 70% (yellow). SIPs are shown as continuous data to avoid the presentation of overlapping peak intensities; the experimental data for ^13^C-labelled *A. tamarense*, identical to that in the plots on the left, are shown in grey, the average labelling efficiency was estimated to be ~58% (see Table 1, including the Pearson’s correlation coefficients (*r*) and associated *p*-values for each comparison between an experimental and simulated SIP).

SIPs were initially located in the ^13^C-labelled *A. tamarense* spectra by searching for groups of four peaks with specific signal intensity patterns (*i.e*., the first peak in the 4-peak pattern should have a lower intensity than the second peak, and the third peak in the pattern should have a higher intensity than the fourth peak) and ^12^C–^13^C peak differences, hereafter referred to as a “template”, which represents the highest intensity peaks of a stable isotope pattern (Figure 4, shown in green). Overall *ca.* 300 templates were observed in the FT-ICR MS dataset. To locate the complete SIP, additional ^13^C-labelled peaks were added on both sides of the template as described in the Methods. Finally, each SIP was matched to its corresponding all-^12^C peak (or if available a naturally occurring ^12^C–^13^C isotope peak pair) in the unlabelled *A. tamarense* mass spectra (Figure 4). This final step defines the original unlabelled peak in each SIP, which subsequently is used for putative annotation of the exuded metabolite (see Section 2.4). Despite the high sensitivity and mass accuracy of FT-ICR MS, several non-ideal stable-isotope patterns were found (Supplementary Figure S2); these arose because (1) the signal intensity of partially or fully-labelled compound dropped below the detection level of the FT-ICR MS, which resulted in missing peaks across the SIP; (2) a single *m/z* peak resulted from two or more metabolites of similar mass, which resulted in an altered isotope intensity profile; (3) some *m/z* features were falsely assigned to a particular SIP in part because of the finite mass accuracy of the FT-ICR; or (4) no all-^12^C peak or ^12^C–^13^C isotope peak pair could be located. Nonetheless, over 100 SIPs of the *ca.* 200 SIPs that were selected for putative annotation did not suffer from these problems.

**Table 1:** Pearson’s correlation coefficient (*r*) and associated *p*-value for each comparison between an experimental and simulated stable isotope pattern; specifically to determine which simulated SIP (across five different colour coded labelling efficiencies) is most similar to each experimental SIP in Figure 4, this analysis is used to estimate the overall ^13^C-isotope labelling efficiency in the experiment.

m/z (all-^12^C containing peak as [M − H]^−^ ion)	Empirical formula	Labelling Efficiency (%)									
		*r*-value					*p*-value				
		50%	55%	60%	65%	70%	50%	55%	60%	65%	70%
125.06084	^12^C_7_H_10_O_2_	0.86	0.99	0.93	0.68	0.35	0.0289	0.0001	0.0080	0.1347	0.4913
131.07140	^12^C_6_H_12_O_3_	0.92	0.99	0.82	0.48	0.12	0.0244	0.0010	0.0918	0.4181	0.8532
227.20143	^12^C_14_H_28_O_2_	0.63	0.90	1.00	0.88	0.58	0.0369	0.0002	0.0000	0.0004	0.0615

### 2.3. Estimation of the Stable Isotope Labelling Efficiency

As we have shown, SIPs allow the discrimination between *m/z* signals from released metabolites and the background milieu of chemicals in seawater. Furthermore, SIPs represent a discrete probability distribution of the number of ^12^C atoms that have been replaced with ^13^C atoms as a result of the stable isotope labelling. Therefore this distribution includes information regarding the total number of C atoms in a particular metabolite, which can help to annotate unknown metabolites (see Section 2.4). However, such calculations are dependent upon knowing the ^13^C-isotope labelling efficiency in the experiment (Equation 2). This labelling efficiency has a direct effect on the observed isotope distribution, becoming right-shifted as the percentage of labelling efficiency increases (Supplementary Figure S3). Consequently we can accurately estimate the efficiency of ^13^C-labelling from the isotope distributions in the experimental data, but this should only be calculated from isotope distributions for which we know the empirical formula unambiguously (termed “well-behaved” SIPs, below). Therefore, first several SIPs were investigated manually, and based on the number of *m/z* measurements and the profile of the isotope distribution a crude labelling efficiency of approximately 60% was estimated (Supplementary Figure S3, spanning a possible labelling efficiency from 1.1% to 100%). Subsequently, based on this estimate, a more accurate labelling efficiency was calculated by modelling a series of binomial distributions (Equation 2) and then comparing these to several well-behaved experimental SIPs using Pearson's correlation analysis (Figure 4 and Table 1). The criteria for the well-behaved SIPs included: only one empirical formula could be assigned to that SIP, and all possible ^12^C- and ^13^C-containing peaks were detected for that formula. For example, the three SIPs and corresponding empirical formulae listed in Table 1 were well-behaved, and were putatively annotated by examination of the KEGG database as [cyclohex-1-enecarboxylic acid or toluene-*cis*-dihydrodiol], [6-hydroxyhexanoic acid, d-2-hydroxyisocaproate, ethyl (*R*)-3-hydroxybutanoate, or paraldehyde] and [myristic acid] (a common saturated fatty acid), respectively. For still greater confidence in this annotation, the highest intensity SIP in Figure 4 was further investigated using solid phase extraction and tandem MS, revealing an MS/MS mass spectra consistent with a myristic acid standard (Supplementary Figure S4). Using just these well-behaved SIPs, the average labelling efficiency (*i.e*., that corresponds to the highest and most significant Pearson’s correlation) was estimated to be 58%.

### 2.4. Putative Annotation of Exuded Metabolites

**Figure 5:**
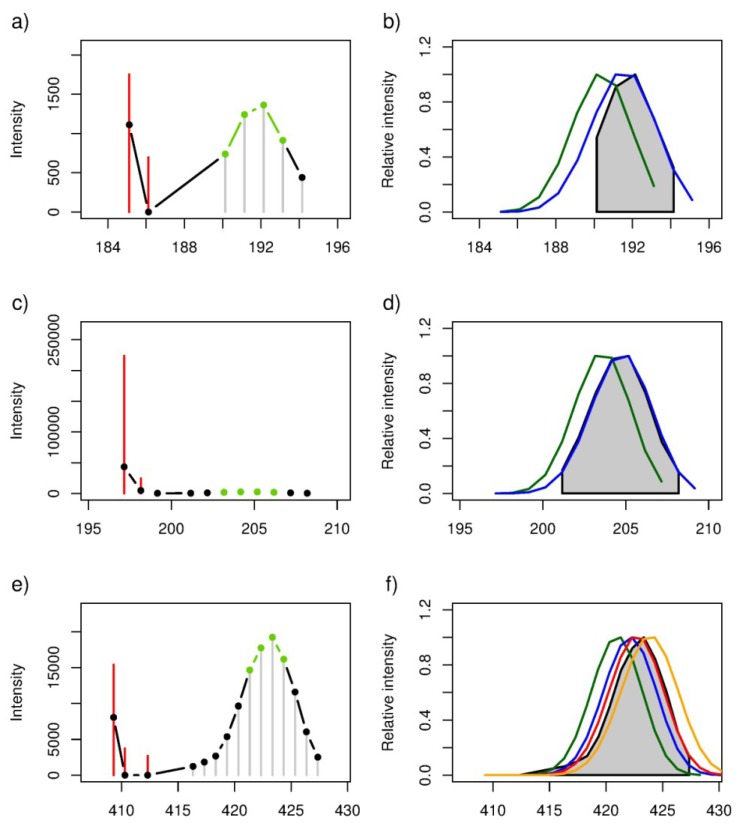
(**a**,**c**,**e**). Stable isotope patterns observed in DI FT-ICR mass spectra collected from ^13^C-labelled cultures of *Alexandrium tamarense*. Signal intensity template presented in green, additional ^13^C-labelled peaks in black, and isotopic peaks observed in mass spectra of unlabelled *A. tamarense* samples in red. (**b**,**d**,**f**). Simulated stable isotope patterns (SIPs) were modelled for each empirical formula assignment to the all-^12^C peak with a labelling efficiency of 58%, where the coloured profiles correspond to differing numbers of carbon atoms in the formula (defined in Table 2). The experimental data for ^13^C-labelled *A. tamarense*, identical to that in the plots on the left, are shown in grey. The results of the metabolite annotation are shown in Table 2.

Having discovered *ca.* 300 SIPs that arise from biologically synthesised metabolites and that have subsequently been transferred to the surrounding media, and having determined the average ^13^C-labelling efficiency in the experiment, we then putatively annotated this compound list. One of the greatest challenges in metabolomics is the annotation of peaks in mass spectra, in part because many of the mass features in a complex biological mass spectrum can be assigned to multiple empirical formulae. Using a mass spectrometer with high mass accuracy is important for this annotation step to reduce the number of possible assignments. The stable isotope labelling approach described here can further improve the confidence in metabolite annotation by eliminating incorrect putative empirical formula(e) assignments (in terms of the number of carbon atoms in the formula). The annotation workflow was applied to all SIPs that matched to an unlabelled ^12^C–^13^C isotope peak pair, specifically to *ca*. 200 SIPs. In this section, however, we focus only on a subset of these as shown in Figure 5 to highlight the metabolite annotation workflow in more detail. First, all possible empirical formulae were calculated for each of the three all-^12^C containing peaks in the SIPs (Table 2; mass accuracy of 1.5 ppm). As can be seen, high mass accuracy alone is insufficient to determine a single empirical formula for any of these metabolites, and for the highest molecular weight metabolite there are four possible formulae. The experimental SIPs were used to estimate the number of C atoms per metabolite by comparing them to simulated isotope distributions for a range of C atoms in the elemental composition of interest (Figure 5 and Table 2). The putative empirical formulae assignments were then ranked based on the degree of correlation between each simulated and experimental SIP; e.g., the all-^12^C containing peaks in the two SIPs shown in Figure 5a,b were both putatively annotated with two empirical formulae. However, based on the calculated correlations each of the two SIPs was clearly reduced to a single empirical formula, specifically C_10_H_18_O_3_ and C_12_H_22_O_2_, respectively. Both empirical formulae were assigned multiple putative metabolite names. The third experimental SIP was highly correlated with the theoretical SIPs of three empirical formulae, while a fourth empirical formula was discounted due to a poor correlation. None of the three empirical formulae was annotated as a KEGG compound. However, the highest correlated empirical formulae (*i.e*., C_22_H_38_O_3_, not present KEGG database) may represent a furan fatty acid which is known to exhibit radical-scavenging ability and anti-inflammatory properties in several organisms, including algae [31].

From the *ca.* 200 SIPs that were located, only the SIPs that were matched at least one simulated SIP with an *r*-value ≥0.7 were considered reliable, which was a total of 126 (Supplementary Table S1 and Figure S5). Many common primary metabolites from marine algae were observed, such as docosohexanoic acid and other polyunsaturated fatty acids. In addition we detected putative structures corresponding to more exotic compounds such as isophorone, known to attract sea lice parasites to salmonid fish [32], grayanotoxin, originally isolated from rhododendrons, and hormones such as hydroxycorticosterone and 11-β,21-dihydroxy-5-β-pregnane-3,20-dione. There is a bias towards compounds retained on the reversed phase SPE columns used here, and the extraction could be made more complete by using additional resins.

**Table 2:** Stable isotope patterns located and annotated in FT-ICR mass spectra collected from ^13^C-labeled cultures of *Alexandrium tamarense*.

*m/z*	Colour (in Figure 5)	Empirical Formula (peak)	Ion Form	Theoretical Mass (Da)	Mass Error (ppm)	KEGG Compound	*r*-value	*p*-value
185.11855	blue	C_10_H_18_O_3_	[M − H]^−^	185.11832	1.25	[(3*R*)-6-Hydroxy-3-isopropenyl-heptanoate, (3*S*)-6-Hydroxy-3-isopropenyl-heptanoate, (5*R*)-6-Hydroxy-5-isopropenyl-2-methylhexanoate, (5*S*)-6-Hydroxy-5-isopropenyl-2-methylhexanoate, 10-Oxodecanoate, 2-Oxodecanoic acid, Epomediol]	0.95	0.0115
	green	C_8_H_14_O	[M + Acetate]^−^	185.11832	1.25	[Sulcatone]	−0.09	0.9149
197.15491	blue	C_12_H_22_O_2_	[M − H]^−^	197.15470	1.05	[(−)-Menthyl acetate, Citronellyl acetate, Decanoyl acetaldehyde, Neomenthyl acetate]	1.00	0.0000
	green	C_10_H_18_	[M + Acetate]^−^	197.15470	1.05		0.58	0.1735
409.29594	red	C_22_H_38_O_3_	[M + Acetate]^−^	409.29595	−0.02		0.99	0.0000
	blue	C_21_H_45_N_2_O_2_P	[M + Acetate]^−^	409.29653	−1.45		0.91	0.0000
	orange	C_24_H_42_O_5_	[M − H]^−^	409.29595	−0.02		0.91	0.0000
	green	C_19_H_39_N_8_P	[M − H]^−^	409.29625	−0.77		0.55	0.0506

## 3. Experimental Section

### 3.1. Cell Culture and Stable Isotope Labelling

The dinoflagellate *Alexandrium tamarense* strain 975 was obtained from the Scandinavian Culture Collection of Algae and Protozoa [33]. *Alexandrium* spp. are known producers of paralytic shellfish toxin, a group of neurotoxic alkaloids [34], and in addition, produce allelopathic compounds that lyse competing species and ciliates causing changes in the pelagic community structure at relatively low cell concentrations [5]. *Alexandrium* cells are *ca.* 30 µm in diameter. Culture media was prepared from filtered seawater (0.22 µm) that had been pre-purified through two 300 mg Isolute ENV+ (Biotage) solid phase extraction (90 µm, 800 Å hydroxylated polystyrene-divinylbenzene co-polymer; SPE) columns to reduce background levels of dissolved organic compounds. The purified water was enriched with K-min medium [35], and spiked with either ^13^C bicarbonate or ^12^C bicarbonate (as a control) to a concentration of 4 mM. An *Alexandrium* culture was diluted with either ^13^C or ^12^C bicarbonate-containing media and divided between six 500 mL glass flasks for each treatment, approximately 400 mL in each. The percentage of ^13^C of total carbon in the alga (*C_t_*) will asymptotically approach that of the surrounding media as the cells divide according to the formula:

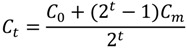
(1)
Where *t* refers to the number of divisions after incubation start, *C_0_* is the initial concentration of ^13^C in this case 1.1%, the natural abundance of ^13^C, and *C_m_* the ^13^C concentration in the enriched media. The labelling was continued for >6 cell divisions (verified by microscopic counts, data not shown) and ^13^C concentration in the algae should theoretically have reached within 2% of the surrounding media. In order to remove intermediately labelled compounds, 99% of the media was replaced by fresh media after this period by removing media from the inside of a submerged filter. Vitamins were omitted in this final media change to reduce the background of non algal organic compounds in the sample matrix. Cells were left to exude into the new media for two days at 16:8 h light:dark cycles, approximately 100 µmol s^−1^ m^−2^ (corresponding to near surface light levels) before metabolite extraction (*n* = 6 independent cultures for ^13^C-labelled and for unlabelled controls).

### 3.2. Metabolite Extraction from Media

At the end of the two-day incubation of the twenty-four culture beakers (six cell free controls of the ^12^C and ^13^C media, six ^12^C *Alexandrium* cultures, and six ^13^C *Alexandrium* cultures), we removed the algal cells by gentle suction filtration onto 45 mm GF/F filters (Whatman). This procedure may lyse some cells or cause the release of compounds from cells stressed by the filtration, and therefore one should be aware that the sample analysed by the mass spectrometer may contain, in addition to algal exudates, some intracellular algal compounds. The cell free filtrate was then pumped through a 100 mg ENV+ SPE column after washing with 1 mL MeOH and equilibrating with 1 mL water, using a peristaltic pump attached to the outlet luer of the column, to trap the exudates. The ENV+ is a polymeric (polystyrene) resin functionalized with phenol groups. Functionalized polymers have previously been shown to be superior to, for example, C18 packings to extract DOC from seawater samples [36]. Columns were desalted with 1 mL MQ grade water before elution with 1 mL methanol. The eluate was evaporated in a centrifugal concentrator, and resolved in 80:20 methanol:water containing 20 mM ammonium acetate, vortexed and then centrifuged prior to DI FT-ICR MS analyses. A set of QC samples were prepared by pooling an aliquot of each of these samples. The cell free media controls were produced exactly as described above, providing both ^13^C-labelled and unlabelled seawater controls (each *n* = 6).

### 3.3. FT-ICR MS Analysis, Spectral Processing and Unsupervised Multivariate Analyses

Negative ion mode MS analyses were conducted using a hybrid 7-Tesla linear ion trap FT-ICR mass spectrometer (LTQ FT Ultra, Thermo Fisher Scientific, Germany) equipped with a Triversa chip-based nanoelectrospray ion source (Advion Biosciences) using conditions as described previously [37,38]. Each of the 24 samples was analysed in triplicate from *m/z* 70 to 590 using the selected ion monitoring (SIM)-stitching approach, which was developed previously to maximise the dynamic range and mass accuracy of metabolomics analyses [37,38]. Mass spectra were processed using a three-stage signal filtering method as described by Payne *et al*. [39]. The resulting intensity matrices, representing the different metabolic profile for each sample, were further processed by the imputation of missing values [40], probabilistic quotient normalization and generalized-log transformed [41,42]. Finally, principal components analyses were conducted using the Matlab PLS-Toolbox (Eigenvector Research) to assess the metabolic similarities and differences between samples.

### 3.4. Locating and Putative Identification of SIPs

A novel computational algorithm was developed to locate stable isotope patterns (SIPs) in the partially processed (untransformed and not normalised) mass spectral data. First, three additional peak lists were created by subtracting the exact masses of ^13^C (13.00335 *m/z*), ^13^C_2_ (26.00670 *m/z*) and ^13^C_3_ (39.01005 *m/z*) from the one original peak list. These four lists were concatenated and then specific groupings of 4 closely spaced peaks were extracted if they met the following two criteria: (1) all 4 peaks should have the same *m/z* value, within an error range of 1.5 ppm (this is used to find ^13^C isotopic spacings); (2) the first peak in the pattern (ordered from low to high *m/z*) should have a lower intensity than the second peak, and the third peak in the pattern should have a higher intensity than the fourth peak (this is used to select the centre of a stable isotope pattern for compounds with a minimum of 4 carbon atoms). Each grouping of 4 peaks (see Figure 5a,b), hereafter referred to as a “template”, was further extended by including all the observed *m/z* values on both sides of the template with *n* × ^13^C–^12^C *m/z* difference(s) away from the first or last peak of the pattern. Once all the peaks (arising from ^13^C isotopes) that surround the template are located, termed a SIP, the original measured *m/z* values and intensities across each of the four sample groups (^13^C-labelled and unlabelled exudates and corresponding seawater-only controls were determined). This allows the examination of overlapping isotopic peaks between ^13^C-labelled and unlabelled mass spectra. Using MI-Pack metabolite annotation software [43], the all-^12^C containing peaks (observed in the unlabelled mass spectra and directly related to the SIP observed in the labelled spectra) were assigned an empirical formula(e) based upon their accurate mass measurement (*i.e*., elements and ion forms were restricted as follows: ^12^C_0–34_H_0–72_N_0–15_O_0–19_P_0–7_S_0–8_ and [M − H]^−^, [M + ^35^Cl]^−^ and [M + Acetate]^−^). Only those empirical formulae within a mass tolerance of 1.5 ppm were recorded. Next, all empirical formulae were filtered using the heuristic rules reported previously [44]. In many cases, each empirical formula was putatively assigned a metabolite name using the “single-peak search” approach in MI-Pack as well as the KEGG (Kyoto Encyclopedia of Genes and Genomes) database [43,45]. Next, for each observed SIP, a set of theoretical SIPs were calculated using a binomial density function (Equation 2). In short, the probability of finding a ^13^C atom in a particular elemental composition follows a binomial distribution, where *k* is the number of ^13^C atoms, *n* is the total number of carbon atoms in the empirical formula of interest, and *p* is the probability that any single carbon atom is ^13^C (*i.e*., the labelling efficiency, ~58% in this study, derived in Results).


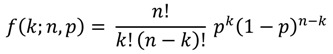
(2)

Pearson’s correlation coefficients (and corresponding *p*-values) were calculated between the intensities of the observed SIP and each of the corresponding theoretical SIPs, with the highest correlation revealing the most likely number of carbons in the empirical formula. The intensities of all observed and theoretical SIPs were normalised to a maximum intensity of one to facilitate their comparison. This estimated number of carbons in each empirical formula was then used to guide the annotation of the exuded compounds. Custom scripts were developed using Python and the R-language and are available on request from the corresponding author.

## 4. Conclusions

We have developed a methodological framework to explore the chemical nature of exudates from phytoplankton cells. This work represents a stable-isotope mass spectrometry-based metabolic footprinting experiment to map the exudates from *Alexandrium tamarense*. The labelling procedure applied here, using ^13^C-enriched bicarbonate, is straightforward, cost efficient, and functional for all micro algae species. The algorithm developed automatically separates the biologically synthesised metabolites from the complex seawater background, and then derives the carbon isotope signatures of these compounds which subsequently enables more accurate putative annotation. Liquid chromatography, tandem or multi-stage mass spectrometry (including the use of authentic standards for structural confirmation), are valuable additions for this methodological framework. Ultimately this approach, in combination with a suitable bioassay, should help to facilitate the discovery of novel bioactive compound candidates secreted from micro algae species.

## Supplementary Files

Supplementary File 1 Supplementary Materials (PDF, 8522 KB)
