# Supplementary material for: A Stable-Isotope Mass Spectrometry-Based Metabolic Footprinting Approach to Analyze Exudates from Phytoplankton
Source: Mar Drugs. 2013 Oct 29;11(11):4158–75. doi: 10.3390/md11114158 (PMC3853721; doi:10.3390/md11114158)

Supplementary Materials

Figure S1. PCA scores plot from analysis of mass spectra (70–590 *m/z*) from negative ion FT-ICR metabolic footprinting analyses of (●) unlabelled and ^{13}C-labelled (●) seawater without algal cells (as controls, *n* = 6 each), and (■) unlabelled and (■) ^{13}C-labelled cultures of *Alexandrium tamarense* (*n* = 6 each). Black triangles represent QC samples. The major separation along the PC1 axis corresponds to the differences between the metabolic footprints of seawater samples with *vs.* without algal cells present. Separation along PC2 corresponds to differences between the metabolic footprints of ^{13}C-labelled *vs.* unlabelled *A. tamarense* cultures.

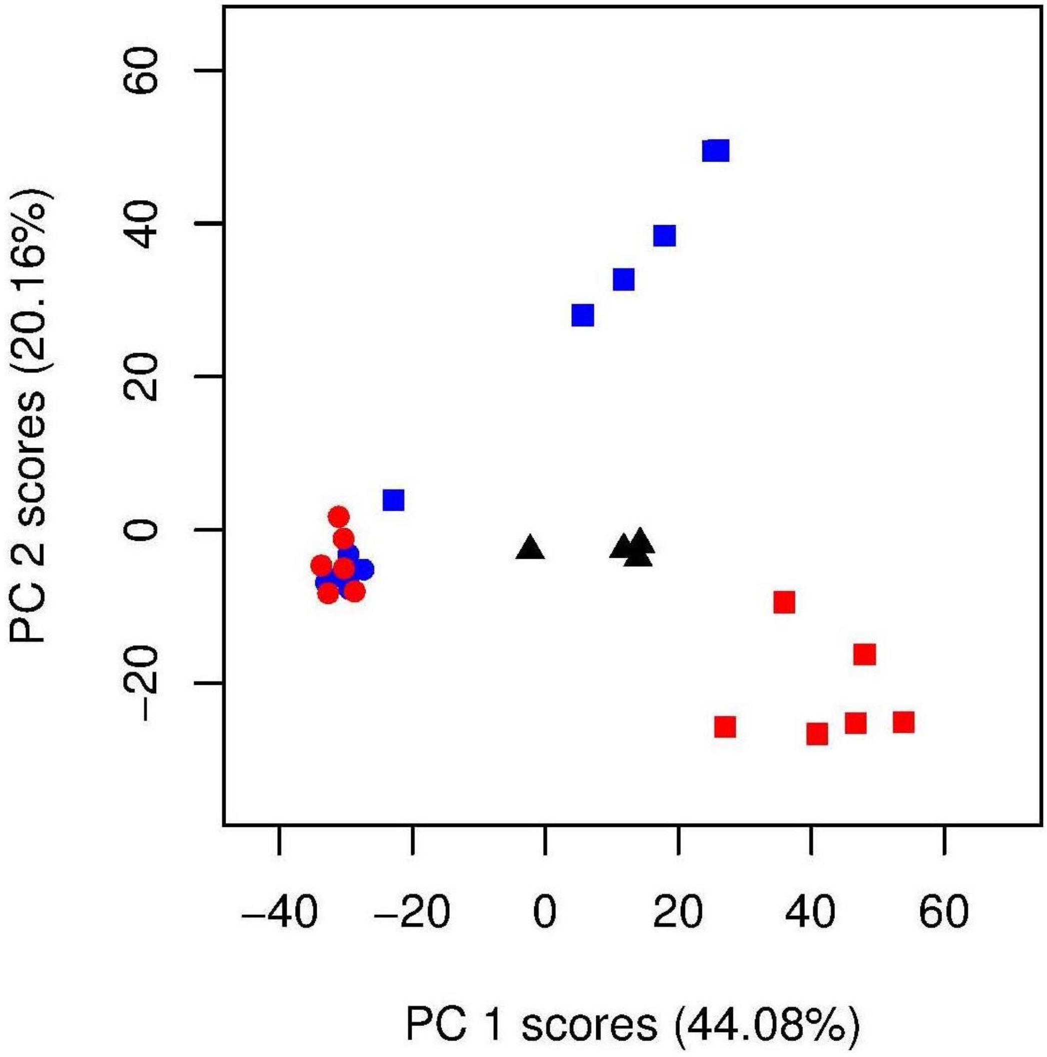

Figure S2. Stable isotope patterns observed in mass spectra collected from ^{13}C-labeled cultures of *Alexandrium tamarense*. Signal intensity template presented in green, additional ^{13}C labeled peaks in black and isotopic peaks observed in mass spectra of non-labeled *Alexandrium tamarense* samples in red. Despite the high sensitivity and mass accuracy of FT-ICR MS, a relatively high number of non-ideal SIPs were found (**a**–**d**); these arose because the signal intensity of partially or fully-labelled compound dropped below the detection level of the FTICR MS, which resulted in missing peaks across the SIP; a single *m/z* peak resulted from two or more metabolites of similar mass, which resulted in an altered isotope intensity profile; some *m/z* features were falsely assigned to a particular SIP in part because of the finite mass accuracy of the FT-ICR; or no all-^{12}C peak or ^{12}C–^{13}C isotope peak pair could be located.

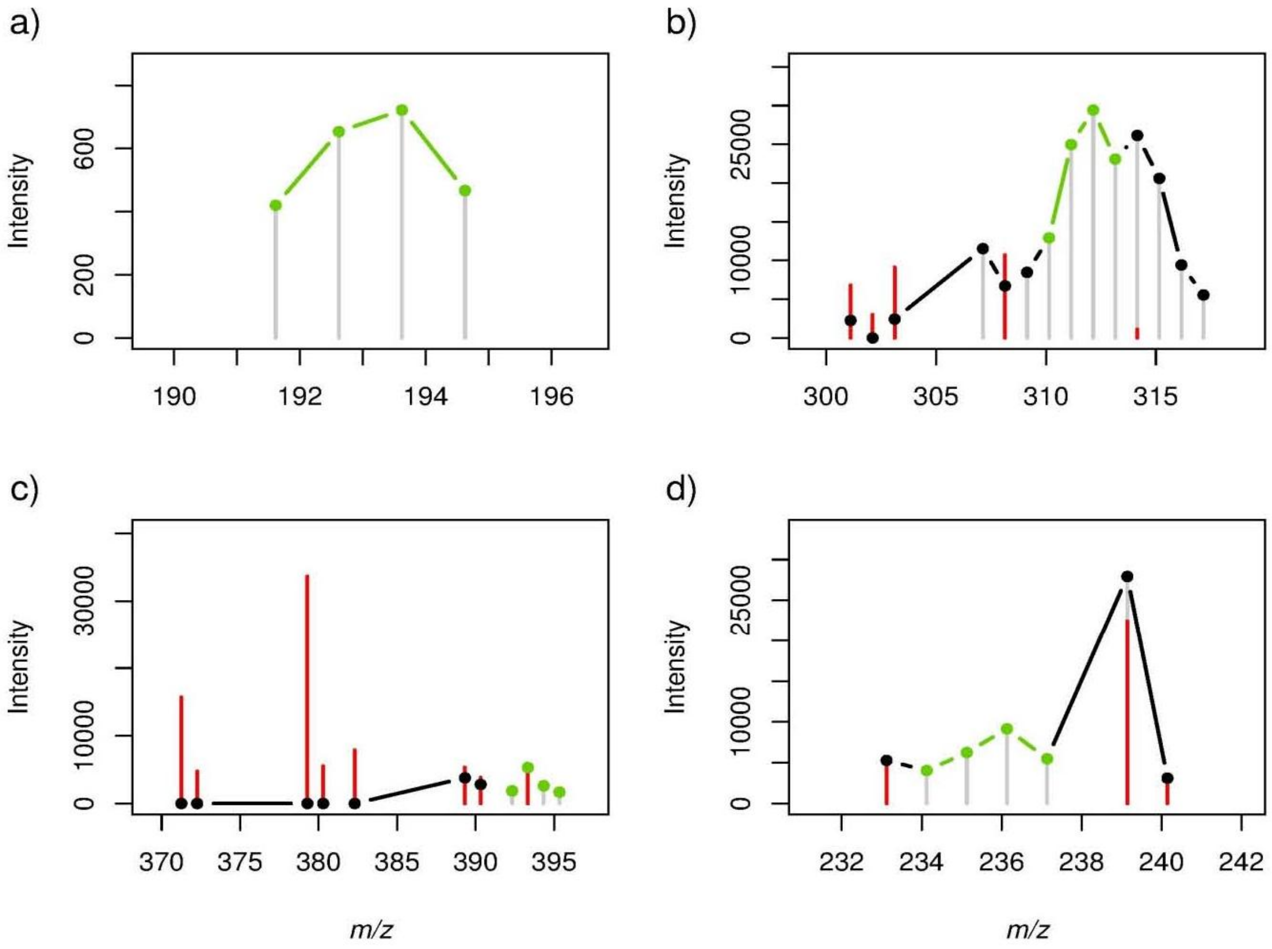

Figure S3. Stable isotope patterns modeled with six different labeling efficiencies (**a**) 1.1%, (**b**) 20%, (**c**) 40% (**d**) 60% (**e**) 80% and (**f**) 100% and four different numbers of ^{12}C atoms in a particular empirical formula: $^{12}C_5H_hN_nO_oP_pS_s$ (black), $^{12}C_{10}H_hN_nO_oP_pS_s$ (blue), $^{12}C_{15}H_hN_nO_oP_pS_s$ (red) and $^{12}C_{20}H_hN_nO_oP_pS_s$ (green). As the labelling efficiency is varied the isotope intensity distribution will move, becoming left-shifted for label efficiencies less than 50% and right-shifted for label efficiencies greater than 50%.

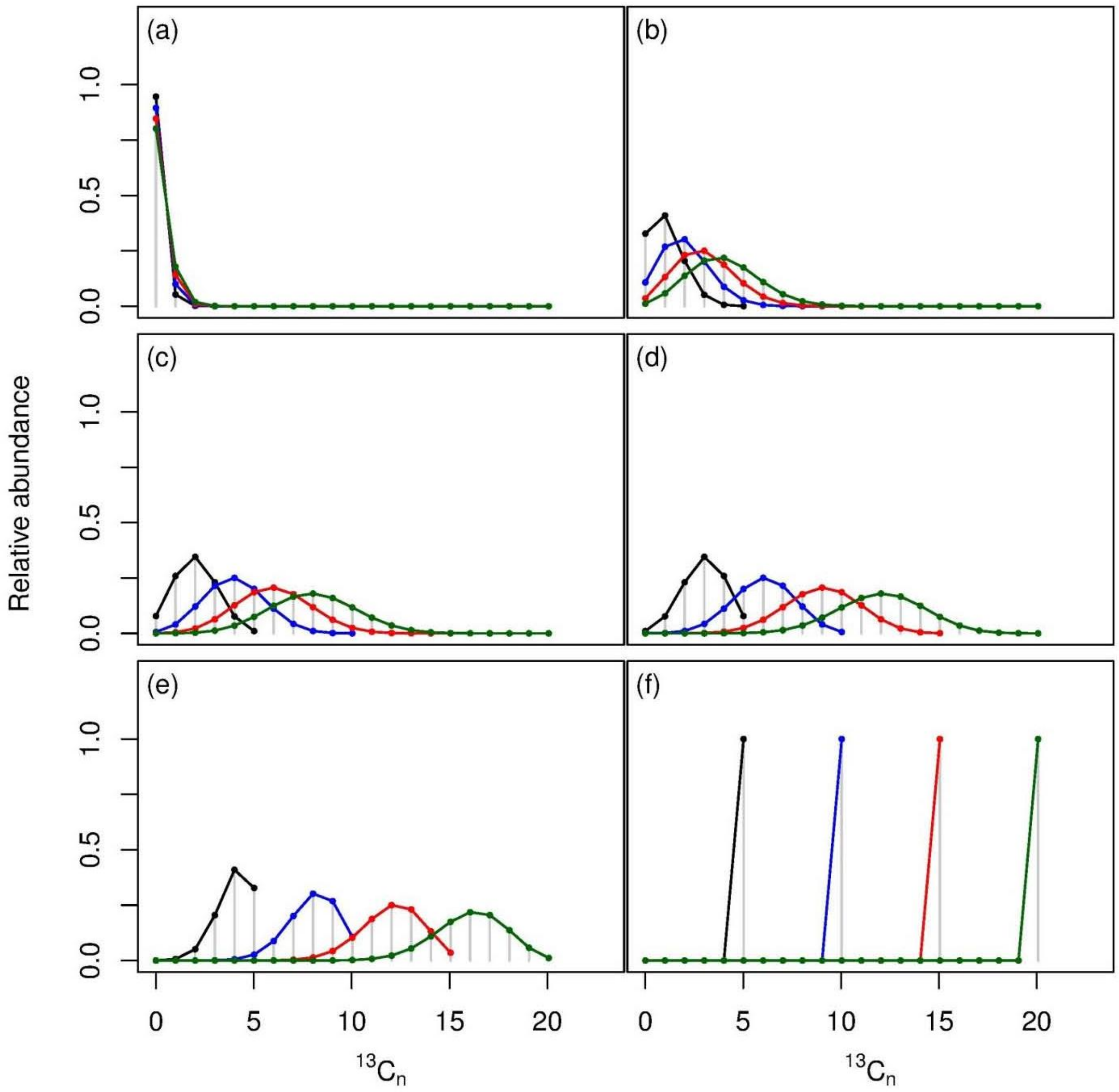

Figure S4. Mass spectral characterization after solid-phase-extraction of the putatively annotated (based upon accurate mass measurement) myristic acid, (**a**) mass spectra for unlabelled fraction FM2 (top) and ^{13}C-labelled FM2 (Orbitrap Velos), showing the isotopic pattern related to the original *m/z* 227.20143 signal. Both the monoisotopic signal and the SIP had their maximum intensities in the same FM2 fractions; (**b**) collision-induced fragmentation (CID) MS/MS spectra (Orbitrap, normalised collision energy of 30%) for the monoisotopic signal *m/z* 227 from the unlabelled FM2 fraction, overlaid with CID spectra for three of the SIP signals from the labelled FM2 fraction, all four SIP signals show a similar pattern of losses, as would be expected from isotopes; (**c**) CID MS/MS spectra (LTQ FT Ultra, normalised collision energy of 45%) for the monoisotopic signal *m/z* 227 from the unlabelled FM2 fraction, compared with that of an authentic myristic acid standard, while the signals in the standard constitute the main fragments in the sample, there is likely another, isomeric compound present.

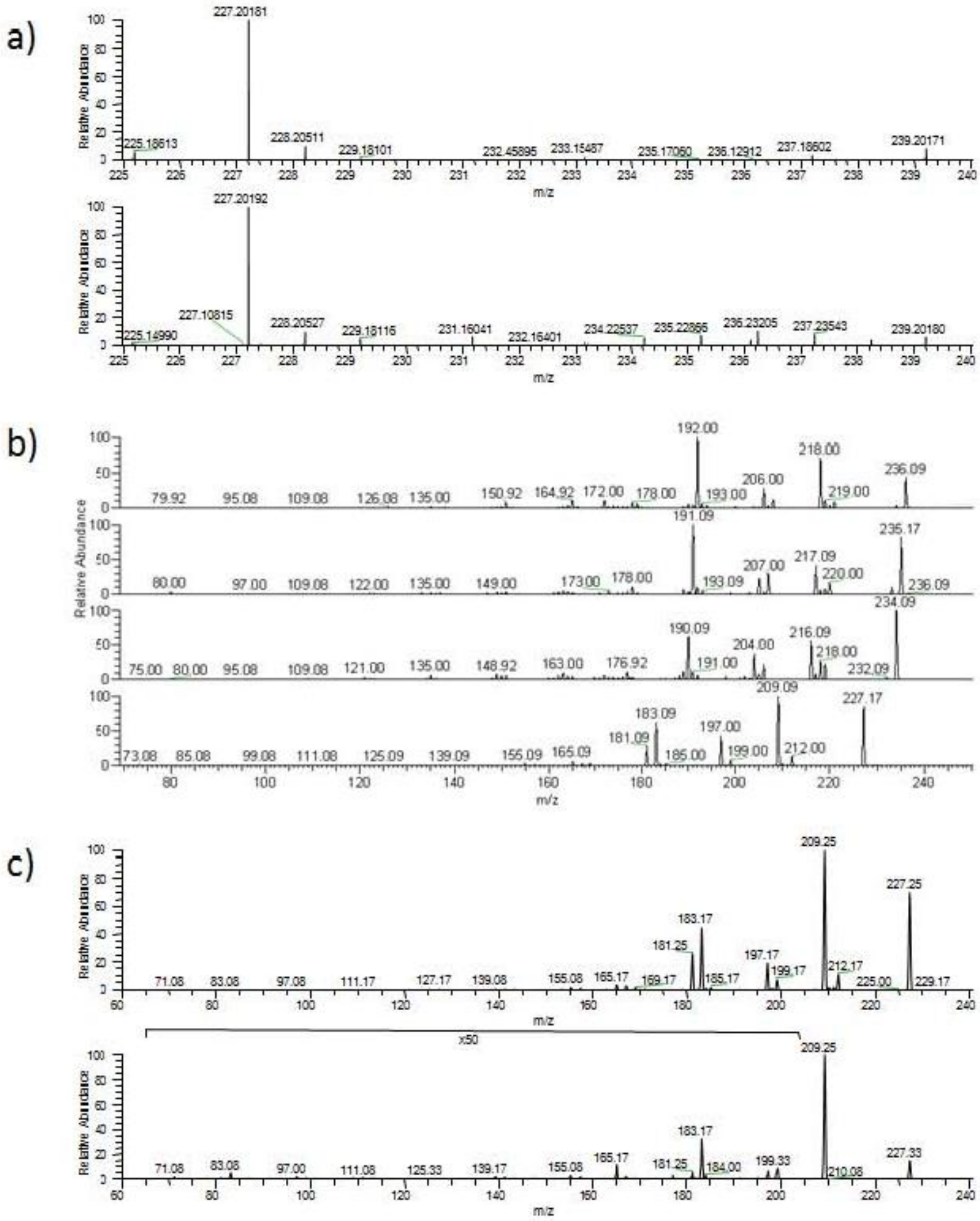

Table S1. Stable isotope patterns located and annotated in mass spectra collected from ^{13}C-labeled cultures of *Alexandrium tamarense*.

m/z	Intensity	ID SIP (Figure S5)	Empirical formula (all-^{12}C containing peak)	Ion form	Mass error (ppm)	KEGG compound	*r*-value	*p*-value
115.04011	2958.4295	1	C_3H_4O	$[M + Acetate]^-$	0.36	[2-Propyn-1-ol, Acrolein]	0.00	1.0000
115.04011	2958.4295	1	$C_5H_8O_3$	$[M - H]^-$	0.36	[2-Oxopentanoic acid, 3-Methyl-2-oxobutanoic acid, 3-Oxopentanoic acid, 5-Oxopentanoate]	0.98	0.0187
121.02940	168682.4333	2	C_5H_2	$[M + Acetate]^-$	−0.86		−0.82	0.3857
121.02940	168682.4333	2	$C_7H_6O_2$	$[M - H]^-$	−0.86	[3-Hydroxybenzaldehyde, 4-Hydroxybenzaldehyde, Benzoate, Salicylaldehyde, Tropolone]	0.98	0.0040
123.04518	3533.979	3	C_5H_4	$[M + Acetate]^-$	0.21		0.30	0.7038
123.04518	3533.979	3	$C_7H_8O_2$	$[M - H]^-$	0.21	[2,3-Dihydroxytoluene, 3-Hydroxybenzyl alcohol, 4-Methylcatechol, Orcinol, Salicyl alcohol, *o*-Methoxyphenol, *p*-Hydroxybenzyl alcohol]	0.95	0.0129
125.06084	3055.881	4	C_5H_6	$[M + Acetate]^-$	0.29		0.27	0.7321
125.06084	3055.881	4	$C_7H_{10}O_2$	$[M - H]^-$	0.29	[Cyclohex-1-enecarboxylic acid, Toluene-*cis*-dihydrodiol]	0.98	0.0007
125.09722	7374.999	5	$C_8H_{14}O$	$[M - H]^-$	0.25	[Sulcatone]	0.99	0.0008
127.04010	5328.0675	6	C_4H_4O	$[M + Acetate]^-$	0.25	[3-Butyn-1-al, Furan]	0.16	0.8967
127.04010	5328.0675	6	$C_6H_8O_3$	$[M - H]^-$	0.25	[(4*E*)-2-Oxohexenoic acid, 2-Hydroxy-*cis*-hex-2,4-dienoate, Dihydro-4,4-dimethyl-2,3-Furandione, Dihydrophloroglucinol]	0.96	0.0107
127.07647	6996.667667	7	C_5H_8	$[M + Acetate]^-$	0.13	[Isoprene]	0.38	0.6186
127.07647	6996.667667	7	$C_7H_{12}O_2$	$[M - H]^-$	0.13	[1-Oxa-2-oxo-3-methylcycloheptane, 3-Isopropylbut-3-enoic acid, Butyl acrylate, Cyclohexane-1-carboxylate, Ethyl 2-methylbut-2-enoate, alpha-EMGBL, beta-EMGBL]	0.95	0.0139
129.05574	18106.15833	8	C_4H_6O	$[M + Acetate]^-$	0.17	[3-Butyn-1-ol, Crotonaldehyde, Vinyl ether]	0.08	0.9517

Table S1. *Cont.*

m/z	Intensity	ID SIP (Figure S5)	Empirical formula (all-^{12}C containing peak)	Ion form	Mass error (ppm)	KEGG compound	*r*-value	*p*-value
129.05574	18106.15833	8	$C_6H_{10}O_3$	$[M - H]^-$	0.17	[(3*R*)-3-Methyl-2-oxopentanoic acid, (*R*)-Pantolactone, (*S*)-3-Methyl-2-oxopentanoic acid, 1-Oxa-2-oxo-3-hydroxycycloheptane, 2-Hydroxyethyl methacrylate, 2-Oxohexanoic acid, 3-Methyl-2-oxopentanoate, 3-Oxohexanoic acid, 4-Methyl-2-oxopentanoate, 5-Oxohexanoic acid, 6-Hydroxyhexan-6-olide, Adipate semialdehyde, Ethyl 3-oxobutanoate, beta-Ketoisocaproate]	0.93	0.0225
129.09196	329766.9	9	C_5H_{10}	$[M + Acetate]^-$	−1.11		−0.80	0.4075
129.09196	329766.9	9	$C_7H_{14}O_2$	$[M - H]^-$	−1.11	[Ethyl isovalerate, Heptanoic acid, Isoamyl acetate]	0.81	0.1925
131.07140	7190.720167	10	C_4H_8O	$[M + Acetate]^-$	0.24	[1,2-Epoxybutane, Butanal, Methyl ethyl ketone]	0.27	0.8247
131.07140	7190.720167	10	$C_6H_{12}O_3$	$[M - H]^-$	0.24	[6-Hydroxyhexanoic acid, D-2-Hydroxyisocaproate, Ethyl (*R*)-3-hydroxybutanoate, Paraldehyde]	0.91	0.0297
133.06593	6023.855167	11	$C_9H_{10}O$	$[M - H]^-$	0.31	[Chavicol, Cinnamyl alcohol, Indan-1-ol, Phenylacetone]	0.98	0.0215
136.04044	5200.330167	12	C_5H_{3N}	$[M + Acetate]^-$	0.27		−0.83	0.3712
136.04044	5200.330167	12	$C_7H_{7N}O_2$	$[M - H]^-$	0.27	[3-Nitrotoluene, 4-Aminobenzoate, 4-Nitrotoluene, Anthranilate, N-Methylnicotinate]	0.75	0.2476
135.08158	2867.2545	13	$C_9H_{12}O$	$[M - H]^-$	0.31	[4-Propylphenol]	0.97	0.0071
137.06085	4163.286667	14	C_6H_6	$[M + Acetate]^-$	0.34	[Benzene]	0.71	0.1797

Table S1. *Cont.*

m/z	Intensity	ID SIP (Figure S5)	Empirical formula (all-^{12}C containing peak)	Ion form	Mass error (ppm)	KEGG compound	*r*-value	*p*-value
137.06085	4163.286667	14	$C_8H_{10}O_2$	$[M - H]^-$	0.34	[1-(4-Hydroxyphenyl)ethanol, 2-Methyl-6-oxohepta-2,4-dienal, 3-Ethylcatechol, 3-Methoxybenzyl alcohol, 4-Hydroxyphenylethanol, Styrene *cis*-glycol]	0.88	0.0215
137.09722	2682.751667	15	$C_9H_{14}O$	$[M - H]^-$	0.23	[3,6-Nonadienal, Isophorone, Nona-2,6-dienal]	0.90	0.0363
139.07650	5189.27	16	C_6H_8	$[M + Acetate]^-$	0.33		0.72	0.1739
139.07650	5189.27	16	$C_8H_{12}O_2$	$[M - H]^-$	0.33	[2-Propyl-2,4-pentadienoic acid, 4-Vinylcyclohexene diepoxide, 7-Hydroxy-6-methylhepta-3,5-dienal, *cis*-1,2-Dihydro-3-ethylcatechol]	0.86	0.0262
141.05562	6652.611333	17	C_5H_6O	$[M + Acetate]^-$	−0.7		0.93	0.0731
141.05562	6652.611333	17	$C_7H_{10}O_3$	$[M - H]^-$	−0.7	[4-Oxocyclohexanecarboxylate]	0.57	0.4279
141.09206	28963.815	18	C_6H_{10}	$[M + Acetate]^-$	−0.31		0.75	0.1426
141.09206	28963.815	18	$C_8H_{14}O_2$	$[M - H]^-$	−0.31	[2-*n*-Propyl-2-pentenoic acid, 2-*n*-Propyl-3-pentenoic acid, 2-*n*-Propyl-4-pentenoic acid, Allyl isovalerate, Cycloheptanecarboxylic acid, Cyclohexyl acetate]	0.76	0.0781
144.04555	5955.535	19	C_9H_7NO	$[M - H]^-$	0.43	[1(2*H*)-Isoquinolinone, 3-Methyleneoxindole, 8-Hydroxyquinoline, Indole-3-carboxaldehyde, Quinolin-2-ol, Quinolin-4-ol]	0.88	0.0216
185.11855	1751.43895	20	$C_8H_{14}O$	$[M + Acetate]^-$	1.25	[Sulcatone]	−0.09	0.9149

Table S1. *Cont.*

m/z	Intensity	ID SIP (Figure S5)	Empirical formula (all-^{12}C containing peak)	Ion form	Mass error (ppm)	KEGG compound	*r*-value	*p*-value
185.11855	1751.43895	20	$C_{10}H_{18}O_3$	[M − H]$^-$	1.25	[(3*R*)-6-Hydroxy-3-isopropenyl-heptanoate, (3*S*)-6-Hydroxy-3-isopropenyl-heptanoate, (5*R*)-6-Hydroxy-5-isopropenyl-2-methylhexanoate, (5*S*)-6-Hydroxy-5-isopropenyl-2-methylhexanoate, 10-Oxodecanoate, 2-Oxodecanoic acid, Epomediol]	0.95	0.0115
189.07708	8847.3296	21	$C_6H_{10}O_3$	[M + Acetate]$^-$	1.22	[(3*R*)-3-Methyl-2-oxopentanoic acid, (*R*)-Pantolactone, (*S*)-3-Methyl-2-oxopentanoic acid, 1-Oxa-2-oxo-3-hydroxycycloheptane, 2-Hydroxyethyl methacrylate, 2-Oxohexanoic acid, 3-Methyl-2-oxopentanoate, 3-Oxohexanoic acid, 4-Methyl-2-oxopentanoate, 5-Oxohexanoic acid, 6-Hydroxyhexan-6-olide, Adipate semialdehyde, Ethyl 3-oxobutanoate, beta-Ketoisocaproate]	0.74	0.2634
189.07708	8847.3296	21	$C_8H_{14}O_5$	[M − H]$^-$	1.22	[(*R*)-3-((*R*)-3-Hydroxybutanoyloxy)butanoate]	−0.01	0.9887
189.07708	8847.3296	21	$C_3H_{11}N_8P$	[M − H]$^-$	−0.39		0.00	1.0000
187.13422	70169.226	22	$C_8H_{16}O$	[M + Acetate]$^-$	1.34	[1-Octanal, 1-Octen-3-ol, 3-Octanone, Sulcatol]	−0.15	0.8148
187.13422	70169.226	22	$C_{10}H_{20}O_3$	[M − H]$^-$	1.34	[10-Hydroxydecanoic acid, 6-Hydroxy-3,7-dimethyloctanoate]	0.98	0.0001
191.09272	7760.51095	23	$C_6H_{12}O_3$	[M + Acetate]$^-$	1.16	[6-Hydroxyhexanoic acid, D-2-Hydroxyisocaproate, Ethyl (*R*)-3-hydroxybutanoate, Paraldehyde]	0.10	0.8956
191.09272	7760.51095	23	$C_8H_{16}O_5$	[M − H]$^-$	1.16	[D-Mycinose]	0.75	0.1466
191.09272	7760.51095	23	$C_3H_{13}N_8P$	[M − H]$^-$	−0.44		0.00	1.0000
197.11853	114412.8083	24	$C_9H_{14}O$	[M + Acetate]$^-$	1.07	[3,6-Nonadienal, Isophorone, Nona-2,6-dienal]	0.60	0.2038
197.11853	114412.8083	24	$C_{11}H_{18}O_3$	[M − H]$^-$	1.07		0.96	0.0007

Table S1. *Cont.*

m/z	Intensity	ID SIP (Figure S5)	Empirical formula (all-^{12}C containing peak)	Ion form	Mass error (ppm)	KEGG compound	*r*-value	*p*-value
199.09782	69691.88	25	$C_8H_{12}O_2$	[M + Acetate]$^-$	1.19	[2-Propyl-2,4-pentadienoic acid, 4-Vinylcyclohexene diepoxide, 7-Hydroxy-6-methylhepta-3,5-dienal, *cis*-1,2-Dihydro-3-ethylcatechol]	0.67	0.2200
199.09782	69691.88	25	$C_{10}H_{16}O_4$	[M − H]$^-$	1.19		0.88	0.0212
197.15491	223653.7283	26	$C_{10}H_{18}$	[M + Acetate]$^-$	1.05		0.58	0.1735
197.15491	223653.7283	26	$C_{12}H_{22}O_2$	[M − H]$^-$	1.05	[(−)-Menthyl acetate, Citronellyl acetate, Decanoyl acetaldehyde, Neomenthyl acetate]	1.00	0.0000
199.13419	149139.7433	27	$C_9H_{16}O$	[M + Acetate]$^-$	1.11		0.47	0.3456
199.13419	149139.7433	27	$C_{11}H_{20}O_3$	[M − H]$^-$	1.11		0.99	0.0000
201.11338	506046.7367	28	$C_8H_{14}O_2$	[M + Acetate]$^-$	0.73	[2-*n*-Propyl-2-pentenoic acid, 2-*n*-Propyl-3-pentenoic acid, 2-*n*-Propyl-4-pentenoic acid, Allyl isovalerate, Cycloheptanecarboxylic acid, Cyclohexyl acetate]	0.72	0.1699
201.11338	506046.7367	28	$C_{10}H_{18}O_4$	[M − H]$^-$	0.73	[Diethyl adipate, Dimethyl suberate, Sebacic acid]	0.72	0.1668
199.17039	4947357.983	29	$C_{10}H_{20}$	[M + Acetate]$^-$	0.18		0.75	0.1478
199.17039	4947357.983	29	$C_{12}H_{24}O_2$	[M − H]$^-$	0.18	[Dodecanoic acid]	0.96	0.0085
227.20143	1634653.517	30	$C_{12}H_{24}$	[M + Acetate]$^-$	−0.98		0.54	0.1365
227.20143	1634653.517	30	$C_{14}H_{28}O_2$	[M − H]$^-$	−0.98	[Tetradecanoic acid]	0.98	0.0000
229.14431	40693.77	31	$C_{10}H_{18}O_2$	[M + Acetate]$^-$	−0.98	[(+)-Neomatatabiol, (1*R*,2*R*,4*S*)-Limonene-1,2-diol, (4*R*,7*S*)-7-Isopropyl-4-methyloxepan-2-one, (*E*)-3,7-Dimethylocta-1,6-diene-3,8-diol, 10-Hydroxygeraniol, 6-endo-Hydroxycineole, 7-Isopropyl-4-methyloxepan-2-one, 8-Methyl-6-nonenoic acid, Citronellate, Limonene-1,2-diol, *p*-Menth-8-ene-1,2-diol]	0.55	0.3337

Table S1. *Cont.*

m/z	Intensity	ID SIP (Figure S5)	Empirical formula (all-^{12}C containing peak)	Ion form	Mass error (ppm)	KEGG compound	*r*-value	*p*-value
229.14431	40693.77	31	$C_{12}H_{22}O_4$	$[M-H]^-$	−0.98	[Dibutyl succinate, Diisopropyl adipate, Dodecanedioic acid]	0.95	0.0141
231.15994	36684.80333	32	$C_{10}H_{20}O_2$	$[M+Acetate]^-$	−1.05	[Decanoic acid, Ethyl octanoate, Isoamyl isovalerate, *p*-Menthane-3,8-diol]	0.60	0.4048
231.15994	36684.80333	32	$C_{12}H_{24}O_4$	$[M-H]^-$	−1.05		0.78	0.2196
239.12856	669303	33	$C_{11}H_{16}O_2$	$[M+Acetate]^-$	−1.35	[3-tert-Butyl-5-methylcatechol]	0.89	0.0071
239.12856	669303	33	$C_{13}H_{20}O_4$	$[M-H]^-$	−1.35		0.89	0.0027
235.13374	70891.09	34	$C_{12}H_{16}O$	$[M+Acetate]^-$	−0.97		0.75	0.0516
235.13374	70891.09	34	$C_{14}H_{20}O_3$	$[M-H]^-$	−0.97	[4-Heptyloxybenzoic acid, Heptyl *p*-hydroxybenzoate]	0.93	0.0021
237.18575	201365.8677	35	$C_{13}H_{22}$	$[M+Acetate]^-$	−1.07		0.34	0.5042
237.18575	201365.8677	35	$C_{15}H_{26}O_2$	$[M-H]^-$	−1.07	[Bisabolol oxide A, Bisabolol oxide B, Bornyl isovalerate, Centarol, Daucol, Debneyol, Kanokonol, Seiricardine A]	0.94	0.0052
237.14936	87379.51167	36	$C_{12}H_{18}O$	$[M+Acetate]^-$	−1.09	[4-*n*-Hexylphenol, Propofol]	0.87	0.0570
237.14936	87379.51167	36	$C_{14}H_{22}O_3$	$[M-H]^-$	−1.09		0.87	0.0531
239.16501	191326.915	37	$C_{12}H_{20}O$	$[M+Acetate]^-$	−1.08	[(*Z*,*Z*,*Z*)-3,6,9-Dodecatrien-1-ol]	0.68	0.2038
239.16501	191326.915	37	$C_{14}H_{24}O_3$	$[M-H]^-$	−1.08		0.98	0.0025
239.20140	479360.08	38	$C_{13}H_{24}$	$[M+Acetate]^-$	−1.06		0.83	0.0422
239.20140	479360.08	38	$C_{15}H_{28}O_2$	$[M-H]^-$	−1.06	[Cryptomeridiol]	0.92	0.0103
241.14428	145457.2133	39	$C_{11}H_{18}O_2$	$[M+Acetate]^-$	−1.05	[Decahydro-2-naphthoic acid, Geranyl formate]	0.87	0.0558
241.14428	145457.2133	39	$C_{13}H_{22}O_4$	$[M-H]^-$	−1.05		0.66	0.2250
243.19631	194249.7617	40	$C_{12}H_{24}O$	$[M+Acetate]^-$	−1.06	[Dodecylaldehyde]	0.48	0.5153
243.19631	194249.7617	40	$C_{14}H_{28}O_3$	$[M-H]^-$	−1.06	[2*S*-Hydroxytetradecanoic acid]	0.83	0.1742
243.13889	64398.36167	41	$C_{14}H_{16}$	$[M+Acetate]^-$	−0.67	[Chamazulene]	0.79	0.0601
243.13889	64398.36167	41	$C_{16}H_{20}O_2$	$[M-H]^-$	−0.67	[Arnebinol, Geranylbenzoquinone]	0.58	0.2246

Table S1. *Cont.*

m/z	Intensity	ID SIP (Figure S5)	Empirical formula (all-^{12}C containing peak)	Ion form	Mass error (ppm)	KEGG compound	*r*-value	*p*-value
253.21701	472387.4833	42	$C_{14}H_{26}$	[M + Acetate]$^-$	–1.16		0.94	0.0049
253.21701	472387.4833	42	$C_{16}H_{30}O_2$	[M − H]$^-$	–1.16	[(9*Z*)-Hexadecenoic acid]	0.70	0.1189
255.23266	3133035	43	$C_{14}H_{28}$	[M + Acetate]$^-$	–1.15		0.81	0.0041
255.23266	3133035	43	$C_{16}H_{32}O_2$	[M − H]$^-$	–1.15	[Hexadecanoic acid]	0.99	0.0000
295.26402	33745.72783	44	$C_{17}H_{32}$	[M + Acetate]$^-$	–0.79		0.59	0.1199
295.26402	33745.72783	44	$C_{19}H_{36}O_2$	[M − H]$^-$	–0.79	[17-Methyl-6*Z*-octadecenoic acid, Lactobacillic acid, Oleic acid methyl ester]	0.99	0.0000
295.20651	7720.9735	45	$C_{21}H_{28}O$	[M − H]$^-$	–0.77		0.76	0.0779
301.11114	6797.914	46	$C_{14}H_{15}N_2P$	[M + Acetate]$^-$	0		−0.46	0.2081
301.11114	6797.914	46	$C_4H_{14}N_6O_6$	[M + Acetate]$^-$	–0.66		0.00	1.0000
301.11114	6797.914	46	$C_{12}H_{18}O_3S$	[M + Acetate]$^-$	–1.26		−0.92	0.0036
301.11114	6797.914	46	$C_{17}H_{18}N_2O$	[M + Cl]$^-$	–0.58	[Borreline]	0.71	0.0135
301.11114	6797.914	46	$C_{16}H_{19}N_2O_2P$	[M − H]$^-$	0		0.48	0.1341
301.11114	6797.914	46	$C_6H_{18}N_6O_8$	[M − H]$^-$	–0.66		0.00	1.0000
301.11114	6797.914	46	$C_{14}H_{22}O_5S$	[M − H]$^-$	–1.26		−0.46	0.2081
297.13409	24117.57167	47	$C_{13}H_{18}O_4$	[M + Acetate]$^-$	–0.92		−0.28	0.7183
297.13409	24117.57167	47	$C_{10}H_{27}N_4PS_2$	[M − H]$^-$	–0.38		0.00	1.0000
297.13409	24117.57167	47	$C_{15}H_{22}O_6$	[M − H]$^-$	–0.92	[Sesamex]	0.83	0.0387
301.14728	3699.104667	48	$C_5H_{18}N_6O_5$	[M + Acetate]$^-$	–1.47		0.00	1.0000
301.14728	3699.104667	48	$C_{10}H_{26}N_4O_2S$	[M + Cl]$^-$	0.77		0.92	0.2588
301.14728	3699.104667	48	$C_{18}H_{22}N_2$	[M + Cl]$^-$	–1.39	[Cyclizine, Desipramine]	−0.69	0.1258
301.14728	3699.104667	48	$C_{17}H_{23}N_2OP$	[M − H]$^-$	–0.81		−0.37	0.4675
301.14728	3699.104667	48	$C_7H_{22}N_6O_7$	[M − H]$^-$	–1.47		0.00	1.0000
301.12090	20234.44	49	$C_{12}H_{19}O_3P$	[M + Acetate]$^-$	–0.45		0.25	0.5862

Table S1. *Cont.*

m/z	Intensity	ID SIP (Figure S5)	Empirical formula (all-^{12}C containing peak)	Ion form	Mass error (ppm)	KEGG compound	*r*-value	*p*-value
301.12090	20234.44	49	$C_{15}H_{22}O_4$	$[M + Cl]^-$	–1.03	[Mukaadial, Rugosal, Xanthoxic acid, Zinniol]	0.94	0.0015
301.12090	20234.44	49	$C_{17}H_{14}N_6$	$[M - H]^-$	0.61		0.51	0.2378
301.12090	20234.44	49	$C_{14}H_{23}O_5P$	$[M - H]^-$	–0.45		0.87	0.0106
301.12090	20234.44	49	$C_9H_{20}N_8P_2$	$[M - H]^-$	–1.47		−0.95	0.0535
303.23274	92459.4405	50	$C_{18}H_{28}$	$[M + Acetate]^-$	–0.7		0.70	0.0778
303.23274	92459.4405	50	$C_{20}H_{32}O_2$	$[M - H]^-$	–0.7	[(5*Z*,8*Z*,11*Z*,14*Z*)-Icosatetraenoic acid, 17-Methyl-5alpha-androst-2-ene-1alpha,17beta-diol, 17beta-Hydroxy-4alpha-methyl-5alpha-androstan-3-one, 2-Ketoepimanool, 3alpha-Hydroxy-2alpha-methyl-5alpha-androstan-17-one, 3beta-Methoxyandrost-5-en-16beta-ol, Acutilol A, Dihydroabietic acid, Methandriol, Metholone, Taxa-4(20),11(12)-dien-5alpha,13alpha-diol]	0.57	0.1861
307.11366	161073.0933	51	$C_6H_8N_{12}$	$[M + Acetate]^-$	1.04		0.00	1.0000
307.11366	161073.0933	51	$C_{11}H_{21}O_2PS$	$[M + Acetate]^-$	–0.6		−0.58	0.2305
307.11366	161073.0933	51	$C_{16}H_{21}N_2P$	$[M + Cl]^-$	0.07		0.68	0.0638
307.11366	161073.0933	51	$C_6H_{20}N_6O_6$	$[M + Cl]^-$	–0.57		0.00	1.0000
307.11366	161073.0933	51	$C_{14}H_{24}O_3S$	$[M + Cl]^-$	–1.17		0.34	0.4127
307.11366	161073.0933	51	$C_8H_{12}N_{12}O_2$	$[M - H]^-$	1.04		−0.67	0.5343
307.11366	161073.0933	51	$C_{15}H_{22}N_2OP_2$	$[M - H]^-$	0.64		0.53	0.1723
307.11366	161073.0933	51	$C_{13}H_{25}O_4PS$	$[M - H]^-$	–0.6		0.12	0.7836
311.12652	15892.83667	52	$C_{13}H_{12}N_6$	$[M + Acetate]^-$	1.04		0.24	0.6477
311.12652	15892.83667	52	$C_{10}H_{21}O_5P$	$[M + Acetate]^-$	0.01		0.89	0.0179
311.12652	15892.83667	52	$C_5H_{18}N_8P_2$	$[M + Acetate]^-$	–0.97		0.00	1.0000
311.12652	15892.83667	52	$C_{13}H_{24}O_6$	$[M + Cl]^-$	–0.55		0.24	0.6477

Table S1. *Cont.*

m/z	Intensity	ID SIP (Figure S5)	Empirical formula (all-^{12}C containing peak)	Ion form	Mass error (ppm)	KEGG compound	*r*-value	*p*-value
311.12652	15892.83667	52	$C_{15}H_{16}N_6O_2$	[M − H]⁻	1.04		−0.11	0.8313
311.12652	15892.83667	52	$C_{12}H_{25}O_7P$	[M − H]⁻	0.01		0.52	0.2900
311.12652	15892.83667	52	$C_7H_{22}N_8O_2P_2$	[M − H]⁻	–0.97		−0.96	0.0363
311.12652	15892.83667	52	$C_8H_{16}N_{12}S$	[M − H]⁻	–1.17		−0.38	0.5302
311.22252	230176.5167	53	$C_{16}H_{28}O_2$	[M + Acetate]⁻	–0.85	[Hydnocarpic acid]	0.73	0.0113
311.22252	230176.5167	53	$C_{18}H_{32}O_4$	[M − H]⁻	–0.85	[(7*S*,8*S*)-DiHODE, (9*Z*,11*E*)-(13*S*)-13-Hydroperoxyoctadeca-9,11-dienoic acid, (9*Z*,12*Z*)-(11*S*)-11-Hydroperoxyoctadeca-9,12-dienoic acid, 12,13-Epoxy-9-hydroxy-10-octadecenoate, 8(*R*)-HPODE, 9(*S*)-HPODE', 9,10-12,13-Diepoxyoctadecanoate, 9,10-Epoxy-13-hydroxy-11-octadecenoate]	0.99	0.0000
313.23816	206802.6367	54	$C_{16}H_{30}O_2$	[M + Acetate]⁻	–0.87	[(9*Z*)-Hexadecenoic acid]	0.99	0.0000
313.23816	206802.6367	54	$C_{18}H_{34}O_4$	[M − H]⁻	–0.87	[(9*Z*)-(7*S*,8*S*)-Dihydroxyoctadecenoic acid, 12,13-DHOME, 9,10-DHOME]	0.90	0.0000
317.11583	32528.65833	55	$C_{12}H_{19}O_4P$	[M + Acetate]⁻	–0.38		0.50	0.3151
317.11583	32528.65833	55	$C_{15}H_{22}O_5$	[M + Cl]⁻	–0.94	[Dihydrophaseic acid, Hymenoxon, Qing Hau Sau]	−0.15	0.7821
317.11583	32528.65833	55	$C_{17}H_{14}N_6O$	[M − H]⁻	0.62		−0.33	0.5283
317.11583	32528.65833	55	$C_{14}H_{23}O_6P$	[M − H]⁻	–0.38		0.00	0.9977
317.11583	32528.65833	55	$C_9H_{20}N_8OP_2$	[M − H]⁻	–1.35		0.72	0.1073
323.09025	17448.7	56	$C_{13}H_8N_6O$	[M + Acetate]⁻	1.35		0.24	0.6093
323.09025	17448.7	56	$C_{10}H_{17}O_6P$	[M + Acetate]⁻	0.37		−0.55	0.1966
323.09025	17448.7	56	$C_5H_{14}N_8OP_2$	[M + Acetate]⁻	–0.58		0.00	1.0000
323.09025	17448.7	56	$C_{13}H_{20}O_7$	[M + Cl]⁻	–0.18		0.24	0.6093
323.09025	17448.7	56	$C_8H_{17}N_8O_2P$	[M + Cl]⁻	–1.12		−0.67	0.2123

Table S1. *Cont.*

m/z	Intensity	ID SIP (Figure S5)	Empirical formula (all-^{12}C containing peak)	Ion form	Mass error (ppm)	KEGG compound	*r*-value	*p*-value
323.09025	17448.7	56	$C_{15}H_{12}N_6O_3$	$[M - H]^-$	1.35		0.59	0.1670
323.09025	17448.7	56	$C_{23}H_{16}S$	$[M - H]^-$	0.79		0.87	0.0113
323.09025	17448.7	56	$C_8H_{20}N_8S_3$	$[M - H]^-$	0.68		−0.67	0.2123
323.09025	17448.7	56	$C_{12}H_{21}O_8P$	$[M - H]^-$	0.37		−0.06	0.8995
323.09025	17448.7	56	$C_7H_{18}N_8O_3P_2$	$[M - H]^-$	−0.58		−0.92	0.0769
323.09025	17448.7	56	$C_8H_{12}N_{12}OS$	$[M - H]^-$	−0.77		−0.67	0.2123
323.09025	17448.7	56	$C_{15}H_{22}N_2P_2S$	$[M - H]^-$	−1.14		0.59	0.1670
329.17526	165026.9233	57	$C_{12}H_{23}N_4OP$	$[M + Acetate]^-$	1.39		0.73	0.1630
329.17526	165026.9233	57	$C_{15}H_{26}N_4O_2$	$[M + Cl]^-$	0.86		0.23	0.6550
329.17526	165026.9233	57	$C_8H_{26}N_{10}S$	$[M + Cl]^-$	−1.23		0.00	1.0000
329.17526	165026.9233	57	$C_{14}H_{27}N_4O_3P$	$[M - H]^-$	1.39		0.46	0.3587
329.17526	165026.9233	57	$C_7H_{27}N_{10}OPS$	$[M - H]^-$	−0.69		0.00	1.0000
335.22239	195300.676	58	$C_{18}H_{28}O_2$	$[M + Acetate]^-$	−1.17	[Kinoprene, Stearidonic acid]	0.24	0.6521
335.22239	195300.676	58	$C_{15}H_{32}N_4O_2$	$[M + Cl]^-$	1.38		0.92	0.0087
335.22239	195300.676	58	$C_{20}H_{32}O_4$	$[M - H]^-$	−1.17	[(13*E*)-(15*S*)-15-Hydroxy-9-oxoprosta-10,13-dienoate, (13*E*)-(15*S*)-15-Hydroxy-9-oxoprosta-11,13-dienoate, (5*Z*,9*E*,14*Z*)-(8xi,11*R*,12*S*)-11,12-Epoxy-8-hydroxyicosa-5,9,14-trienoic acid, 11(*R*)-HPETE, 11H-14,15-EETA, 12(*R*)-HPETE, 12(*S*)-HPETE, 15(*S*)-HPETE, 15H-11,12-EETA, 5(*S*)-HPETE, 8(*R*)-HPETE, 8(*S*)-HPETE, 9(*S*)-HPETE, Diterpenoid SP-II, Hepoxilin A3, Hepoxilin B3, Leukotriene B4, Portulal, Prostaglandin B1]	−0.07	0.8882

Table S1. *Cont.*

m/z	Intensity	ID SIP (Figure S5)	Empirical formula (all-^{12}C containing peak)	Ion form	Mass error (ppm)	KEGG compound	*r*-value	*p*-value
339.25374	83610.57833	59	$C_{18}H_{32}O_2$	[M + Acetate]$^-$	−1.01	[9-*cis*,11-*trans*-Octadecadienoate, Chaulmoogric acid, Linoleate, Malvalic acid, Stearolic acid]	0.96	0.0000
339.25374	83610.57833	59	$C_{20}H_{36}O_4$	[M − H]$^-$	−1.01		0.91	0.0001
367.21253	38839.35667	60	$C_{18}H_{28}O_4$	[M + Acetate]$^-$	−0.23	[5-*O*-Methylembelin, Soraphen O]	0.83	0.0008
367.21253	38839.35667	60	$C_{15}H_{37}N_4PS_2$	[M − H]$^-$	0.21		0.54	0.0676
367.21253	38839.35667	60	$C_{20}H_{32}O_6$	[M − H]$^-$	−0.23	[11-Dehydro-thromboxane B2, 6-Keto-prostaglandin E1, Cinncassiol D2, Cinncassiol D3, Platenolide A, Prostaglandin G2]	0.62	0.0316
367.21253	38839.35667	60	$C_{15}H_{29}N_8OP$	[M − H]$^-$	−1.06		0.54	0.0676
371.22577	56509.405	61	$C_{10}H_{28}N_6O_5$	[M + Acetate]$^-$	−0.55		0.84	0.0182
371.22577	56509.405	61	$C_{18}H_{32}O_2S$	[M + Acetate]$^-$	−1.04		−0.55	0.1593
371.22577	56509.405	61	$C_5H_{25}N_{14}P$	[M + Acetate]$^-$	−1.37		0.00	1.0000
371.22577	56509.405	61	$C_{15}H_{36}N_4O_2S$	[M + Cl]$^-$	1.27		−0.21	0.6247
371.22577	56509.405	61	$C_{23}H_{32}N_2$	[M + Cl]$^-$	−0.48		−0.62	0.0982
371.22577	56509.405	61	$C_{22}H_{33}N_2OP$	[M − H]$^-$	−0.01		−0.62	0.0997
371.22577	56509.405	61	$C_{12}H_{32}N_6O_7$	[M − H]$^-$	−0.55		0.69	0.0558
371.22577	56509.405	61	$C_{20}H_{36}O_4S$	[M − H]$^-$	−1.04		−0.61	0.1120
371.22577	56509.405	61	$C_7H_{29}N_{14}O_2P$	[M − H]$^-$	−1.37		−0.54	0.4650
371.13767	48627.795	62	$C_{14}H_{21}N_2O_4P$	[M + Acetate]$^-$	−0.21		0.98	0.0000
371.13767	48627.795	62	$C_{12}H_{24}O_7S$	[M + Acetate]$^-$	−1.24		0.48	0.2258
371.13767	48627.795	62	$C_{17}H_{24}N_2O_5$	[M + Cl]$^-$	−0.69		0.56	0.1532
371.13767	48627.795	62	$C_{19}H_{16}N_8O$	[M − H]$^-$	0.65		0.21	0.6249
371.13767	48627.795	62	$C_{11}H_{30}N_6P_2S_2$	[M − H]$^-$	0.22		−0.14	0.7618
371.13767	48627.795	62	$C_{16}H_{25}N_2O_6P$	[M − H]$^-$	−0.21		0.77	0.0245

Table S1. *Cont.*

m/z	Intensity	ID SIP (Figure S5)	Empirical formula (all-^{12}C containing peak)	Ion form	Mass error (ppm)	KEGG compound	*r*-value	*p*-value
371.13767	48627.795	62	$C_{11}H_{22}N_{10}OP_2$	$[M-H]^-$	−1.04		−0.14	0.7618
371.13767	48627.795	62	$C_{14}H_{28}O_9S$	$[M-H]^-$	−1.24		0.98	0.0000
371.13767	48627.795	62	$C_{18}H_{32}P_4$	$[M-H]^-$	−1.36		0.36	0.3800
381.22810	224680.45	63	$C_{19}H_{30}O_4$	$[M+Acetate]^-$	−0.43	(8)-Gingerol, Decylubiquinone, Rapanone]	0.85	0.0000
381.22810	224680.45	63	$C_{10}H_{32}N_{12}P_2$	$[M-H]^-$	1.48		0.12	0.7597
381.22810	224680.45	63	$C_{16}H_{39}N_4PS_2$	$[M-H]^-$	0		0.25	0.3607
381.22810	224680.45	63	$C_{21}H_{34}O_6$	$[M-H]^-$	−0.43	[Sarcostin]	0.96	0.0000
381.22810	224680.45	63	$C_{16}H_{31}N_8OP$	$[M-H]^-$	−1.23		0.25	0.3607
365.19674	19472.78333	64	$C_{18}H_{26}O_4$	$[M+Acetate]^-$	−0.61	[Compactin diol lactone, Di-*n*-pentyl phthalate]	−0.38	0.4029
365.19674	19472.78333	64	$C_9H_{28}N_{12}P_2$	$[M-H]^-$	1.38		0.00	1.0000
365.19674	19472.78333	64	$C_{15}H_{35}N_4PS_2$	$[M-H]^-$	−0.17		0.96	0.0377
365.19674	19472.78333	64	$C_{20}H_{30}O_6$	$[M-H]^-$	−0.61	[20-COOH-Leukotriene B4, Bis(2-butoxyethyl)phthalate, Chaparrolide]	−0.65	0.0575
365.19674	19472.78333	64	$C_{15}H_{27}N_8OP$	$[M-H]^-$	−1.45		0.96	0.0377
381.26448	80093.695	65	$C_{20}H_{34}O_3$	$[M+Acetate]^-$	−0.44	[17alpha-Methyl-5alpha-androstane-3beta,11beta,17beta-triol, 2alpha-(Hydroxymethyl)-5alpha-androstane-3beta,17beta-diol]	0.86	0.0000
381.26448	80093.695	65	$C_{22}H_{38}O_5$	$[M-H]^-$	−0.44		0.98	0.0000
381.26448	80093.695	65	$C_{17}H_{35}N_8P$	$[M-H]^-$	−1.24		0.25	0.3927
383.18395	71100.83333	66	$C_{17}H_{20}N_6O$	$[M+Acetate]^-$	0.62		0.92	0.0013
383.18395	71100.83333	66	$C_{14}H_{29}O_6P$	$[M+Acetate]^-$	−0.21		0.74	0.0364
383.18395	71100.83333	66	$C_9H_{26}N_8OP_2$	$[M+Acetate]^-$	−1.01		−0.50	0.3866
383.18395	71100.83333	66	$C_{17}H_{32}O_7$	$[M+Cl]^-$	−0.67		0.92	0.0013
383.18395	71100.83333	66	$C_{12}H_{29}N_8O_2P$	$[M+Cl]^-$	−1.47		0.10	0.8199

Table S1. *Cont.*

m/z	Intensity	ID SIP (Figure S5)	Empirical formula (all-^{12}C containing peak)	Ion form	Mass error (ppm)	KEGG compound	*r*-value	*p*-value
383.18395	71100.83333	66	$C_{19}H_{24}N_6O_3$	$[M-H]^-$	0.62		0.71	0.0472
383.18395	71100.83333	66	$C_{27}H_{28}S$	$[M-H]^-$	0.14		0.29	0.4788
383.18395	71100.83333	66	$C_{12}H_{32}N_8S_3$	$[M-H]^-$	0.05		0.10	0.8199
383.18395	71100.83333	66	$C_{16}H_{33}O_8P$	$[M-H]^-$	−0.21		0.98	0.0000
383.18395	71100.83333	66	$C_{11}H_{30}N_8O_3P_2$	$[M-H]^-$	−1.01		−0.21	0.6458
383.18395	71100.83333	66	$C_{12}H_{24}N_{12}OS$	$[M-H]^-$	−1.17		0.10	0.8199
383.18395	71100.83333	66	$C_{19}H_{34}N_2P_2S$	$[M-H]^-$	−1.49		0.71	0.0472
381.22810	224680.45	67	$C_{19}H_{30}O_4$	$[M + Acetate]^-$	−0.43	[(8)-Gingerol, Decylubiquinone, Rapanone]	0.85	0.0000
381.22810	224680.45	67	$C_{10}H_{32}N_{12}P_2$	$[M-H]^-$	1.48		0.12	0.7597
381.22810	224680.45	67	$C_{16}H_{39}N_4PS_2$	$[M-H]^-$	0		0.25	0.3607
381.22810	224680.45	67	$C_{21}H_{34}O_6$	$[M-H]^-$	−0.43	[Sarcostin]	0.96	0.0000
381.22810	224680.45	67	$C_{16}H_{31}N_8OP$	$[M-H]^-$	−1.23		0.25	0.3607
381.26448	80093.695	68	$C_{20}H_{34}O_3$	$[M + Acetate]^-$	−0.44	[17alpha-Methyl-5alpha-androstane-3beta,11beta,17beta-triol, 2alpha-(Hydroxymethyl)-5alpha-androstane-3beta,17beta-diol]	0.97	0.0000
381.26448	80093.695	68	$C_{22}H_{38}O_5$	$[M-H]^-$	−0.44		0.89	0.0000
381.26448	80093.695	68	$C_{17}H_{35}N_8P$	$[M-H]^-$	−1.24		0.60	0.0244
385.17832	35152.79833	69	$C_{17}H_{27}O_4P$	$[M + Acetate]^-$	−0.6		0.86	0.0122
385.17832	35152.79833	69	$C_{20}H_{30}O_5$	$[M + Cl]^-$	−1.06	[(5*Z*,13*E*)-11alpha-Hydroxy-9,15-dioxoprost-13-enoate, (5*Z*,13*E*)-6,9alpha-Epoxy-11alpha-hydroxy-15-oxoprosta-5,13-dienoate, (5Z,13*E*)-9alpha-Hydroxy-11,15-dioxoprosta-5,13-dienoate, 5*S*-Hydroperoxy-18R-HEPE, Chalcolactone, Prostaglandin *D*3, Prostaglandin *E3*, Resolvin *E*1]	0.99	0.0000

Table S1. *Cont.*

m/z	Intensity	ID SIP (Figure S5)	Empirical formula (all-^{12}C containing peak)	Ion form	Mass error (ppm)	KEGG compound	*r*-value	*p*-value
385.17832	35152.79833	69	$C_{22}H_{30}N_2S_2$	[M − H]⁻	1.44		0.91	0.0049
385.17832	35152.79833	69	$C_8H_{29}N_{12}P_3$	[M − H]⁻	1.28		0.00	1.0000
385.17832	35152.79833	69	$C_{22}H_{22}N_6O$	[M − H]⁻	0.23		0.91	0.0049
385.17832	35152.79833	69	$C_7H_{26}N_{14}OS_2$	[M − H]⁻	0.13		0.00	1.0000
385.17832	35152.79833	69	$C_{14}H_{36}N_4P_2S_2$	[M − H]⁻	−0.18		0.36	0.6396
385.17832	35152.79833	69	$C_{19}H_{31}O_6P$	[M − H]⁻	−0.6		0.97	0.0004
385.17832	35152.79833	69	$C_{14}H_{28}N_8OP_2$	[M − H]⁻	−1.39		0.36	0.6396
373.16557	25366.18	70	$C_{11}H_{26}N_2O_6S$	[M + Acetate]⁻	1.49		0.98	0.1231
373.16557	25366.18	70	$C_6H_{23}N_{10}OPS$	[M + Acetate]⁻	0.67		0.00	1.0000
373.16557	25366.18	70	$C_{19}H_{22}O_4$	[M + Acetate]⁻	−0.25	[2,3-Dehydro-gibberellin A9, Heliettin]	0.61	0.1985
373.16557	25366.18	70	$C_9H_{26}N_{10}O_2S$	[M + Cl]⁻	0.2		−0.05	0.9653
373.16557	25366.18	70	$C_{16}H_{36}OP_2S$	[M + Cl]⁻	−0.12		0.46	0.4405
373.16557	25366.18	70	$C_{13}H_{30}N_2O_8S$	[M − H]⁻	1.49		0.78	0.4303
373.16557	25366.18	70	$C_8H_{27}N_{10}O_3PS$	[M − H]⁻	0.67		0.00	1.0000
373.16557	25366.18	70	$C_{16}H_{31}N_4PS_2$	[M − H]⁻	0.18		0.46	0.4405
373.16557	25366.18	70	$C_{21}H_{26}O_6$	[M − H]⁻	−0.25	[Hexahydrocurcumin, Phantomolin]	−0.18	0.7050
373.16557	25366.18	70	$C_{16}H_{23}N_8OP$	[M − H]⁻	−1.07		0.46	0.4405
381.19168	45771.21333	71	$C_{18}H_{26}O_5$	[M + Acetate]⁻	−0.52	[alpha-Zearalanol, beta-Zearalanol]	−0.72	0.0271
381.19168	45771.21333	71	$C_{13}H_{23}N_8P$	[M + Acetate]⁻	−1.32		−0.99	0.0099
381.19168	45771.21333	71	$C_{10}H_{27}N_{12}P$	[M + Cl]⁻	0.92		0.84	0.3594
381.19168	45771.21333	71	$C_{16}H_{34}N_4S_2$	[M + Cl]⁻	−0.56		−0.65	0.1141
381.19168	45771.21333	71	$C_9H_{28}N_{12}OP_2$	[M − H]⁻	1.38		0.95	0.2056
381.19168	45771.21333	71	$C_{16}H_{38}N_2P_4$	[M − H]⁻	1.07		−0.65	0.1141
381.19168	45771.21333	71	$C_{15}H_{35}N_4OPS_2$	[M − H]⁻	−0.1		−0.73	0.0967

Table S1. *Cont.*

m/z	Intensity	ID SIP (Figure S5)	Empirical formula (all-^{12}C containing peak)	Ion form	Mass error (ppm)	KEGG compound	*r*-value	*p*-value
381.19168	45771.21333	71	$C_{20}H_{30}O_7$	$[M - H]^-$	−0.52	[Cinncassiol A, Cinncassiol C3]	−0.56	0.0720
381.19168	45771.21333	71	$C_{15}H_{27}N_8O_2P$	$[M - H]^-$	−1.32		−0.73	0.0967
389.21790	428073.7	72	$C_6H_{28}N_{12}P_2$	[M + Acetate]$^-$	1.37		0.00	1.0000
389.21790	428073.7	72	$C_{17}H_{30}O_6$	[M + Acetate]$^-$	−0.5		0.96	0.0000
389.21790	428073.7	72	$C_{12}H_{27}N_8OP$	[M + Acetate]$^-$	−1.28		−0.24	0.5613
389.21790	428073.7	72	$C_9H_{31}N_{12}OP$	$[M + Cl]^-$	0.92		−0.98	0.0038
389.21790	428073.7	72	$C_8H_{32}N_{12}O_2P_2$	$[M - H]^-$	1.37		−0.98	0.0215
389.21790	428073.7	72	$C_{19}H_{34}O_8$	$[M - H]^-$	−0.5	[Rehmaionoside A, Rehmaionoside B]	0.62	0.0433
389.21790	428073.7	72	$C_{14}H_{31}N_8O_3P$	$[M - H]^-$	−1.28		0.68	0.0313
397.13244	18064.2318	73	$C_{19}H_{19}N_2O_2P$	[M + Acetate]$^-$	0.43		−0.92	0.0087
397.13244	18064.2318	73	$C_9H_{18}N_6O_8$	[M + Acetate]$^-$	−0.07		−0.62	0.1910
397.13244	18064.2318	73	$C_{17}H_{22}O_5S$	[M + Acetate]$^-$	−0.53		−0.94	0.0060
397.13244	18064.2318	73	$C_4H_{15}N_{14}O_3P$	[M + Acetate]$^-$	−0.84		0.83	0.3814
397.13244	18064.2318	73	$C_{11}H_{25}N_4O_2P_3$	[M + Acetate]$^-$	−1.14		−0.90	0.0151
397.13244	18064.2318	73	$C_{12}H_{19}N_8PS$	[M + Acetate]$^-$	−1.3		−0.93	0.0064
397.13244	18064.2318	73	$C_8H_{29}N_8O_2P_3$	$[M + Cl]^-$	1.01		−0.22	0.6695
397.13244	18064.2318	73	$C_9H_{23}N_{12}PS$	$[M + Cl]^-$	0.86		−0.62	0.1910
397.13244	18064.2318	73	$C_{22}H_{22}N_2O_3$	$[M + Cl]^-$	−0.01		−0.90	0.0131
397.13244	18064.2318	73	$C_7H_{26}N_{10}O_3S_2$	$[M + Cl]^-$	−0.1		0.34	0.5037
397.13244	18064.2318	73	$C_{15}H_{30}N_4S_3$	$[M + Cl]^-$	−0.56		−0.95	0.0041
397.13244	18064.2318	73	$C_7H_{18}N_{14}O_4$	$[M + Cl]^-$	−1.28		0.34	0.5037
397.13244	18064.2318	73	$C_8H_{24}N_{12}OP_2S$	$[M - H]^-$	1.3		−0.22	0.6695
397.13244	18064.2318	73	$C_{15}H_{34}N_2P_4S$	$[M - H]^-$	0.99		−0.95	0.0041
397.13244	18064.2318	73	$C_{21}H_{23}N_2O_4P$	$[M - H]^-$	0.43		−0.91	0.0117

Table S1. *Cont.*

m/z	Intensity	ID SIP (Figure S5)	Empirical formula (all-^{12}C containing peak)	Ion form	Mass error (ppm)	KEGG compound	*r*-value	*p*-value
397.13244	18064.2318	73	$C_{11}H_{22}N_6O_{10}$	[M − H]$^-$	−0.07		−0.90	0.0151
397.13244	18064.2318	73	$C_{14}H_{31}N_4OPS_3$	[M − H]$^-$	−0.12		−0.95	0.0037
397.13244	18064.2318	73	$C_{19}H_{26}O_7S$	[M − H]$^-$	−0.53		−0.92	0.0087
397.13244	18064.2318	73	$C_6H_{19}N_{14}O_5P$	[M − H]$^-$	−0.84		0.59	0.2973
397.13244	18064.2318	73	$C_{13}H_{29}N_4O_4P_3$	[M − H]$^-$	−1.14		−0.95	0.0041
397.13244	18064.2318	73	$C_{14}H_{23}N_8O_2PS$	[M − H]$^-$	−1.3		−0.95	0.0037
389.24620	25568.11833	74	$C_{18}H_{35}O_3P$	[M + Acetate]$^-$	−0.09		0.46	0.2529
389.24620	25568.11833	74	$C_{21}H_{38}O_4$	[M + Cl]$^-$	−0.54	['Methyl acetyl ricinoleate']	0.97	0.0001
389.24620	25568.11833	74	$C_{23}H_{30}N_6$	[M − H]$^-$	0.73		0.75	0.0329
389.24620	25568.11833	74	$C_{20}H_{39}O_5P$	[M − H]$^-$	−0.09		0.98	0.0000
389.24620	25568.11833	74	$C_{15}H_{36}N_8P_2$	[M − H]$^-$	−0.88		−0.53	0.1724
383.20734	57391.08667	75	$C_{18}H_{28}O_5$	[M + Acetate]$^-$	−0.49		0.36	0.2746
383.20734	57391.08667	75	$C_{13}H_{25}N_8P$	[M + Acetate]$^-$	−1.29		0.20	0.7111
383.20734	57391.08667	75	$C_{10}H_{29}N_{12}P$	[M + Cl]$^-$	0.95		0.28	0.8206
383.20734	57391.08667	75	$C_{16}H_{36}N_4S_2$	[M + Cl]$^-$	−0.53		0.71	0.0312
383.20734	57391.08667	75	$C_9H_{30}N_{12}OP_2$	[M − H]$^-$	1.4		0.00	1.0000
383.20734	57391.08667	75	$C_{16}H_{40}N_2P_4$	[M − H]$^-$	1.09		0.71	0.0312
383.20734	57391.08667	75	$C_{15}H_{37}N_4OPS_2$	[M − H]$^-$	−0.07		0.58	0.1278
383.20734	57391.08667	75	$C_{20}H_{32}O_7$	[M − H]$^-$	−0.49		−0.11	0.7265
383.20734	57391.08667	75	$C_{15}H_{29}N_8O_2P$	[M − H]$^-$	−1.29		0.58	0.1278
393.18621	106041.4783	76	$C_{26}H_{22}$	[M + Acetate]$^-$	0.52		−0.25	0.4069
393.18621	106041.4783	76	$C_{11}H_{26}N_8S_2$	[M + Acetate]$^-$	0.43		−0.37	0.3677
393.18621	106041.4783	76	$C_{11}H_{18}N_{12}O$	[M + Acetate]$^-$	−0.75		−0.37	0.3677
393.18621	106041.4783	76	$C_{18}H_{28}N_2P_2$	[M + Acetate]$^-$	−1.06		0.95	0.0000
393.18621	106041.4783	76	$C_{15}H_{32}N_6P_2$	[M + Cl]$^-$	1.11		0.81	0.0014

Table S1. *Cont.*

m/z	Intensity	ID SIP (Figure S5)	Empirical formula (all-^{12}C containing peak)	Ion form	Mass error (ppm)	KEGG compound	*r*-value	*p*-value
393.18621	106041.4783	76	$C_5H_{26}N_{14}O_5S$	[M − H]$^−$	0.9		0.00	1.0000
393.18621	106041.4783	76	$C_{28}H_{26}O_2$	[M − H]$^−$	0.52		−0.35	0.2432
393.18621	106041.4783	76	$C_{13}H_{30}N_8O_2S_2$	[M − H]$^−$	0.43		0.23	0.5298
393.18621	106041.4783	76	$C_{13}H_{22}N_{12}O_3$	[M − H]$^−$	−0.75		0.23	0.5298
393.18621	106041.4783	76	$C_{20}H_{32}N_2O_2P_2$	[M − H]$^−$	−1.06		0.66	0.0141
393.18621	106041.4783	76	$C_{21}H_{26}N_6S$	[M − H]$^−$	−1.22		0.46	0.1177
399.19706	20047.98225	77	$C_{25}H_{24}O$	[M + Acetate]$^−$	1.23		−0.88	0.0009
399.19706	20047.98225	77	$C_{10}H_{28}N_8OS_2$	[M + Acetate]$^−$	1.14		0.11	0.8071
399.19706	20047.98225	77	$C_{10}H_{20}N_{12}O_2$	[M + Acetate]$^−$	−0.03		0.11	0.8071
399.19706	20047.98225	77	$C_{17}H_{30}N_2OP_2$	[M + Acetate]$^−$	−0.33		−0.09	0.8120
399.19706	20047.98225	77	$C_{15}H_{33}O_4PS$	[M + Acetate]$^−$	−1.29		0.50	0.1394
399.19706	20047.98225	77	$C_{20}H_{33}N_2O_2P$	[M + Cl]$^−$	−0.77		−0.66	0.0365
399.19706	20047.98225	77	$C_{27}H_{28}O_3$	[M − H]$^−$	1.23		−0.89	0.0007
399.19706	20047.98225	77	$C_{12}H_{32}N_8O_3S_2$	[M − H]$^−$	1.14		0.81	0.0082
399.19706	20047.98225	77	$C_{20}H_{36}N_2S_3$	[M − H]$^−$	0.68		−0.66	0.0365
399.19706	20047.98225	77	$C_{12}H_{24}N_{12}O_4$	[M − H]$^−$	−0.03		0.81	0.0082
399.19706	20047.98225	77	$C_{19}H_{34}N_2O_3P_2$	[M − H]$^−$	−0.33		−0.53	0.1130
399.19706	20047.98225	77	$C_{20}H_{28}N_6OS$	[M − H]$^−$	−0.49		−0.66	0.0365
399.19706	20047.98225	77	$C_{17}H_{37}O_6PS$	[M − H]$^−$	−1.29		−0.09	0.8120
397.22321	234445.4	78	$C_{19}H_{30}O_5$	[M + Acetate]$^−$	0.08	['Piperonyl butoxide', 'Shiromodiol diacetate']	1.00	0.0000
397.22321	234445.4	78	$C_{14}H_{27}N_8P$	[M + Acetate]$^−$	−0.69		−0.16	0.6670

Table S1. *Cont.*

m/z	Intensity	ID SIP (Figure S5)	Empirical formula (all-^{12}C containing peak)	Ion form	Mass error (ppm)	KEGG compound	*r*-value	*p*-value
397.22321	234445.4	78	$C_{11}H_{31}N_{12}P$	$[M + Cl]^-$	1.47		−0.87	0.0105
397.22321	234445.4	78	$C_{17}H_{38}N_4S_2$	$[M + Cl]^-$	0.05		0.84	0.0003
397.22321	234445.4	78	$C_{17}H_{30}N_8O$	$[M + Cl]^-$	−1.13		0.84	0.0003
397.22321	234445.4	78	$C_{16}H_{39}N_4OPS_2$	$[M - H]^-$	0.49		0.60	0.0386
397.22321	234445.4	78	$C_{21}H_{34}O_7$	$[M - H]^-$	0.08		0.87	0.0000
397.22321	234445.4	78	$C_{16}H_{31}N_8O_2P$	$[M - H]^-$	−0.69		0.60	0.0386
395.31626	35088.42333	79	$C_{22}H_{40}O_2$	$[M + Acetate]^-$	−1.07	[13,16-Docosadienoic acid]	0.99	0.0000
395.31626	35088.42333	79	$C_{19}H_{44}N_4O_2$	$[M + Cl]^-$	1.09		0.54	0.0713
395.31626	35088.42333	79	$C_{24}H_{44}O_4$	$[M - H]^-$	−1.07		0.85	0.0004
401.21809	75247.478	80	$C_{18}H_{30}O_6$	$[M + Acetate]^-$	−0.01		0.57	0.1843
401.21809	75247.478	80	$C_{13}H_{27}N_8OP$	$[M + Acetate]^-$	−0.77		0.21	0.6583
401.21809	75247.478	80	$C_{10}H_{31}N_{12}OP$	$[M + Cl]^-$	1.36		−0.87	0.1338
401.21809	75247.478	80	$C_{17}H_{41}N_2P_3$	$[M + Cl]^-$	1.06		0.82	0.0245
401.21809	75247.478	80	$C_{16}H_{38}N_4OS_2$	$[M + Cl]^-$	−0.04		0.91	0.0049
401.21809	75247.478	80	$C_{16}H_{30}N_8O_2$	$[M + Cl]^-$	−1.21		0.91	0.0049
401.21809	75247.478	80	$C_{20}H_{34}O_8$	$[M - H]^-$	−0.01		0.13	0.7761
401.21809	75247.478	80	$C_{15}H_{31}N_8O_3P$	$[M - H]^-$	−0.77		0.75	0.0518
401.21809	75247.478	80	$C_{23}H_{35}N_2PS$	$[M - H]^-$	−1.23		−0.18	0.7015
403.23348	185445.3333	81	$C_7H_{30}N_{12}P_2$	$[M + Acetate]^-$	1.15		0.00	1.0000
403.23348	185445.3333	81	$C_{18}H_{32}O_6$	$[M + Acetate]^-$	−0.65		0.68	0.0654
403.23348	185445.3333	81	$C_{13}H_{29}N_8OP$	$[M + Acetate]^-$	−1.41		−0.88	0.0205
403.23348	185445.3333	81	$C_{15}H_{36}N_4O_6$	$[M + Cl]^-$	1.47		−0.08	0.8504
403.23348	185445.3333	81	$C_{10}H_{33}N_{12}OP$	$[M + Cl]^-$	0.71		0.32	0.7915
403.23348	185445.3333	81	$C_9H_{34}N_{12}O_2P_2$	$[M - H]^-$	1.15		0.00	1.0000

Table S1. *Cont.*

m/z	Intensity	ID SIP (Figure S5)	Empirical formula (all-^{12}C containing peak)	Ion form	Mass error (ppm)	KEGG compound	*r*-value	*p*-value
403.23348	185445.3333	81	$C_{20}H_{36}O_8$	$[M - H]^-$	−0.65		0.78	0.0229
403.23348	185445.3333	81	$C_{15}H_{33}N_8O_3P$	$[M - H]^-$	−1.41		−0.08	0.8504
409.29594	15403.1562	82	$C_{22}H_{38}O_3$	$[M + Acetate]^-$	−0.02		0.99	0.0000
409.29594	15403.1562	82	$C_{24}H_{42}O_5$	$[M - H]^-$	−0.02		0.91	0.0000
409.29594	15403.1562	82	$C_{19}H_{39}N_8P$	$[M - H]^-$	−0.77		0.55	0.0506
439.21020	8604.357667	83	$C_{20}H_{24}N_6O_2$	$[M + Acetate]^-$	0.62		0.23	0.5201
439.21020	8604.357667	83	$C_{17}H_{33}O_7P$	$[M + Acetate]^-$	−0.11		0.56	0.0902
439.21020	8604.357667	83	$C_{12}H_{30}N_8O_2P_2$	$[M + Acetate]^-$	−0.8		0.81	0.0047
439.21020	8604.357667	83	$C_{13}H_{24}N_{12}S$	$[M + Acetate]^-$	−0.94		0.93	0.0001
439.21020	8604.357667	83	$C_9H_{34}N_{12}O_2P_2$	[M + Cl]−	1.15		0.02	0.9693
439.21020	8604.357667	83	$C_{20}H_{36}O_8$	$[M + Cl]^-$	−0.5		0.23	0.5201
439.21020	8604.357667	83	$C_{15}H_{33}N_8O_3P$	$[M + Cl]^-$	−1.2		0.86	0.0015
439.21020	8604.357667	83	$C_{16}H_{39}N_6P_3S$	$[M - H]^-$	1.13		0.72	0.0198
439.21020	8604.357667	83	$C_{22}H_{28}N_6O_4$	$[M - H]^-$	0.62		0.11	0.7518
439.21020	8604.357667	83	$C_{30}H_{32}OS$	$[M - H]^-$	0.2		−0.05	0.8932
439.21020	8604.357667	83	$C_{15}H_{36}N_8OS_3$	$[M - H]^-$	0.12		0.86	0.0015
439.21020	8604.357667	83	$C_{19}H_{37}O_9P$	$[M - H]^-$	−0.11		0.32	0.3723
439.21020	8604.357667	83	$C_{14}H_{34}N_8O_4P_2$	$[M - H]^-$	−0.8		0.94	0.0000
439.21020	8604.357667	83	$C_{15}H_{28}N_{12}O_2S$	$[M - H]^-$	−0.94		0.86	0.0015
439.21020	8604.357667	83	$C_{22}H_{38}N_2OP_2S$	$[M - H]^-$	−1.22		0.11	0.7518
445.28076	122661.2567	84	$C_{21}H_{38}O_6$	$[M + Acetate]^-$	0.15	[6-Deoxyerythronolide B]	−0.25	0.5154
445.28076	122661.2567	84	$C_{16}H_{35}N_8OP$	$[M + Acetate]^-$	−0.54		0.15	0.7056
445.28076	122661.2567	84	$C_{13}H_{39}N_{12}OP$	$[M + Cl]^-$	1.39		0.78	0.0124

Table S1. *Cont.*

m/z	Intensity	ID SIP (Figure S5)	Empirical formula (all-^{12}C containing peak)	Ion form	Mass error (ppm)	KEGG compound	*r*-value	*p*-value
445.28076	122661.2567	84	$C_{19}H_{38}N_8O_2$	$[M + Cl]^-$	−0.93		−0.16	0.6724
445.28076	122661.2567	84	$C_{23}H_{42}O_8$	$[M - H]^-$	0.15		−0.30	0.4397
445.28076	122661.2567	84	$C_{18}H_{39}N_8O_3P$	$[M - H]^-$	−0.54		−0.09	0.8106
445.28076	122661.2567	84	$C_{26}H_{43}N_2P_S$	$[M - H]^-$	−0.95		−0.33	0.3885
441.28557	12578.83083	85	$C_{22}H_{38}O_5$	$[M + Acetate]^-$	−0.47		0.24	0.6961
441.28557	12578.83083	85	$C_{17}H_{35}N_8P$	$[M + Acetate]^-$	−1.16		0.71	0.1760
441.28557	12578.83083	85	$C_{19}H_{42}N_4O_5$	$[M + Cl]^-$	1.47		0.46	0.4342
441.28557	12578.83083	85	$C_{14}H_{39}N_{12}P$	$[M + Cl]^-$	0.78		0.99	0.0006
441.28557	12578.83083	85	$C_{20}H_{46}N_{4S2}$	$[M + Cl]^-$	−0.5		0.37	0.5396
441.28557	12578.83083	85	$C_{13}H_{40}N_{12}OP_2$	$[M - H]^-$	1.17		0.93	0.0236
441.28557	12578.83083	85	$C_{24}H_{42}O_7$	$[M - H]^-$	−0.47		0.16	0.8007
441.28557	12578.83083	85	$C_{19}H_{39}N_8O_2P$	$[M - H]^-$	−1.16		0.46	0.4342
441.24929	99110.558	86	$C_{10}H_{32}N_{12}P_2$	$[M + Acetate]^-$	1.41		−0.96	0.1717
441.24929	99110.558	86	$C_{16}H_{39}N_4P_{S2}$	$[M + Acetate]^-$	0.13		−0.47	0.2049
441.24929	99110.558	86	$C_{21}H_{34}O_6$	$[M + Acetate]^-$	−0.23	[Sarcostin]	0.99	0.0000
441.24929	99110.558	86	$C_{16}H_{31}N_8OP$	$[M + Acetate]^-$	−0.93		−0.47	0.2049
441.24929	99110.558	86	$C_{13}H_{35}N_{12}OP$	$[M + Cl]^-$	1.01		−1.00	0.0000
441.24929	99110.558	86	$C_{20}H_{45}N_2P_3$	$[M + Cl]^-$	0.74		0.89	0.0001
441.24929	99110.558	86	$C_{19}H_{42}N_4O_{S2}$	$[M + Cl]^-$	−0.26		0.72	0.0078
441.24929	99110.558	86	$C_{19}H_{34}N_8O_2$	$[M + Cl]^-$	−1.32		0.72	0.0078
441.24929	99110.558	86	$C_{12}H_{36}N_{12}O_2P_2$	$[M - H]^-$	1.41		−0.97	0.0070
441.24929	99110.558	86	$C_{19}H_{46}N_2OP_4$	$[M - H]^-$	1.14		0.72	0.0078
441.24929	99110.558	86	$C_{18}H_{43}N_4O_2P_{S2}$	$[M - H]^-$	0.13		0.43	0.1924

Table S1. *Cont.*

m/z	Intensity	ID SIP (Figure S5)	Empirical formula (all-^{12}C containing peak)	Ion form	Mass error (ppm)	KEGG compound	*r*-value	*p*-value
441.24929	99110.558	86	$C_{23}H_{38}O_8$	$[M-H]^-$	−0.23		0.90	0.0001
441.24929	99110.558	86	$C_{18}H_{35}N_8O_3P$	$[M-H]^-$	−0.93		0.43	0.1924
441.24929	99110.558	86	$C_{26}H_{39}N_2PS$	$[M-H]^-$	−1.34		0.27	0.4043
437.29118	21714.87667	87	$C_{23}H_{38}O_4$	$[M + Acetate]^-$	0.72	[1-Arachidonoylglycerol, 2-Arachidonoylglycerol]	0.87	0.0053
437.29118	21714.87667	87	$C_{16}H_{38}N_6O_2S$	$[M + Acetate]^-$	−0.85		−0.66	0.1051
437.29118	21714.87667	87	$C_{21}H_{38}N_8$	$[M + Cl]^-$	−0.37		0.75	0.0316
437.29118	21714.87667	87	$C_{20}H_{47}N_4PS_2$	$[M-H]^-$	1.09		0.51	0.1990
437.29118	21714.87667	87	$C_{25}H_{42}O_6$	$[M-H]^-$	0.72		0.45	0.2628
437.29118	21714.87667	87	$C_{20}H_{39}N_8OP$	$[M-H]^-$	0.03		0.51	0.1990
437.29118	21714.87667	87	$C_{18}H_{42}N_6O_4S$	$[M-H]^-$	−0.85		0.04	0.9240
437.29118	21714.87667	87	$C_{26}H_{46}OS_2$	$[M-H]^-$	−1.26		0.26	0.5401
441.32194	13986.06367	88	$C_{23}H_{42}O_4$	$[M + Acetate]^-$	−0.51		0.95	0.0009
441.32194	13986.06367	88	$C_{20}H_{46}N_4O_4$	$[M + Cl]^-$	1.43		0.76	0.0470
441.32194	13986.06367	88	$C_{14}H_{44}N_{12}P_2$	$[M-H]^-$	1.14		0.00	1.0000
441.32194	13986.06367	88	$C_{25}H_{46}O_6$	$[M-H]^-$	−0.51		0.89	0.0079
441.32194	13986.06367	88	$C_{20}H_{43}N_8OP$	$[M-H]^-$	−1.2		0.76	0.0470
449.23909	11970.7975	89	$C_8H_{32}N_{12}O_2P_2$	$[M + Acetate]^-$	1.32		−0.85	0.3511
449.23909	11970.7975	89	$C_{19}H_{34}O_8$	$[M + Acetate]^-$	−0.3	[Rehmaionoside A, Rehmaionoside B]	−0.22	0.6723
449.23909	11970.7975	89	$C_{14}H_{31}N_8O_3P$	$[M + Acetate]^-$	−0.98		0.67	0.1422
449.23909	11970.7975	89	$C_{22}H_{35}N_2PS$	$[M + Acetate]^-$	−1.38		−0.35	0.5000
449.23909	11970.7975	89	$C_{11}H_{35}N_{12}O_3P$	$[M + Cl]^-$	0.93		0.33	0.5192
449.23909	11970.7975	89	$C_{19}H_{39}N_6PS$	$[M + Cl]^-$	0.52		−0.22	0.6723
449.23909	11970.7975	89	$C_{17}H_{34}N_8O_4$	$[M + Cl]^-$	−1.37		−0.03	0.9520

Table S1. *Cont.*

m/z	Intensity	ID SIP (Figure S5)	Empirical formula (all-^{12}C containing peak)	Ion form	Mass error (ppm)	KEGG compound	*r*-value	*p*-value
449.23909	11970.7975	89	$C_{10}H_{36}N_{12}O_4P_2$	$[M-H]^-$	1.32		−0.21	0.7355
449.23909	11970.7975	89	$C_{18}H_{40}N_6OP_2S$	$[M-H]^-$	0.91		−0.14	0.7843
449.23909	11970.7975	89	$C_{19}H_{26}N_{14}$	$[M-H]^-$	−0.27		−0.22	0.6723
449.23909	11970.7975	89	$C_{21}H_{38}O_{10}$	$[M-H]^-$	−0.3		−0.32	0.5401
449.23909	11970.7975	89	$C_{16}H_{35}N_8O_5P$	$[M-H]^-$	−0.98		0.14	0.7940
449.23909	11970.7975	89	$C_{24}H_{39}N_2O_2PS$	$[M-H]^-$	−1.38		−0.39	0.4469
457.35308	13969.922	90	$C_{24}H_{46}O_4$	$[M+Acetate]^-$	−0.84	[Lauroyl peroxide]	0.77	0.0250
457.35308	13969.922	90	$C_{26}H_{50}O_6$	$[M-H]^-$	−0.84		0.73	0.0391
447.21516	25773.18367	91	$C_{22}H_{32}N_2S_2$	$[M+Acetate]^-$	1.37		0.90	0.0003
447.21516	25773.18367	91	$C_8H_{31}N_{12}P_3$	$[M+Acetate]^-$	1.24		0.00	1.0000
447.21516	25773.18367	91	$C_{22}H_{24}N_6O$	$[M+Acetate]^-$	0.33		0.90	0.0003
447.21516	25773.18367	91	$C_7H_{28}N_{14}OS_2$	$[M+Acetate]^-$	0.25		0.00	1.0000
447.21516	25773.18367	91	$C_{19}H_{33}O_6P$	$[M+Acetate]^-$	−0.38		0.82	0.0038
447.21516	25773.18367	91	$C_{14}H_{30}N_8OP_2$	$[M+Acetate]^-$	−1.07		−0.76	0.0291
447.21516	25773.18367	91	$C_{11}H_{34}N_{12}OP_2$	$[M+Cl]^-$	0.85		−0.97	0.0051
447.21516	25773.18367	91	$C_{18}H_{44}N_2P_4$	$[M+Cl]^-$	0.58		0.57	0.0838
447.21516	25773.18367	91	$C_{17}H_{41}N_4OPS_2$	$[M+Cl]^-$	−0.41		0.27	0.4583
447.21516	25773.18367	91	$C_{22}H_{36}O_7$	$[M+Cl]^-$	−0.78	[Grayanotoxin I]	0.90	0.0003
447.21516	25773.18367	91	$C_{17}H_{33}N_8O_2P$	$[M+Cl]^-$	−1.46		0.27	0.4583
447.21516	25773.18367	91	$C_{24}H_{36}N_2O_2S_2$	$[M-H]^-$	1.37		0.65	0.0436
447.21516	25773.18367	91	$C_{10}H_{35}N_{12}O_2P_3$	$[M-H]^-$	1.24		−0.99	0.0058
447.21516	25773.18367	91	$C_{24}H_{28}N_6O_3$	$[M-H]^-$	0.33		0.65	0.0436
447.21516	25773.18367	91	$C_9H_{32}N_{14}O_3S_2$	$[M-H]^-$	0.25		−0.96	0.1858

Table S1. *Cont.*

m/z	Intensity	ID SIP (Figure S5)	Empirical formula (all-^{12}C containing peak)	Ion form	Mass error (ppm)	KEGG compound	*r*-value	*p*-value
447.21516	25773.18367	91	$C_{32}H_{32}S$	$[M - H]^-$	−0.08		0.07	0.8582
447.21516	25773.18367	91	$C_{17}H_{36}N_8S_3$	$[M - H]^-$	−0.16		0.27	0.4583
447.21516	25773.18367	91	$C_{21}H_{37}O_8P$	$[M - H]^-$	−0.38		0.98	0.0000
447.21516	25773.18367	91	$C_{16}H_{34}N_8O_3P_2$	$[M - H]^-$	−1.07		−0.05	0.8820
447.21516	25773.18367	91	$C_{17}H_{28}N_{12}OS$	$[M - H]^-$	−1.2		0.27	0.4583
447.21516	25773.18367	91	$C_{24}H_{38}N_2P_2S$	$[M - H]^-$	−1.47		0.65	0.0436
451.21017	9646.401333	92	$C_7H_{31}N_{12}OP_3$	$[M + Acetate]^-$	1.44		0.00	1.0000
451.21017	9646.401333	92	$C_{21}H_{24}N_6O_2$	$[M + Acetate]^-$	0.54		0.76	0.0297
451.21017	9646.401333	92	$C_6H_{28}N_{14}O_2S_2$	$[M + Acetate]^-$	0.46		0.00	1.0000
451.21017	9646.401333	92	$C_{18}H_{33}O_7P$	$[M + Acetate]^-$	−0.17		−0.24	0.5708
451.21017	9646.401333	92	$C_{13}H_{30}N_8O_2P_2$	$[M + Acetate]^-$	−0.85		−0.90	0.0150
451.21017	9646.401333	92	$C_{14}H_{24}N_{12}S$	$[M + Acetate]^-$	−0.98		−0.90	0.0061
451.21017	9646.401333	92	$C_{10}H_{34}N_{12}O_2P_2$	$[M + Cl]^-$	1.05		0.08	0.9473
451.21017	9646.401333	92	$C_{21}H_{36}O_8$	$[M + Cl]^-$	−0.56		0.76	0.0297
451.21017	9646.401333	92	$C_{16}H_{33}N_8O_3P$	$[M + Cl]^-$	−1.23		−0.78	0.0228
451.21017	9646.401333	92	$C_9H_{35}N_{12}O_3P_3$	$[M - H]^-$	1.44		0.00	1.0000
451.21017	9646.401333	92	$C_{17}H_{39}N_6P_3S$	$[M - H]^-$	1.03		−0.57	0.1415
451.21017	9646.401333	92	$C_{23}H_{28}N_6O_4$	$[M - H]^-$	0.54		0.88	0.0040
451.21017	9646.401333	92	$C_8H_{32}N_{14}O_4S_2$	$[M - H]^-$	0.46		0.00	1.0000
451.21017	9646.401333	92	$C_{31}H_{32}OS$	$[M - H]^-$	0.13		0.73	0.0398
451.21017	9646.401333	92	$C_{16}H_{36}N_8OS_3$	$[M - H]^-$	0.05		−0.78	0.0228
451.21017	9646.401333	92	$C_{20}H_{37}O_9P$	$[M - H]^-$	−0.17		0.54	0.1690
451.21017	9646.401333	92	$C_{15}H_{34}N_8O_4P_2$	$[M - H]^-$	−0.85		−0.89	0.0034

Table S1. *Cont.*

m/z	Intensity	ID SIP (Figure S5)	Empirical formula (all-^{12}C containing peak)	Ion form	Mass error (ppm)	KEGG compound	*r*-value	*p*-value
451.21017	9646.401333	92	$C_{16}H_{28}N_{12}O_2S$	$[M - H]^-$	−0.98		−0.78	0.0228
451.21017	9646.401333	92	$C_{23}H_{38}N_2OP_2S$	$[M - H]^-$	−1.25		0.88	0.0040
453.24925	49409.43667	93	$C_{11}H_{32}N_{12}P_2$	$[M + Acetate]^-$	1.28		−0.56	0.4411
453.24925	49409.43667	93	$C_{17}H_{39}N_4PS_2$	$[M + Acetate]^-$	0.04		−0.40	0.2529
453.24925	49409.43667	93	$C_{22}H_{34}O_6$	$[M + Acetate]^-$	−0.32		0.74	0.0062
453.24925	49409.43667	93	$C_{17}H_{31}N_8OP$	$[M + Acetate]^-$	−0.99		−0.40	0.2529
453.24925	49409.43667	93	$C_{14}H_{35}N_{12}OP$	$[M + Cl]^-$	0.9		−0.63	0.1302
453.24925	49409.43667	93	$C_{21}H_{45}N_2P_3$	$[M + Cl]^-$	0.63		0.55	0.0619
453.24925	49409.43667	93	$C_{20}H_{42}N_4OS_2$	$[M + Cl]^-$	−0.35		0.34	0.2724
453.24925	49409.43667	93	$C_{20}H_{34}N_8O_2$	$[M + Cl]^-$	−1.38		0.34	0.2724
453.24925	49409.43667	93	$C_{13}H_{36}N_{12}O_2P_2$	$[M - H]^-$	1.28		−0.64	0.1737
453.24925	49409.43667	93	$C_{20}H_{46}N_2OP_4$	$[M - H]^-$	1.02		0.34	0.2724
453.24925	49409.43667	93	$C_{19}H_{43}N_4O_2PS_2$	$[M - H]^-$	0.04		0.12	0.7006
453.24925	49409.43667	93	$C_{24}H_{38}O_8$	$[M - H]^-$	−0.32	[Rhodojaponin IV]	0.96	0.0000
453.24925	49409.43667	93	$C_{19}H_{35}N_8O_3P$	$[M - H]^-$	−0.99		0.12	0.7006
453.24925	49409.43667	93	$C_{27}H_{39}N_2PS$	$[M - H]^-$	−1.39		0.82	0.0011
461.27604	11097.01975	94	$C_{21}H_{38}O_7$	$[M + Acetate]^-$	0.94	[Erythronolide B]	−0.52	0.1536
461.27604	11097.01975	94	$C_{16}H_{35}N_8O_2P$	$[M + Acetate]^-$	0.27		−0.13	0.7446
461.27604	11097.01975	94	$C_{22}H_{42}O_2S_2$	$[M + Acetate]^-$	−0.95		−0.54	0.1337
461.27604	11097.01975	94	$C_9H_{35}N_{14}PS$	$[M + Acetate]^-$	−1.21		0.52	0.2340
461.27604	11097.01975	94	$C_{19}H_{38}N_8O_3$	$[M + Cl]^-$	−0.11		−0.44	0.2364
461.27604	11097.01975	94	$C_{27}H_{42}N_2S$	$[M + Cl]^-$	−0.5		−0.58	0.0998
461.27604	11097.01975	94	$C_{23}H_{42}O_9$	$[M - H]^-$	0.94		−0.56	0.1208

Table S1. *Cont.*

m/z	Intensity	ID SIP (Figure S5)	Empirical formula (all-^{12}C containing peak)	Ion form	Mass error (ppm)	KEGG compound	*r*-value	*p*-value
461.27604	11097.01975	94	$C_{18}H_{39}N_8O_4P$	$[M - H]^-$	0.27		−0.37	0.3242
461.27604	11097.01975	94	$C_{26}H_{43}N_2OPS$	$[M - H]^-$	−0.12		−0.58	0.1024
461.27604	11097.01975	94	$C_{24}H_{46}O_4S_2$	$[M - H]^-$	−0.95		−0.57	0.1121
461.27604	11097.01975	94	$C_{11}H_{39}N_{14}O_2PS$	$[M - H]^-$	−1.21		0.95	0.0001
457.35308	13969.922	95	$C_{24}H_{46}O_4$	$[M + Acetate]^-$	−0.84	[Lauroyl peroxide]	0.77	0.0250
457.35308	13969.922	95	$C_{26}H_{50}O_6$	[M − H]−	−0.84		0.73	0.0391
457.24425	27036.51833	96	$C_{10}H_{32}N_{12}OP_2$	$[M + Acetate]^-$	1.46		0.00	1.0000
457.24425	27036.51833	96	$C_{17}H_{42}N_2P_4$	$[M + Acetate]^-$	1.19		0.29	0.4500
457.24425	27036.51833	96	$C_{16}H_{39}N_4OPS_2$	$[M + Acetate]^-$	0.23		−0.17	0.6947
457.24425	27036.51833	96	$C_{21}H_{34}O_7$	$[M + Acetate]^-$	−0.13		0.98	0.0000
457.24425	27036.51833	96	$C_{16}H_{31}N_8O_2P$	$[M + Acetate]^-$	−0.8		−0.17	0.6947
457.24425	27036.51833	96	$C_{13}H_{35}N_{12}O_2P$	$[M + Cl]^-$	1.08		−0.97	0.0071
457.24425	27036.51833	96	$C_{20}H_{45}N_2OP_3$	$[M + Cl]^-$	0.81		0.93	0.0003
457.24425	27036.51833	96	$C_{19}H_{42}N_4O_2S_2$	$[M + Cl]^-$	−0.16		0.76	0.0177
457.24425	27036.51833	96	$C_{19}H_{34}N_8O_3$	$[M + Cl]^-$	−1.18		0.76	0.0177
457.24425	27036.51833	96	$C_{12}H_{36}N_{12}O_3P_2$	$[M - H]^-$	1.46		−0.92	0.0806
457.24425	27036.51833	96	$C_{19}H_{46}N_2O_2P_4$	$[M - H]^-$	1.19		0.76	0.0177
457.24425	27036.51833	96	$C_{20}H_{40}N_6P_2S$	$[M - H]^-$	1.06		0.93	0.0003
457.24425	27036.51833	96	$C_{18}H_{43}N_4O_3PS_2$	$[M - H]^-$	0.23		0.53	0.1419
457.24425	27036.51833	96	$C_{23}H_{38}O_9$	$[M - H]^-$	−0.13		0.62	0.0723
457.24425	27036.51833	96	$C_{18}H_{35}N_8O_4P$	$[M - H]^-$	−0.8		0.53	0.1419
457.24425	27036.51833	96	$C_{26}H_{39}N_2OPS$	$[M - H]^-$	−1.2		−0.08	0.8342
455.26517	105234.545	97	$C_{17}H_{41}N_4PS_2$	$[M + Acetate]^-$	0.63		−0.27	0.5159

Table S1. *Cont.*

m/z	Intensity	ID SIP (Figure S5)	Empirical formula (all-^{12}C containing peak)	Ion form	Mass error (ppm)	KEGG compound	*r*-value	*p*-value
455.26517	105234.545	97	$C_{22}H_{36}O_6$	[M + Acetate]$^−$	0.28		0.66	0.0771
455.26517	105234.545	97	$C_{17}H_{33}N_8OP$	[M + Acetate]$^−$	−0.39		−0.27	0.5159
455.26517	105234.545	97	$C_{15}H_{36}N_6O_4S$	[M + Acetate]$^−$	−1.23		−0.51	0.2982
455.26517	105234.545	97	$C_{14}H_{37}N_{12}OP$	[M + Cl]$^−$	1.49		−0.73	0.1577
455.26517	105234.545	97	$C_{21}H_{47}N_2P_3$	[M + Cl]$^−$	1.22		0.42	0.3010
455.26517	105234.545	97	$C_{20}H_{44}N_4OS_2$	[M + Cl]$^−$	0.25		0.20	0.6387
455.26517	105234.545	97	$C_{20}H_{36}N_8O_2$	[M + Cl]$^−$	−0.78		0.20	0.6387
455.26517	105234.545	97	$C_{19}H_{45}N_4O_2PS_2$	[M − H]$^−$	0.63		0.01	0.9768
455.26517	105234.545	97	$C_{24}H_{40}O_8$	[M − H]$^−$	0.28		0.96	0.0002
455.26517	105234.545	97	$C_{19}H_{37}N_8O_3P$	[M − H]$^−$	−0.39		0.01	0.9768
455.26517	105234.545	97	$C_{27}H_{41}N_2PS$	[M − H]$^−$	−0.79		0.84	0.0084
455.26517	105234.545	97	$C_{17}H_{40}N_6O_6S$	[M − H]$^−$	−1.23		−0.27	0.5159
463.15180	14650.97	98	$C_8H_{24}N_{10}O_5S_2$	[M + Acetate]$^−$	1.48		0.00	1.0000
463.15180	14650.97	98	$C_{15}H_{34}O_4P_2S_2$	[M + Acetate]$^−$	1.22		−0.30	0.4727
463.15180	14650.97	98	$C_{16}H_{28}N_4O_2S_3$	[M + Acetate]$^−$	1.09		0.23	0.5589
463.15180	14650.97	98	$C_{18}H_{17}N_{10}P$	[M + Acetate]$^−$	0.9		0.74	0.0232
463.15180	14650.97	98	$C_8H_{16}N_{14}O_6$	[M + Acetate]$^−$	0.48		0.00	1.0000
463.15180	14650.97	98	$C_{15}H_{26}N_4O_5P_2$	[M + Acetate]$^−$	0.21		−0.30	0.4727
463.15180	14650.97	98	$C_{16}H_{20}N_8O_3S$	[M + Acetate]$^−$	0.08		0.23	0.5589
463.15180	14650.97	98	$C_{24}H_{24}N_2S_2$	[M + Acetate]$^−$	−0.31		0.13	0.7335
463.15180	14650.97	98	$C_9H_{28}N_{10}S_4$	[M + Acetate]$^−$	−0.39		0.00	1.0000
463.15180	14650.97	98	$C_{10}H_{23}N_{12}P_3$	[M + Acetate]$^−$	−0.44		−0.95	0.2019
463.15180	14650.97	98	$C_{13}H_{29}N_2O_8PS$	[M + Acetate]$^−$	−0.61		−0.97	0.0012

Table S1. *Cont.*

m/z	Intensity	ID SIP (Figure S5)	Empirical formula (all-^{12}C containing peak)	Ion form	Mass error (ppm)	KEGG compound	*r*-value	*p*-value
463.15180	14650.97	98	$C_8H_{26}N_{10}O_3P_2S$	$[M + Acetate]^-$	−1.27		0.00	1.0000
463.15180	14650.97	98	$C_{24}H_{16}N_6O$	$[M + Acetate]^-$	−1.32		0.13	0.7335
463.15180	14650.97	98	$C_9H_{20}N_{14}OS_2$	$[M + Acetate]^-$	−1.4		0.00	1.0000
463.15180	14650.97	98	$C_{18}H_{37}O_5PS_2$	$[M + Cl]^-$	0.85		0.74	0.0232
463.15180	14650.97	98	$C_{21}H_{20}N_{10}O$	$[M + Cl]^-$	0.53		0.82	0.0070
463.15180	14650.97	98	$C_{28}H_{30}P_2$	$[M + Cl]^-$	0.26		−0.34	0.3700
463.15180	14650.97	98	$C_{13}H_{34}N_8P_2S_2$	$[M + Cl]^-$	0.19		−0.97	0.0012
463.15180	14650.97	98	$C_{18}H_{29}N_4O_6P$	$[M + Cl]^-$	−0.16		0.74	0.0232
463.15180	14650.97	98	$C_{13}H_{26}N_{12}OP_2$	$[M + Cl]^-$	−0.82		−0.97	0.0012
463.15180	14650.97	98	$C_{16}H_{32}N_2O_9S$	$[M + Cl]^-$	−0.99		0.23	0.5589
463.15180	14650.97	98	$C_{19}H_{41}PS_4$	$[M + Cl]^-$	−1.03		0.91	0.0006
463.15180	14650.97	98	$C_{20}H_{36}N_2P_4$	$[M + Cl]^-$	−1.08		0.95	0.0001
463.15180	14650.97	98	$C_{10}H_{28}N_{10}O_7S_2$	$[M - H]^-$	1.48		−0.95	0.2019
463.15180	14650.97	98	$C_{17}H_{38}O_6P_2S_2$	$[M - H]^-$	1.22		0.49	0.1825
463.15180	14650.97	98	$C_{18}H_{32}N_4O_4S_3$	$[M - H]^-$	1.09		0.74	0.0232
463.15180	14650.97	98	$C_{20}H_{21}N_{10}O_2P$	$[M - H]^-$	0.9		0.95	0.0001
463.15180	14650.97	98	$C_{27}H_{31}OP_3$	$[M - H]^-$	0.64		−0.27	0.4907
463.15180	14650.97	98	$C_{12}H_{35}N_8OP_3S_2$	$[M - H]^-$	0.56		−0.94	0.0161
463.15180	14650.97	98	$C_{10}H_{20}N_{14}O_8$	$[M - H]^-$	0.48		−0.95	0.2019
463.15180	14650.97	98	$C_{17}H_{30}N_4O_7P_2$	$[M - H]^-$	0.21		0.49	0.1825
463.15180	14650.97	98	$C_{18}H_{24}N_8O_5S$	$[M - H]^-$	0.08		0.74	0.0232
463.15180	14650.97	98	$C_{26}H_{28}N_2O_2S_2$	$[M - H]^-$	−0.31		−0.17	0.6694

Table S1. *Cont.*

m/z	Intensity	ID SIP (Figure S5)	Empirical formula (all-^{12}C containing peak)	Ion form	Mass error (ppm)	KEGG compound	*r*-value	*p*-value
463.15180	14650.97	98	$C_{11}H_{32}N_{10}O_2S_4$	$[M-H]^-$	−0.39		−0.85	0.1493
463.15180	14650.97	98	$C_{12}H_{27}N_{12}O_2P_3$	$[M-H]^-$	−0.44		−0.94	0.0161
463.15180	14650.97	98	$C_{15}H_{33}N_2O_{10}PS$	$[M-H]^-$	−0.61		−0.30	0.4727
463.15180	14650.97	98	$C_{19}H_{37}N_2OP_5$	$[M-H]^-$	−0.71		0.91	0.0006
463.15180	14650.97	98	$C_{10}H_{30}N_{10}O_5P_2S$	$[M-H]^-$	−1.27		−0.95	0.2019
463.15180	14650.97	98	$C_{26}H_{20}N_6O_3$	$[M-H]^-$	−1.32		−0.17	0.6694
463.15180	14650.97	98	$C_{11}H_{24}N_{14}O_3S_2$	$[M-H]^-$	−1.4		−0.85	0.1493
457.25979	15150.5488	99	$C_{12}H_{35}N_{10}OPS$	$[M + Acetate]^-$	1.25		0.81	0.4036
457.25979	15150.5488	99	$C_{25}H_{34}O_4$	$[M + Acetate]^-$	0.5	[17-Propylestra-1,3,5(10)-triene-3,17beta-diol diacetate, Pregna-5,16,20-triene-3beta,20-diol diacetate]	0.37	0.1979
457.25979	15150.5488	99	$C_{10}H_{30}N_{12}O_5$	$[M + Acetate]^-$	−0.6		0.93	0.2469
457.25979	15150.5488	99	$C_{17}H_{40}N_2O_4P_2$	$[M + Acetate]^-$	−0.87		−0.59	0.2219
457.25979	15150.5488	99	$C_{18}H_{34}N_6O_2S$	$[M + Acetate]^-$	−1.01		−0.47	0.2865
457.25979	15150.5488	99	$C_{15}H_{38}N_{10}O_2S$	$[M + Cl]_-$	0.87		−0.12	0.8795
457.25979	15150.5488	99	$C_{22}H_{48}OP_2S$	$[M + Cl]_-$	0.6		0.29	0.3794
457.25979	15150.5488	99	$C_{23}H_{34}N_8$	$[M + Cl]_-$	−0.55		0.37	0.2388
457.25979	15150.5488	99	$C_{20}H_{43}N_2O_5P$	$[M + Cl]_-$	−1.25		−0.12	0.7536
457.25979	15150.5488	99	$C_{14}H_{39}N_{10}O_3PS$	$[M-H]^-$	1.25		0.11	0.9285
457.25979	15150.5488	99	$C_{22}H_{43}N_4PS_2$	$[M-H]^-$	0.85		0.29	0.3794
457.25979	15150.5488	99	$C_{27}H_{38}O_6$	$[M-H]^-$	0.5	[Prednisolone tebutate]	0.31	0.2401
457.25979	15150.5488	99	$C_{22}H_{35}N_8OP$	$[M-H]^-$	−0.17		0.29	0.3794
457.25979	15150.5488	99	$C_{12}H_{34}N_{12}O_7$	$[M-H]^-$	−0.6		0.81	0.4036
457.25979	15150.5488	99	$C_{19}H_{44}N_2O_6P_2$	$[M-H]^-$	−0.87		−0.34	0.4139

Table S1. *Cont.*

m/z	Intensity	ID SIP (Figure S5)	Empirical formula (all-^{12}C containing peak)	Ion form	Mass error (ppm)	KEGG compound	*r*-value	*p*-value
457.25979	15150.5488	99	$C_{20}H_{38}N_6O_4S$	$[M - H]^-$	−1.01		−0.12	0.7536
457.25979	15150.5488	99	$C_{28}H_{42}OS_2$	$[M - H]^-$	−1.41		0.29	0.2665
457.26676	28713.725	100	$C_{19}H_{34}N_4O_5$	$[M + Acetate]^-$	−0.03		−0.92	0.0272
457.26676	28713.725	100	$C_{14}H_{31}N_{12}P$	$[M + Acetate]^-$	−0.7		0.00	1.0000
457.26676	28713.725	100	$C_{24}H_{42}N_2O_2S$	$[M + Cl]^-$	1.44		−0.10	0.7779
457.26676	28713.725	100	$C_{32}H_{38}$	$[M + Cl]^-$	0.02		0.94	0.0000
457.26676	28713.725	100	$C_{17}H_{42}N_8S_2$	$[M + Cl]^-$	−0.06		−1.00	0.0024
457.26676	28713.725	100	$C_{17}H_{34}N_{12}O$	$[M + Cl]^-$	−1.08		−1.00	0.0024
457.26676	28713.725	100	$C_{24}H_{44}N_2P_2$	$[M + Cl]^-$	−1.35		−0.10	0.7779
457.26676	28713.725	100	$C_{17}H_{46}N_6P_4$	$[M - H]^-$	1.29		−1.00	0.0024
457.26676	28713.725	100	$C_{31}H_{39}OP$	$[M - H]^-$	0.4		0.91	0.0000
457.26676	28713.725	100	$C_{16}H_{43}N_8OPS_2$	$[M - H]^-$	0.32		0.00	1.0000
457.26676	28713.725	100	$C_{21}H_{38}N_4O_7$	$[M - H]^-$	−0.03		−0.94	0.0017
457.26676	28713.725	100	$C_{16}H_{35}N_{12}O_2P$	$[M - H]^-$	−0.7		0.00	1.0000
457.26676	28713.725	100	$C_{23}H_{45}N_2OP_3$	$[M - H]^-$	−0.96		−0.53	0.1382
467.30118	40387.49833	101	$C_{24}H_{40}O_5$	$[M + Acetate]^-$	−0.53	[3alpha,7alpha,12beta-Trihydroxy-5beta-cholanate, Allocholic acid, Avicholate, Bile salt, Bitocholate, Cholic acid, Haemulcholate, Hyocholate, Ursocholate, Vulpecholate, alpha-Muricholate, beta-Muricholate, beta-Phocaecholate, omega-Muricholate]	−0.76	0.0061
467.30118	40387.49833	101	$C_{19}H_{37}N_8P$	$[M + Acetate]^-$	−1.18		0.38	0.2524
467.30118	40387.49833	101	$C_{21}H_{44}N_4O_5$	$[M + Cl]^-$	1.3		−0.21	0.5430
467.30118	40387.49833	101	$C_{16}H_{41}N_{12}P$	$[M + Cl]^-$	0.65		0.94	0.0001
467.30118	40387.49833	101	$C_{22}H_{48}N_4S_2$	$[M + Cl]^-$	−0.56		−0.46	0.1546

Table S1. *Cont.*

m/z	**Intensity**	**ID SIP (Figure S5)**	**Empirical formula (all-^{12}C containing peak)**	**Ion form**	**Mass error (ppm)**	**KEGG compound**	***r*-value**	***p*-value**
467.30118	40387.49833	101	$C_{15}H_{42}N_{12}OP_2$	[M − H]$^-$	1.02		0.96	0.0000
467.30118	40387.49833	101	$C_{22}H_{52}N_2P_4$	[M − H]$^-$	0.76		−0.46	0.1546
467.30118	40387.49833	101	$C_{21}H_{49}N_4OPS_2$	[M − H]$^-$	−0.19		−0.21	0.5430
467.30118	40387.49833	101	$C_{26}H_{44}O_7$	[M − H]$^-$	−0.53		−0.84	0.0011
467.30118	40387.49833	101	$C_{21}H_{41}N_8O_2P$	[M − H]$^-$	−1.18		−0.21	0.5430
463.26970	209476.87	102	$C_{18}H_{37}N_4O_4P$	[M + Acetate]$^-$	1.3		−0.08	0.8302
463.26970	209476.87	102	$C_{24}H_{36}O_5$	[M + Acetate]$^-$	−0.93	[7alpha,12alpha-Dihydroxy-3-oxochol-4-enoate, Lovastatin]	0.95	0.0000
463.26970	209476.87	102	$C_{21}H_{40}N_4O_5$	[M + Cl]$^-$	0.92		0.59	0.0441
463.26970	209476.87	102	$C_{16}H_{37}N_{12}P$	[M + Cl]$^-$	0.26		−0.48	0.2730
463.26970	209476.87	102	$C_{14}H_{40}N_{10}O_3S$	[M + Cl]$^-$	−0.56		−0.55	0.3408
463.26970	209476.87	102	$C_{22}H_{44}N_4S_2$	[M + Cl]$^-$	−0.95		0.73	0.0073
463.26970	209476.87	102	$C_{20}H_{41}N_4O_6P$	[M − H]$^-$	1.3		0.39	0.2379
463.26970	209476.87	102	$C_{15}H_{38}N_{12}OP_2$	[M − H]$^-$	0.64		−0.54	0.2649
463.26970	209476.87	102	$C_{22}H_{48}N_2P_4$	[M − H]$^-$	0.38		0.73	0.0073
463.26970	209476.87	102	$C_{21}H_{45}N_4OPS_2$	[M − H]$^-$	−0.58		0.59	0.0441
463.26970	209476.87	102	$C_{26}H_{40}O_7$	[M − H]$^-$	−0.93		0.98	0.0000
457.25979	15150.5488	103	$C_{12}H_{35}N_{10}OPS$	[M + Acetate]$^-$	1.25		0.81	0.4036
457.25979	15150.5488	103	$C_{25}H_{34}O_4$	[M + Acetate]$^-$	0.5	[17-Propylestra-1,3,5(10)-triene-3,17beta-diol diacetate, Pregna-5,16,20-triene-3beta,20-diol diacetate]	0.37	0.1979
457.25979	15150.5488	103	$C_{10}H_{30}N_{12}O_5$	[M + Acetate]$^-$	−0.6		0.93	0.2469
457.25979	15150.5488	103	$C_{17}H_{40}N_2O_4P_2$	[M + Acetate]$^-$	−0.87		−0.59	0.2219
457.25979	15150.5488	103	$C_{18}H_{34}N_6O_2S$	[M + Acetate]$^-$	−1.01		−0.47	0.2865
457.25979	15150.5488	103	$C_{15}H_{38}N_{10}O_2S$	[M + Cl]$^-$	0.87		−0.12	0.8795

Table S1. *Cont.*

m/z	Intensity	ID SIP (Figure S5)	Empirical formula (all-^{12}C containing peak)	Ion form	Mass error (ppm)	KEGG compound	r-value	p-value
457.25979	15150.5488	103	$C_{22}H_{48}OP_2S$	$[M + Cl]^-$	0.6		0.29	0.3794
457.25979	15150.5488	103	$C_{23}H_{34}N_8$	$[M + Cl]^-$	−0.55		0.37	0.2388
457.25979	15150.5488	103	$C_{20}H_{43}N_2O_5P$	$[M + Cl]^-$	−1.25		−0.12	0.7536
457.25979	15150.5488	103	$C_{14}H_{39}N_{10}O_3PS$	$[M - H]^-$	1.25		0.11	0.9285
457.25979	15150.5488	103	$C_{22}H_{43}N_4PS_2$	$[M - H]^-$	0.85		0.29	0.3794
457.25979	15150.5488	103	$C_{27}H_{38}O_6$	$[M - H]^-$	0.5	[Prednisolone tebutate]	0.31	0.2401
457.25979	15150.5488	103	$C_{22}H_{35}N_8OP$	$[M - H]^-$	−0.17		0.29	0.3794
457.25979	15150.5488	103	$C_{12}H_{34}N_{12}O_7$	$[M - H]^-$	−0.6		0.81	0.4036
457.25979	15150.5488	103	$C_{19}H_{44}N_2O_6P_2$	$[M - H]^-$	−0.87		−0.34	0.4139
457.25979	15150.5488	103	$C_{20}H_{38}N_6O_4S$	$[M - H]^-$	−1.01		−0.12	0.7536
457.25979	15150.5488	103	$C_{28}H_{42}OS_2$	$[M - H]^-$	−1.41		0.29	0.2665
473.31201	361907.5433	104	$C_{23}H_{42}O_6$	$[M + Acetate]^-$	0.03		−0.79	0.0022
473.31201	361907.5433	104	$C_{18}H_{39}N_8OP$	$[M + Acetate]^-$	−0.61		0.05	0.8770
473.31201	361907.5433	104	$C_{15}H_{43}N_{12}OP$	$[M + Cl]^-$	1.2		0.81	0.0015
473.31201	361907.5433	104	$C_{21}H_{42}N_8O_2$	$[M + Cl]^-$	−0.98		−0.63	0.0288
473.31201	361907.5433	104	$C_{25}H_{46}O_8$	$[M - H]^-$	0.03		−0.82	0.0010
473.31201	361907.5433	104	$C_{20}H_{43}N_8O_3P$	$[M - H]^-$	−0.61		−0.46	0.1318
473.31201	361907.5433	104	$C_{28}H_{47}N_2PS$	$[M - H]^-$	−1		−0.79	0.0021
471.25971	81347.33833	105	$C_{11}H_{34}N_{12}OP_2$	$[M + Acetate]^-$	1.01		0.20	0.7981
471.25971	81347.33833	105	$C_{18}H_{44}N_2P_4$	$[M + Acetate]^-$	0.76		0.99	0.0001
471.25971	81347.33833	105	$C_{17}H_{41}N_4OPS_2$	$[M + Acetate]^-$	−0.18		0.97	0.0017
471.25971	81347.33833	105	$C_{22}H_{36}O_7$	$[M + Acetate]^-$	−0.53	[Grayanotoxin I]	0.58	0.2255
471.25971	81347.33833	105	$C_{17}H_{33}N_8O_2P$	$[M + Acetate]^-$	−1.17		0.97	0.0017

Table S1. *Cont.*

m/z	Intensity	ID SIP (Figure S5)	Empirical formula (all-^{12}C containing peak)	Ion form	Mass error (ppm)	KEGG compound	*r*-value	*p*-value
471.25971	81347.33833	105	$C_{19}H_{40}N_4O_7$	[M + Cl]$^-$	1.29		0.92	0.0101
471.25971	81347.33833	105	$C_{14}H_{37}N_{12}O_2P$	[M + Cl]$^-$	0.64		0.53	0.2741
471.25971	81347.33833	105	$C_{21}H_{47}N_2OP_3$	[M + Cl]$^-$	0.38		0.68	0.1356
471.25971	81347.33833	105	$C_{20}H_{44}N_4O_2S_2$	[M + Cl]$^-$	−0.56		0.80	0.0565
471.25971	81347.33833	105	$C_{13}H_{38}N_{12}O_3P_2$	[M − H]$^-$	1.01		0.39	0.4435
471.25971	81347.33833	105	$C_{20}H_{48}N_2O_2P_4$	[M − H]$^-$	0.76		0.80	0.0565
471.25971	81347.33833	105	$C_{21}H_{42}N_6P_2S$	[M − H]$^-$	0.62		0.68	0.1356
471.25971	81347.33833	105	$C_{19}H_{45}N_4O_3PS_2$	[M − H]$^-$	−0.18		0.92	0.0101
471.25971	81347.33833	105	$C_{24}H_{40}O_9$	[M − H]$^-$	−0.53		0.44	0.3875
471.25971	81347.33833	105	$C_{19}H_{37}N_8O_4P$	[M − H]$^-$	−1.17		0.92	0.0101
465.35894	27617.706	106	$C_{26}H_{46}O_3$	[M + Acetate]$^-$	0.84		0.69	0.0277
465.35894	27617.706	106	$C_{19}H_{46}N_6OS$	[M + Acetate]$^-$	−0.63		0.28	0.4349
465.35894	27617.706	106	$C_{28}H_{50}O_5$	[M − H]$^-$	0.84	[6alpha-Hydroxy-castasterone]	0.44	0.2028
465.35894	27617.706	106	$C_{23}H_{47}N_8P$	[M − H]$^-$	0.19		0.86	0.0012
465.35894	27617.706	106	$C_{21}H_{50}N_6O_3S$	[M − H]$^-$	−0.63		0.62	0.0583
465.35894	27617.706	106	$C_{29}H_{54}S_2$	[M − H]$^-$	−1.03		0.34	0.3428
475.12277	16099.36167	107	$C_{25}H_{20}O_4S$	[M + Acetate]$^-$	1.44		−0.58	0.2262
475.12277	16099.36167	107	$C_{10}H_{24}N_8O_4S_3$	[M + Acetate]$^-$	1.36		−0.35	0.5620
475.12277	16099.36167	107	$C_{12}H_{13}N_{14}O_2P$	[M + Acetate]$^-$	1.18		0.75	0.0854
475.12277	16099.36167	107	$C_{14}H_{25}O_{12}P$	[M + Acetate]$^-$	1.15		0.89	0.0182
475.12277	16099.36167	107	$C_{18}H_{28}N_2OS_4$	[M + Acetate]$^-$	0.98		−0.21	0.6847
475.12277	16099.36167	107	$C_{19}H_{23}N_4OP_3$	[M + Acetate]$^-$	0.93		−0.32	0.5350
475.12277	16099.36167	107	$C_9H_{22}N_8O_7P_2$	[M + Acetate]$^-$	0.51		−0.91	0.0938

Table S1. *Cont.*

m/z	**Intensity**	**ID SIP (Figure S5)**	**Empirical formula (all-^{12}C containing peak)**	**Ion form**	**Mass error (ppm)**	**KEGG compound**	***r*-value**	***p*-value**
475.12277	16099.36167	107	$C_{10}H_{16}N_{12}O_5S$	[M + Acetate]$^−$	0.38		−0.35	0.5620
475.12277	16099.36167	107	$C_{17}H_{26}N_2O_4P_2S$	[M + Acetate]$^−$	0.13		−0.06	0.9122
475.12277	16099.36167	107	$C_{18}H_{20}N_6O_2S_2$	[M + Acetate]$^−$	0		−0.21	0.6847
475.12277	16099.36167	107	$C_{11}H_{29}N_6OP_5$	[M + Acetate]$^−$	−0.38		0.45	0.3690
475.12277	16099.36167	107	$C_{15}H_{29}O_7PS_2$	[M + Acetate]$^−$	−0.67		0.51	0.3002
475.12277	16099.36167	107	$C_{18}H_{12}N_{10}O_3$	[M + Acetate]$^−$	−0.99		−0.21	0.6847
475.12277	16099.36167	107	$C_{25}H_{22}O_2P_2$	[M + Acetate]$^−$	−1.24		−0.58	0.2262
475.12277	16099.36167	107	$C_{10}H_{26}N_8O_2P_2S_2$	[M + Acetate]$^−$	−1.32		−0.35	0.5620
475.12277	16099.36167	107	$C_{26}H_{16}N_4S$	[M + Acetate]$^−$	−1.37		−0.60	0.2093
475.12277	16099.36167	107	$C_{11}H_{20}N_{12}S_3$	[M + Acetate]$^−$	−1.45		0.45	0.3690
475.12277	16099.36167	107	$C_8H_{33}N_{10}OP_5$	[M + Cl]$^−$	1.42		−1.00	0.0276
475.12277	16099.36167	107	$C_{15}H_{16}N_{14}O_3$	[M + Cl]$^−$	0.82		0.51	0.3002
475.12277	16099.36167	107	$C_{17}H_{28}O_{13}$	[M + Cl]$^−$	0.79		−0.06	0.9122
475.12277	16099.36167	107	$C_{22}H_{26}N_4O_2P_2$	[M + Cl]$^−$	0.56		−0.50	0.3130
475.12277	16099.36167	107	$C_7H_{30}N_{12}O_2P_2S_2$	[M + Cl]$^−$	0.48		0.00	1.0000
475.12277	16099.36167	107	$C_{23}H_{20}N_8S$	[M + Cl]$^−$	0.43		−0.53	0.2758
475.12277	16099.36167	107	$C_{12}H_{25}N_8O_8P$	[M + Cl]$^−$	0.14		0.75	0.0854
475.12277	16099.36167	107	$C_{20}H_{29}N_2O_5PS$	[M + Cl]$^−$	−0.24		−0.40	0.4339
475.12277	16099.36167	107	$C_{10}H_{28}N_6O_{11}S$	[M + Cl]$^−$	−0.66		−0.35	0.5620
475.12277	16099.36167	107	$C_{14}H_{32}N_6O_2P_4$	[M + Cl]$^−$	−0.75		0.89	0.0182
475.12277	16099.36167	107	$C_{15}H_{26}N_{10}P_2S$	[M + Cl]$^−$	−0.88		0.51	0.3002
475.12277	16099.36167	107	$C_{18}H_{32}O_8S_2$	[M + Cl]$^−$	−1.04		−0.21	0.6847
475.12277	16099.36167	107	$C_5H_{25}N_{14}O_6PS$	[M + Cl]$^−$	−1.3		0.00	1.0000

Table S1. *Cont.*

m/z	Intensity	ID SIP (Figure S5)	Empirical formula (all-^{12}C containing peak)	Ion form	Mass error (ppm)	KEGG compound	*r*-value	*p*-value
475.12277	16099.36167	107	$C_{27}H_{24}O_6S$	$[M-H]^-$	1.44		−0.61	0.1959
475.12277	16099.36167	107	$C_{12}H_{28}N_8O_6S_3$	$[M-H]^-$	1.36		0.75	0.0854
475.12277	16099.36167	107	$C_{14}H_{17}N_{14}O_4P$	$[M-H]^-$	1.18		0.89	0.0182
475.12277	16099.36167	107	$C_{16}H_{29}O_{14}P$	$[M-H]^-$	1.15		0.17	0.7445
475.12277	16099.36167	107	$C_{20}H_{32}N_2O_3S_4$	$[M-H]^-$	0.98		−0.40	0.4339
475.12277	16099.36167	107	$C_{21}H_{27}N_4O_3P_3$	$[M-H]^-$	0.93		−0.46	0.3635
475.12277	16099.36167	107	$C_{22}H_{21}N_8OPS$	$[M-H]^-$	0.8		−0.50	0.3130
475.12277	16099.36167	107	$C_{11}H_{26}N_8O_9P_2$	$[M-H]^-$	0.51		0.45	0.3690
475.12277	16099.36167	107	$C_{14}H_{35}N_6P_3S_3$	$[M-H]^-$	0.47		0.89	0.0182
475.12277	16099.36167	107	$C_{12}H_{20}N_{12}O_7S$	$[M-H]^-$	0.38		0.75	0.0854
475.12277	16099.36167	107	$C_{19}H_{30}N_2O_6P_2S$	$[M-H]^-$	0.13		−0.32	0.5350
475.12277	16099.36167	107	$C_{20}H_{24}N_6O_4S_2$	$[M-H]^-$	0		−0.40	0.4339
475.12277	16099.36167	107	$C_{13}H_{33}N_6O_3P_5$	$[M-H]^-$	−0.38		0.98	0.0007
475.12277	16099.36167	107	$C_{28}H_{28}OS_3$	$[M-H]^-$	−0.39		−0.62	0.1851
475.12277	16099.36167	107	$C_{13}H_{32}N_8OS_5$	$[M-H]^-$	−0.46		0.98	0.0007
475.12277	16099.36167	107	$C_{14}H_{27}N_{10}OP_3S$	$[M-H]^-$	−0.51		0.89	0.0182
475.12277	16099.36167	107	$C_{17}H_{33}O_9PS_2$	$[M-H]^-$	−0.67		−0.06	0.9122
475.12277	16099.36167	107	$C_{21}H_{37}P_5S$	$[M-H]^-$	−0.77		−0.46	0.3635
475.12277	16099.36167	107	$C_{20}H_{16}N_{10}O_5$	$[M-H]^-$	−0.99		−0.40	0.4339
475.12277	16099.36167	107	$C_{27}H_{26}O_4P_2$	$[M-H]^-$	−1.24		−0.61	0.1959
475.12277	16099.36167	107	$C_{12}H_{30}N_8O_4P_2S_2$	$[M-H]^-$	−1.32		0.75	0.0854
475.12277	16099.36167	107	$C_{28}H_{20}N_4O_2S$	$[M-H]^-$	−1.37		−0.62	0.1851
475.12277	16099.36167	107	$C_{13}H_{24}N_{12}O_2S_3$	$[M-H]^-$	−1.45		0.98	0.0007

Table S1. *Cont.*

m/z	Intensity	ID SIP (Figure S5)	Empirical formula (all-^{12}C containing peak)	Ion form	Mass error (ppm)	KEGG compound	*r*-value	*p*-value
457.26676	28713.725	108	$C_{19}H_{34}N_4O_5$	$[M + Acetate]^-$	−0.03		−0.92	0.0272
457.26676	28713.725	108	$C_{14}H_{31}N_{12}P$	$[M + Acetate]^-$	−0.7		0.00	1.0000
457.26676	28713.725	108	$C_{24}H_{42}N_2O_2S$	$[M + Cl]^-$	1.44		−0.10	0.7779
457.26676	28713.725	108	$C_{32}H_{38}$	$[M + Cl]^-$	0.02		0.94	0.0000
457.26676	28713.725	108	$C_{17}H_{42}N_8S_2$	$[M + Cl]^-$	−0.06		−1.00	0.0024
457.26676	28713.725	108	$C_{17}H_{34}N_{12}O$	$[M + Cl]^-$	−1.08		−1.00	0.0024
457.26676	28713.725	108	$C_{24}H_{44}N_2P_2$	$[M + Cl]^-$	−1.35		−0.10	0.7779
457.26676	28713.725	108	$C_{17}H_{46}N_6P_4$	$[M - H]^-$	1.29		−1.00	0.0024
457.26676	28713.725	108	$C_{31}H_{39}OP$	$[M - H]^-$	0.4		0.91	0.0000
457.26676	28713.725	108	$C_{16}H_{43}N_8OPS_2$	$[M - H]^-$	0.32		0.00	1.0000
457.26676	28713.725	108	$C_{21}H_{38}N_4O_7$	$[M - H]^-$	−0.03		−0.94	0.0017
457.26676	28713.725	108	$C_{16}H_{35}N_{12}O_2P$	$[M - H]^-$	−0.7		0.00	1.0000
457.26676	28713.725	108	$C_{23}H_{45}N_2OP_3$	$[M - H]^-$	−0.96		−0.53	0.1382
467.30118	40387.49833	109	$C_{24}H_{40}O_5$	$[M + Acetate]^-$	−0.53	[3alpha,7alpha,12beta-Trihydroxy-5beta-cholanate, Allocholic acid, Avicholate, Bile salt, Bitocholate, Cholic acid, Haemulcholate, Hyocholate, Ursocholate, Vulpecholate, alpha-Muricholate, beta-Muricholate, beta-Phocaecholate, omega-Muricholate]	−0.76	0.0061
467.30118	40387.49833	109	$C_{19}H_{37}N_8P$	$[M + Acetate]^-$	−1.18		0.38	0.2524
467.30118	40387.49833	109	$C_{21}H_{44}N_4O_5$	$[M + Cl]^-$	1.3		−0.21	0.5430
467.30118	40387.49833	109	$C_{16}H_{41}N_{12}P$	$[M + Cl]^-$	0.65		0.94	0.0001
467.30118	40387.49833	109	$C_{22}H_{48}N_4S_2$	$[M + Cl]^-$	−0.56		−0.46	0.1546
467.30118	40387.49833	109	$C_{15}H_{42}N_{12}OP_2$	$[M - H]^-$	1.02		0.96	0.0000

Table S1. *Cont.*

m/z	Intensity	ID SIP (Figure S5)	Empirical formula (all-^{12}C containing peak)	Ion form	Mass error (ppm)	KEGG compound	*r*-value	*p*-value
467.30118	40387.49833	109	$C_{22}H_{52}N_2P_4$	$[M-H]^-$	0.76		−0.46	0.1546
467.30118	40387.49833	109	$C_{21}H_{49}N_4OPS_2$	$[M-H]^-$	−0.19		−0.21	0.5430
467.30118	40387.49833	109	$C_{26}H_{44}O_7$	$[M-H]^-$	−0.53		−0.84	0.0011
467.30118	40387.49833	109	$C_{21}H_{41}N_8O_2P$	$[M-H]^-$	−1.18		−0.21	0.5430
473.29075	26306.27167	110	$C_{13}H_{39}N_{10}OPS$	$[M + Acetate]^-$	0.49		0.00	1.0000
473.29075	26306.27167	110	$C_{26}H_{38}O_4$	$[M + Acetate]^-$	−0.24	[Lupulone]	0.74	0.0214
473.29075	26306.27167	110	$C_{11}H_{34}N_{12}O_5$	$[M + Acetate]^-$	−1.3		0.00	1.0000
473.29075	26306.27167	110	$C_{16}H_{42}N_{10}O_2S$	$[M + Cl]^-$	0.12		−0.56	0.4388
473.29075	26306.27167	110	$C_{24}H_{38}N_8$	$[M + Cl]^-$	−1.25		0.53	0.1426
473.29075	26306.27167	110	$C_{17}H_{40}N_{12}P_2$	$[M-H]^-$	1.29		−0.60	0.2844
473.29075	26306.27167	110	$C_{15}H_{43}N_{10}O_3PS$	$[M-H]^-$	0.49		0.00	1.0000
473.29075	26306.27167	110	$C_{23}H_{47}N_4PS_2$	$[M-H]^-$	0.1		0.39	0.2937
473.29075	26306.27167	110	$C_{28}H_{42}O_6$	$[M-H]^-$	−0.24		0.91	0.0006
473.29075	26306.27167	110	$C_{23}H_{39}N_8OP$	$[M-H]^-$	−0.88		0.39	0.2937
473.29075	26306.27167	110	$C_{13}H_{38}N_{12}O_7$	$[M-H]^-$	−1.3		0.00	1.0000
481.31693	13797.74867	111	$C_{25}H_{42}O_5$	$[M + Acetate]^-$	−0.31		−0.56	0.2476
481.31693	13797.74867	111	$C_{20}H_{39}N_8P$	$[M + Acetate]^-$	−0.94		−0.16	0.7689
481.31693	13797.74867	111	$C_{22}H_{46}N_4O_5$	$[M + Cl]^-$	1.47		−0.41	0.4248
481.31693	13797.74867	111	$C_{17}H_{43}N_{12}P$	$[M + Cl]^-$	0.84		0.86	0.0265
481.31693	13797.74867	111	$C_{23}H_{50}N_4S_2$	$[M + Cl]^-$	−0.34		−0.47	0.3417
481.31693	13797.74867	111	$C_{23}H_{42}N_8O$	$[M + Cl]^-$	−1.31		−0.47	0.3417
481.31693	13797.74867	111	$C_{16}H_{44}N_{12}OP_2$	$[M-H]^-$	1.2		0.93	0.0070
481.31693	13797.74867	111	$C_{23}H_{54}N_2P_4$	$[M-H]^-$	0.95		−0.47	0.3417

Table S1. *Cont.*

m/z	Intensity	ID SIP (Figure S5)	Empirical formula (all-^{12}C containing peak)	Ion form	Mass error (ppm)	KEGG compound	*r*-value	*p*-value
481.31693	13797.74867	111	$C_{22}H_{51}N_4OPS_2$	$[M - H]^-$	0.03		−0.41	0.4248
481.31693	13797.74867	111	$C_{27}H_{46}O_7$	$[M - H]^-$	−0.31		−0.61	0.1996
481.31693	13797.74867	111	$C_{22}H_{43}N_8O_2P$	$[M - H]^-$	−0.94		−0.41	0.4248
481.28025	144511.525	112	$C_{18}H_{39}N_4O_5P$	$[M + Acetate]^-$	1.22		0.29	0.5248
481.28025	144511.525	112	$C_{13}H_{36}N_{12}P_2$	$[M + Acetate]^-$	0.59		−0.91	0.2788
481.28025	144511.525	112	$C_{19}H_{43}N_4PS_2$	$[M + Acetate]^-$	−0.59		0.44	0.3210
481.28025	144511.525	112	$C_{24}H_{38}O_6$	$[M + Acetate]^-$	−0.92		0.51	0.2407
481.28025	144511.525	112	$C_{31}H_{43}P$	$[M + Cl]^-$	1.27		−0.56	0.1918
481.28025	144511.525	112	$C_{21}H_{42}N_4O_6$	$[M + Cl]^-$	0.86		0.77	0.0421
481.28025	144511.525	112	$C_{16}H_{39}N_{12}OP$	$[M + Cl]^-$	0.22		−0.26	0.6231
481.28025	144511.525	112	$C_{23}H_{49}N_2P_3$	$[M + Cl]^-$	−0.03		0.82	0.0228
481.28025	144511.525	112	$C_{22}H_{46}N_4OS_2$	$[M + Cl]^-$	−0.95		0.89	0.0073
481.28025	144511.525	112	$C_{20}H_{43}N_4O_7P$	$[M - H]^-$	1.22		0.61	0.1492
481.28025	144511.525	112	$C_{15}H_{40}N_{12}O_2P_2$	$[M - H]^-$	0.59		−0.60	0.2888
481.28025	144511.525	112	$C_{22}H_{50}N_2OP_4$	$[M - H]^-$	0.34		0.89	0.0073
481.28025	144511.525	112	$C_{21}H_{47}N_4O_2PS_2$	$[M - H]^-$	−0.59		0.77	0.0421
481.28025	144511.525	112	$C_{26}H_{42}O_8$	$[M - H]^-$	−0.92	[Fusicoccin H]	−0.08	0.8609
479.33838	20201.553	113	$C_{26}H_{44}O_4$	$[M + Acetate]^-$	1.18	[6-Ethylchenodeoxycholic acid]	1.00	0.0000
479.33838	20201.553	113	$C_{19}H_{44}N_6O_2S$	$[M + Acetate]^-$	−0.25		−0.57	0.1100
479.33838	20201.553	113	$C_{24}H_{44}N_8$	$[M + Cl]^-$	0.18		0.81	0.0025
479.33838	20201.553	113	$C_{28}H_{48}O_6$	$[M - H]^-$	1.18	[24-epi-Brassinolide, Brassinolide]	0.77	0.0055
479.33838	20201.553	113	$C_{23}H_{45}N_8OP$	$[M - H]^-$	0.55		0.62	0.0438
479.33838	20201.553	113	$C_{21}H_{48}N_6O_4S$	$[M - H]^-$	−0.25		0.19	0.5820

Table S1. *Cont.*

m/z	Intensity	ID SIP (Figure S5)	Empirical formula (all-^{12}C containing peak)	Ion form	Mass error (ppm)	KEGG compound	*r*-value	*p*-value
479.33838	20201.553	113	$C_{29}H_{52}OS_2$	$[M-H]^-$	−0.63		0.54	0.0841
483.29577	39760.412	114	$C_{18}H_{41}N_4O_5P$	$[M + Acetate]^-$	0.95		−0.46	0.2481
483.29577	39760.412	114	$C_{13}H_{38}N_{12}P_2$	$[M + Acetate]^-$	0.32		−0.98	0.0244
483.29577	39760.412	114	$C_{19}H_{45}N_4PS_2$	$[M + Acetate]^-$	−0.85		−0.24	0.5662
483.29577	39760.412	114	$C_{24}H_{40}O_6$	$[M + Acetate]^-$	−1.19	[alpha-Phocaecholic acid]	0.93	0.0007
483.29577	39760.412	114	$C_{31}H_{45}P$	$[M + Cl]^-$	0.99		0.32	0.4346
483.29577	39760.412	114	$C_{21}H_{44}N_4O_6$	$[M + Cl]^-$	0.58		0.39	0.3452
483.29577	39760.412	114	$C_{16}H_{41}N_{12}OP$	$[M + Cl]^-$	−0.05		−0.85	0.0161
483.29577	39760.412	114	$C_{23}H_{51}N_2P_3$	$[M + Cl]^-$	−0.3		0.92	0.0011
483.29577	39760.412	114	$C_{22}H_{48}N_4OS_2$	$[M + Cl]^-$	−1.21		0.72	0.0426
483.29577	39760.412	114	$C_{30}H_{46}OP_2$	$[M-H]^-$	1.36		0.38	0.3566
483.29577	39760.412	114	$C_{20}H_{45}N_4O_7P$	$[M-H]^-$	0.95		0.04	0.9174
483.29577	39760.412	114	$C_{15}H_{42}N_{12}O_2P_2$	$[M-H]^-$	0.32		−0.95	0.0041
483.29577	39760.412	114	$C_{22}H_{52}N_2OP_4$	$[M-H]^-$	0.06		0.72	0.0426
483.29577	39760.412	114	$C_{21}H_{49}N_4O_2PS_2$	$[M-H]^-$	−0.85		0.39	0.3452
483.29577	39760.412	114	$C_{26}H_{44}O_8$	$[M-H]^-$	−1.19		0.72	0.0428
511.39964	6149.618667	115	$C_{25}H_{56}N_4O_4$	$[M + Cl]^-$	0.16		0.77	0.0241
511.39964	6149.618667	115	$C_{27}H_{48}N_{10}$	$[M-H]^-$	1.13		0.65	0.0789
511.39964	6149.618667	115	$C_{19}H_{54}N_{12}P_2$	$[M-H]^-$	−0.09		0.58	0.1295
507.33228	7318.3336	116	$C_{21}H_{45}N_4O_4P$	$[M + Acetate]^-$	1.15		−0.44	0.2367
507.33228	7318.3336	116	$C_{27}H_{44}O_5$	$[M + Acetate]^-$	−0.88	[2-Deoxyecdysone, Convallagenin A, Digitogenin]	0.86	0.0031
507.33228	7318.3336	116	$C_{22}H_{41}N_8P$	$[M + Acetate]^-$	−1.49		−0.01	0.9713
507.33228	7318.3336	116	$C_{24}H_{48}N_4O_5$	$[M + Cl]^-$	0.8		0.53	0.1447

Table S1. *Cont.*

m/z	Intensity	ID SIP (Figure S5)	Empirical formula (all-^{12}C containing peak)	Ion form	Mass error (ppm)	KEGG compound	*r*-value	*p*-value
507.33228	7318.3336	116	$C_{19}H_{45}N_{12}P$	$[M + Cl]^-$	0.2		−0.84	0.0189
507.33228	7318.3336	116	$C_{25}H_{52}N_4S_2$	$[M + Cl]^-$	−0.91		0.66	0.0507
507.33228	7318.3336	116	$C_{23}H_{49}N_4O_6P$	$[M - H]^-$	1.15		0.32	0.3979
507.33228	7318.3336	116	$C_{18}H_{46}N_{12}OP_2$	$[M - H]^-$	0.55		−0.89	0.0180
507.33228	7318.3336	116	$C_{25}H_{56}N_2P_4$	$[M - H]^-$	0.31		0.66	0.0507
507.33228	7318.3336	116	$C_{24}H_{53}N_4OPS_2$	$[M - H]^-$	−0.56		0.53	0.1447
507.33228	7318.3336	116	$C_{29}H_{48}O_7$	$[M - H]^-$	−0.88		0.97	0.0000
507.33228	7318.3336	116	$C_{24}H_{45}N_8O_2P$	$[M - H]^-$	−1.49		0.53	0.1447
515.35882	83953.423	117	$C_{15}H_{46}N_{12}P_2$	$[M + Acetate]^-$	1.17		0.76	0.0784
515.35882	83953.423	117	$C_{26}H_{48}O_6$	$[M + Acetate]^-$	−0.24		−0.65	0.1581
515.35882	83953.423	117	$C_{21}H_{45}N_8OP$	$[M + Acetate]^-$	−0.83		−0.47	0.3466
515.35882	83953.423	117	$C_{23}H_{52}N_4O_6$	$[M + Cl]^-$	1.42		−0.59	0.2216
515.35882	83953.423	117	$C_{18}H_{49}N_{12}OP$	$[M + Cl]^-$	0.83		0.17	0.7405
515.35882	83953.423	117	$C_{24}H_{48}N_8O_2$	$[M + Cl]^-$	−1.17		−0.62	0.1917
515.35882	83953.423	117	$C_{17}H_{50}N_{12}O_2P_2$	$[M - H]^-$	1.17		0.61	0.2026
515.35882	83953.423	117	$C_{28}H_{52}O_8$	$[M - H]^-$	−0.24		−0.67	0.1414
515.35882	83953.423	117	$C_{23}H_{49}N_8O_3P$	$[M - H]^-$	−0.83		−0.59	0.2216
515.35882	83953.423	117	$C_{31}H_{53}N_2PS$	$[M - H]^-$	−1.19		−0.69	0.1299
509.38446	9373.352833	118	$C_{22}H_{51}N_4O_3P$	$[M + Acetate]^-$	1.43		0.38	0.1850
509.38446	9373.352833	118	$C_{28}H_{50}O_4$	$[M + Acetate]^-$	−0.6	[6-Deoxocastasterone]	0.93	0.0000
509.38446	9373.352833	118	$C_{25}H_{54}N_4O_4$	$[M + Cl]^-$	1.08		0.91	0.0000
509.38446	9373.352833	118	$C_{24}H_{55}N_4O_5P$	$[M - H]^-$	1.43		0.77	0.0012

Table S1. *Cont.*

m/z	**Intensity**	**ID SIP (Figure S5)**	**Empirical formula (all-^{12}C containing peak)**	**Ion form**	**Mass error (ppm)**	**KEGG compound**	***r*-value**	***p*-value**
509.38$_{44}$6	9373.352833	118	$C_{19}H_{52}N_{12}P_2$	$[M-H]^-$	0.83		−0.57	0.0662
509.38$_{44}$6	9373.352833	118	$C_{30}H_{54}O_6$	$[M-H]^-$	−0.6		0.64	0.0129
509.38$_{44}$6	9373.352833	118	$C_{25}H_{51}N_8OP$	$[M-H]^-$	−1.19		0.91	0.0000
519.15505	67172.39333	119	$C_7H_{21}N_{14}O_8P$	$[M + Acetate]^-$	1.45		0.00	1.0000
519.15505	67172.39333	119	$C_{14}H_{31}N_4O_7P_3$	$[M + Acetate]^-$	1.22		0.50	0.2049
519.15505	67172.39333	119	$C_{15}H_{25}N_8O_5PS$	$[M + Acetate]^-$	1.1		0.82	0.0067
519.15505	67172.39333	119	$C_{23}H_{29}N_2O_2PS_2$	$[M + Acetate]^-$	0.74		−0.46	0.1854
519.15505	67172.39333	119	$C_5H_{24}N_{12}O_{11}S$	$[M + Acetate]^-$	0.72		0.00	1.0000
519.15505	67172.39333	119	$C_9H_{28}N_{12}O_2P_4$	$[M + Acetate]^-$	0.63		−0.96	0.1870
519.15505	67172.39333	119	$C_{16}H_{38}N_2OP_6$	$[M + Acetate]^-$	0.4		0.96	0.0000
519.15505	67172.39333	119	$C_{13}H_{28}N_6O_8S_2$	$[M + Acetate]^-$	0.36		0.01	0.9802
519.15505	67172.39333	119	$C_{21}H_{32}O_5S_3$	$[M + Acetate]^-$	0.01		0.04	0.9092
519.15505	67172.39333	119	$C_{23}H_{21}N_6O_3P$	$[M + Acetate]^-$	−0.16		−0.46	0.1854
519.15505	67172.39333	119	$C_8H_{25}N_{14}O_3PS_2$	$[M + Acetate]^-$	−0.22		0.00	1.0000
519.15505	67172.39333	119	$C_{15}H_{35}N_4O_2P_3S_2$	$[M + Acetate]^-$	−0.46		0.82	0.0067
519.15505	67172.39333	119	$C_{31}H_{25}PS$	$[M + Acetate]^-$	−0.51		−0.80	0.0052
519.15505	67172.39333	119	$C_{13}H_{20}N_{10}O_9$	$[M + Acetate]^-$	−0.54		0.01	0.9802
519.15505	67172.39333	119	$C_{16}H_{29}N_8PS_3$	$[M + Acetate]^-$	−0.58		0.96	0.0000
519.15505	67172.39333	119	$C_{20}H_{30}O_8P_2$	$[M + Acetate]^-$	−0.77		0.36	0.3028
519.15505	67172.39333	119	$C_{21}H_{24}N_4O_6S$	$[M + Acetate]^-$	−0.89		0.04	0.9092
519.15505	67172.39333	119	$C_{14}H_{32}N_6O_3S_4$	$[M + Acetate]^-$	−1.31		0.50	0.2049
519.15505	67172.39333	119	$C_{15}H_{27}N_8O_3P_3$	$[M + Acetate]^-$	−1.36		0.82	0.0067
519.15505	67172.39333	119	$C_{16}H_{21}N_{12}OPS$	$[M + Acetate]^-$	−1.48		0.96	0.0000

Table S1. *Cont.*

m/z	**Intensity**	**ID SIP (Figure S5)**	**Empirical formula (all-^{12}C containing peak)**	**Ion form**	**Mass error (ppm)**	**KEGG compound**	***r*-value**	***p*-value**
519.15505	67172.39333	119	$C_{20}H_{25}N_{10}O_3P$	$[M + Cl]^-$	1.49		0.36	0.3028
519.15505	67172.39333	119	$C_{27}H_{35}O_2P_3$	$[M + Cl]^-$	1.26		−0.78	0.0075
519.15505	67172.39333	119	$C_{28}H_{29}N_4PS$	$[M + Cl]^-$	1.14		−0.80	0.0056
519.15505	67172.39333	119	$C_{10}H_{24}N_{14}O_9$	$[M + Cl]^-$	1.11		−0.92	0.0780
519.15505	67172.39333	119	$C_{13}H_{33}N_{12}PS_3$	$[M + Cl]^-$	1.07		0.01	0.9802
519.15505	67172.39333	119	$C_{17}H_{34}N_4O_8P_2$	$[M + Cl]^-$	0.88		0.97	0.0000
519.15505	67172.39333	119	$C_{18}H_{28}N_8O_6S$	$[M + Cl]^-$	0.76		0.87	0.0010
519.15505	67172.39333	119	$C_{26}H_{32}N_2O_3S_2$	$[M + Cl]^-$	0.41		−0.75	0.0125
519.15505	67172.39333	119	$C_{12}H_{31}N_{12}O_3P_3$	$[M + Cl]^-$	0.29		−0.54	0.2658
519.15505	67172.39333	119	$C_{19}H_{41}N_2O_2P_5$	$[M + Cl]^-$	0.06		0.66	0.0379
519.15505	67172.39333	119	$C_{19}H_{40}N_4S_5$	$[M + Cl]^-$	−0.01		0.66	0.0379
519.15505	67172.39333	119	$C_{20}H_{35}N_6P_3S$	$[M + Cl]^-$	−0.06		0.36	0.3028
519.15505	67172.39333	119	$C_{10}H_{34}N_{10}O_6P_2S$	$[M + Cl]^-$	−0.44		−0.92	0.0780
519.15505	67172.39333	119	$C_{26}H_{24}N_6O_4$	$[M + Cl]^-$	−0.49		−0.75	0.0125
519.15505	67172.39333	119	$C_{11}H_{28}N_{14}O_4S_2$	$[M + Cl]^-$	−0.56		−0.84	0.0731
519.15505	67172.39333	119	$C_{18}H_{38}N_4O_3P_2S_2$	$[M + Cl]^-$	−0.79		0.87	0.0010
519.15505	67172.39333	119	$C_{34}H_{28}OS$	$[M + Cl]^-$	−0.84		−0.78	0.0074
519.15505	67172.39333	119	$C_{19}H_{32}N_8OS_3$	$[M + Cl]^-$	−0.91		0.66	0.0379
519.15505	67172.39333	119	$C_{23}H_{33}O_9P$	$[M + Cl]^-$	−1.11		−0.46	0.1854
519.15505	67172.39333	119	$C_{13}H_{32}N_4O_{15}$	$[M + Cl]^-$	−1.49		0.01	0.9802
519.15505	67172.39333	119	$C_{27}H_{30}N_4OP_2S$	$[M - H]^-$	1.48		−0.78	0.0075
519.15505	67172.39333	119	$C_9H_{25}N_{14}O_{10}P$	$[M - H]^-$	1.45		−0.96	0.1870
519.15505	67172.39333	119	$C_{12}H_{34}N_{12}OP_2S_3$	$[M - H]^-$	1.41		−0.54	0.2658

Table S1. *Cont.*

m/z	**Intensity**	**ID SIP (Figure S5)**	**Empirical formula (all-^{12}C containing peak)**	**Ion form**	**Mass error (ppm)**	**KEGG compound**	***r*-value**	***p*-value**
519.15505	67172.39333	119	$C_{16}H_{35}N_4O_9P_3$	$[M - H]^-$	1.22		0.96	0.0000
519.15505	67172.39333	119	$C_{19}H_{44}N_2P_4S_3$	$[M - H]^-$	1.18		0.66	0.0379
519.15505	67172.39333	119	$C_{17}H_{29}N_8O_7PS$	$[M - H]^-$	1.1		0.97	0.0000
519.15505	67172.39333	119	$C_{25}H_{33}N_2O_4PS_2$	$[M - H]^-$	0.74		−0.69	0.0260
519.15505	67172.39333	119	$C_7H_{28}N_{12}O_{13}S$	$[M - H]^-$	0.72		0.00	1.0000
519.15505	67172.39333	119	$C_{11}H_{32}N_{12}O_4P_4$	$[M - H]^-$	0.63		−0.84	0.0731
519.15505	67172.39333	119	$C_{28}H_{16}N_{12}$	$[M - H]^-$	0.46		−0.80	0.0056
519.15505	67172.39333	119	$C_{18}H_{42}N_2O_3P_6$	$[M - H]^-$	0.4		0.87	0.0010
519.15505	67172.39333	119	$C_{15}H_{32}N_6O_{10}S_2$	$[M - H]^-$	0.36		0.82	0.0067
519.15505	67172.39333	119	$C_{18}H_{41}N_4OPS_5$	$[M - H]^-$	0.32		0.87	0.0010
519.15505	67172.39333	119	$C_{19}H_{36}N_6OP_4S$	$[M - H]^-$	0.28		0.66	0.0379
519.15505	67172.39333	119	$C_{23}H_{36}O_7S_3$	$[M - H]^-$	0.01		−0.46	0.1854
519.15505	67172.39333	119	$C_{25}H_{25}N_6O_5P$	$[M - H]^-$	−0.16		−0.69	0.0260
519.15505	67172.39333	119	$C_{10}H_{29}N_{14}O_5PS_2$	$[M - H]^-$	−0.22		−0.92	0.0780
519.15505	67172.39333	119	$C_{17}H_{39}N_4O_4P_3S_2$	$[M - H]^-$	−0.46		0.97	0.0000
519.15505	67172.39333	119	$C_{33}H_{29}O_2PS$	$[M - H]^-$	−0.51		−0.79	0.0065
519.15505	67172.39333	119	$C_{15}H_{24}N_{10}O_{11}$	$[M - H]^-$	−0.54		0.82	0.0067
519.15505	67172.39333	119	$C_{18}H_{33}N_8O_2PS_3$	$[M - H]^-$	−0.58		0.87	0.0010
519.15505	67172.39333	119	$C_{20}H_{22}N_{14}P_2$	$[M - H]^-$	−0.74		0.36	0.3028
519.15505	67172.39333	119	$C_{22}H_{34}O_{10}P_2$	$[M - H]^-$	−0.77		−0.24	0.5009
519.15505	67172.39333	119	$C_{23}H_{28}N_4O_8S$	$[M - H]^-$	−0.89		−0.46	0.1854
519.15505	67172.39333	119	$C_{16}H_{36}N_6O_5S_4$	$[M - H]^-$	−1.31		0.96	0.0000
519.15505	67172.39333	119	$C_{17}H_{31}N_8O_5P_3$	$[M - H]^-$	−1.36		0.97	0.0000

Table S1. *Cont.*

m/z	Intensity	ID SIP (Figure S5)	Empirical formula (all-^{12}C containing peak)	Ion form	Mass error (ppm)	KEGG compound	*r*-value	*p*-value
519.15505	67172.39333	119	$C_{18}H_{25}N_{12}O_3PS$	$[M-H]^-$	−1.48		0.87	0.0010
521.15192	38452.1225	120	$C_{17}H_{14}N_{14}O_3$	$[M + Acetate]^-$	1.41		0.81	0.0082
521.15192	38452.1225	120	$C_{19}H_{26}O_{13}$	$[M + Acetate]^-$	1.38		0.56	0.1172
521.15192	38452.1225	120	$C_{24}H_{24}N_4O_2P_2$	$[M + Acetate]^-$	1.18		0.22	0.5747
521.15192	38452.1225	120	$C_9H_{28}N_{12}O_2P_2S_2$	$[M + Acetate]^-$	1.11		−0.38	0.4569
521.15192	38452.1225	120	$C_{25}H_{18}N_8S$	$[M + Acetate]^-$	1.06		0.18	0.6405
521.15192	38452.1225	120	$C_{16}H_{38}N_2OP_4S_2$	$[M + Acetate]^-$	0.88		0.93	0.0003
521.15192	38452.1225	120	$C_{14}H_{23}N_8O_8P$	$[M + Acetate]^-$	0.8		0.93	0.0003
521.15192	38452.1225	120	$C_{22}H_{27}N_2O_5PS$	$[M + Acetate]^-$	0.45		0.31	0.4111
521.15192	38452.1225	120	$C_{12}H_{26}N_6O_{11}S$	$[M + Acetate]^-$	0.07		0.43	0.2463
521.15192	38452.1225	120	$C_{15}H_{35}N_4O_2PS_4$	$[M + Acetate]^-$	0.03		0.99	0.0000
521.15192	38452.1225	120	$C_{16}H_{30}N_6O_2P_4$	$[M + Acetate]^-$	−0.02		0.93	0.0003
521.15192	38452.1225	120	$C_{17}H_{24}N_{10}P_2S$	$[M + Acetate]^-$	−0.14		0.81	0.0082
521.15192	38452.1225	120	$C_{20}H_{30}O_8S_2$	$[M + Acetate]^-$	−0.28		0.46	0.2126
521.15192	38452.1225	120	$C_7H_{23}N_{14}O_6PS$	$[M + Acetate]^-$	−0.52		−0.46	0.5409
521.15192	38452.1225	120	$C_{14}H_{33}N_4O_5P_3S$	$[M + Acetate]^-$	−0.75		0.93	0.0003
521.15192	38452.1225	120	$C_{30}H_{23}O_3P$	$[M + Acetate]^-$	−0.8		0.07	0.8539
521.15192	38452.1225	120	$C_{15}H_{27}N_8O_3PS_2$	$[M + Acetate]^-$	−0.87		0.99	0.0000
521.15192	38452.1225	120	$C_{20}H_{22}N_4O_9$	$[M + Acetate]^-$	−1.18		0.46	0.2126
521.15192	38452.1225	120	$C_{23}H_{31}N_2PS_3$	$[M + Acetate]^-$	−1.22		0.26	0.4984
521.15192	38452.1225	120	$C_9H_{30}N_{12}P_4S$	$[M + Acetate]^-$	−1.33		−0.38	0.4569
521.15192	38452.1225	120	$C_{20}H_{44}OP_6$	$[M + Cl]^-$	1.39		0.46	0.2126
521.15192	38452.1225	120	$C_{17}H_{34}N_4O_8S_2$	$[M + Cl]^-$	1.36		0.81	0.0082

Table S1. *Cont.*

m/z	Intensity	ID SIP (Figure S5)	Empirical formula (all-^{12}C containing peak)	Ion form	Mass error (ppm)	KEGG compound	*r*-value	*p*-value
521.15192	38452.1225	120	$C_{27}H_{27}N_4O_3P$	$[M + Cl]^-$	0.84		0.13	0.7453
521.15192	38452.1225	120	$C_{12}H_{31}N_{12}O_3PS_2$	$[M + Cl]^-$	0.77		0.43	0.2463
521.15192	38452.1225	120	$C_{19}H_{41}N_2O_2P_3S_2$	$[M + Cl]^-$	0.54		0.56	0.1172
521.15192	38452.1225	120	$C_{17}H_{26}N_8O_9$	$[M + Cl]^-$	0.46		0.81	0.0082
521.15192	38452.1225	120	$C_{20}H_{35}N_6PS_3$	$[M + Cl]^-$	0.42		0.46	0.2126
521.15192	38452.1225	120	$C_{25}H_{30}N_2O_6S$	$[M + Cl]^-$	0.11		0.18	0.6405
521.15192	38452.1225	120	$C_{18}H_{38}N_4O_3S_4$	$[M + Cl]^-$	−0.31		0.68	0.0446
521.15192	38452.1225	120	$C_{19}H_{33}N_6O_3P_3$	$[M + Cl]^-$	−0.35		0.56	0.1172
521.15192	38452.1225	120	$C_{20}H_{27}N_{10}OPS$	$[M + Cl]^-$	−0.47		0.46	0.2126
521.15192	38452.1225	120	$C_{27}H_{37}P_3S$	$[M + Cl]^-$	−0.71		0.13	0.7453
521.15192	38452.1225	120	$C_9H_{32}N_{10}O_9P_2$	$[M + Cl]^-$	−0.73		−0.38	0.4569
521.15192	38452.1225	120	$C_{10}H_{26}N_{14}O_7S$	$[M + Cl]^-$	−0.85		−0.21	0.6439
521.15192	38452.1225	120	$C_{17}H_{36}N_4O_6P_2S$	$[M + Cl]^-$	−1.09		0.81	0.0082
521.15192	38452.1225	120	$C_{33}H_{26}O_4$	$[M + Cl]^-$	−1.14		0.04	0.9258
521.15192	38452.1225	120	$C_{18}H_{30}N_8O_4S_2$	$[M + Cl]^-$	−1.2		0.68	0.0446
521.15192	38452.1225	120	$C_{19}H_{18}N_{14}O_5$	$[M - H]^-$	1.41		0.56	0.1172
521.15192	38452.1225	120	$C_{21}H_{30}O_{15}$	$[M - H]^-$	1.38		0.38	0.3142
521.15192	38452.1225	120	$C_{26}H_{28}N_4O_4P_2$	$[M - H]^-$	1.18		0.15	0.6969
521.15192	38452.1225	120	$C_{11}H_{32}N_{12}O_4P_2S_2$	$[M - H]^-$	1.11		0.07	0.8741
521.15192	38452.1225	120	$C_{27}H_{22}N_8O_2S$	$[M - H]^-$	1.06		0.13	0.7453
521.15192	38452.1225	120	$C_{18}H_{42}N_2O_3P_4S_2$	$[M - H]^-$	0.88		0.68	0.0446
521.15192	38452.1225	120	$C_{16}H_{27}N_8O_{10}P$	$[M - H]^-$	0.8		0.93	0.0003
521.15192	38452.1225	120	$C_{19}H_{36}N_6OP_2S_3$	$[M - H]^-$	0.76		0.56	0.1172

Table S1. *Cont.*

m/z	Intensity	ID SIP (Figure S5)	Empirical formula (all-^{12}C containing peak)	Ion form	Mass error (ppm)	KEGG compound	*r*-value	*p*-value
521.15192	38452.1225	120	$C_{24}H_{31}N_2O_7PS$	[M − H]$^−$	0.45		0.22	0.5747
521.15192	38452.1225	120	$C_6H_{26}N_{12}O_{16}$	[M − H]$^−$	0.42		−0.72	0.4862
521.15192	38452.1225	120	$C_{14}H_{30}N_6O_{13}S$	[M − H]$^−$	0.07		0.93	0.0003
521.15192	38452.1225	120	$C_{17}H_{39}N_4O_4PS_4$	[M − H]$^−$	0.03		0.81	0.0082
521.15192	38452.1225	120	$C_{18}H_{34}N_6O_4P_4$	[M − H]$^−$	−0.02		0.68	0.0446
521.15192	38452.1225	120	$C_{19}H_{28}N_{10}O_2P_2S$	[M − H]$^−$	−0.14		0.56	0.1172
521.15192	38452.1225	120	$C_{20}H_{22}N_{14}S_2$	[M − H]$^−$	−0.26		0.46	0.2126
521.15192	38452.1225	120	$C_{22}H_{34}O_{10}S_2$	[M − H]$^−$	−0.28		0.31	0.4111
521.15192	38452.1225	120	$C_{26}H_{38}OP_4S$	[M − H]$^−$	−0.37		0.15	0.6969
521.15192	38452.1225	120	$C_9H_{27}N_{14}O_8PS$	[M − H]$^−$	−0.52		−0.38	0.4569
521.15192	38452.1225	120	$C_{16}H_{37}N_4O_7P_3S$	[M − H]$^−$	−0.75		0.93	0.0003
521.15192	38452.1225	120	$C_{32}H_{27}O_5P$	[M − H]$^−$	−0.8		0.05	0.9048
521.15192	38452.1225	120	$C_{17}H_{31}N_8O_5PS_2$	[M − H]$^−$	−0.87		0.81	0.0082
521.15192	38452.1225	120	$C_{22}H_{26}N_4O_{11}$	[M − H]$^−$	−1.18		0.31	0.4111
521.15192	38452.1225	120	$C_{25}H_{35}N_2O_2PS_3$	[M − H]$^−$	−1.22		0.18	0.6405
521.15192	38452.1225	120	$C_{11}H_{34}N_{12}O_2P_4S$	[M − H]$^−$	−1.33		0.07	0.8741
521.15192	38452.1225	120	$C_{27}H_{24}N_8P_2$	[M − H]$^−$	−1.38		0.13	0.7453
527.33751	57657.2125	121	$C_{24}H_{45}N_4O_3P$	[M + Acetate]$^−$	1.38		0.28	0.3837
527.33751	57657.2125	121	$C_{22}H_{48}N_2O_6S$	[M + Acetate]$^−$	0.66		−0.12	0.7438
527.33751	57657.2125	121	$C_{17}H_{45}N_{10}OPS$	[M + Acetate]$^−$	0.08		−0.91	0.0329
527.33751	57657.2125	121	$C_{30}H_{44}O_4$	[M + Acetate]$^−$	−0.58	[3-Oxoglycyrrhetinate]	0.95	0.0000
527.33751	57657.2125	121	$C_{27}H_{48}N_4O_4$	[M + Cl]$^−$	1.05		0.67	0.0117
527.33751	57657.2125	121	$C_{20}H_{48}N_{10}O_2S$	[M + Cl]$^−$	−0.25		−0.68	0.0629

Table S1. *Cont.*

m/z	Intensity	ID SIP (Figure S5)	Empirical formula (all-^{12}C containing peak)	Ion form	Mass error (ppm)	KEGG compound	*r*-value	*p*-value
527.33751	57657.2125	121	$C_{27}H_{58}OP_2S$	$[M + Cl]^-$	−0.48		0.67	0.0117
527.33751	57657.2125	121	$C_{28}H_{44}N_8$	$[M + Cl]^-$	−1.49		0.78	0.0016
527.33751	57657.2125	121	$C_{26}H_{49}N_4O_5P$	$[M - H]^-$	1.38		0.56	0.0477
527.33751	57657.2125	121	$C_{21}H_{46}N_{12}P_2$	$[M - H]^-$	0.8		−0.37	0.3310
527.33751	57657.2125	121	$C_{24}H_{52}N_2O_8S$	$[M - H]^-$	0.66		0.28	0.3837
527.33751	57657.2125	121	$C_{19}H_{49}N_{10}O_3PS$	$[M - H]^-$	0.08		−0.91	0.0049
527.33751	57657.2125	121	$C_{27}H_{53}N_4PS_2$	$[M - H]^-$	−0.27		0.67	0.0117
527.33751	57657.2125	121	$C_{32}H_{48}O_6$	$[M - H]^-$	−0.58		0.97	0.0000
527.33751	57657.2125	121	$C_{27}H_{45}N_8OP$	$[M - H]^-$	−1.15		0.67	0.0117
539.43103	5999.149833	122	$C_{30}H_{56}O_4$	$[M + Acetate]^-$	−1.27		−0.01	0.9727
539.43103	5999.149833	122	$C_{27}H_{60}N_4O_4$	$[M + Cl]^-$	0.32		0.35	0.3205
539.43103	5999.149833	122	$C_{29}H_{52}N_{10}$	$[M - H]^-$	1.24		0.08	0.8226
539.43103	5999.149833	122	$C_{21}H_{58}N_{12}P_2$	$[M - H]^-$	0.08		0.80	0.0054
539.43103	5999.149833	122	$C_{32}H_{60}O_6$	$[M - H]^-$	−1.27		−0.14	0.6917
543.35306	15062.22667	123	$C_{24}H_{40}N_{10}O$	$[M + Acetate]^-$	1.01		−0.23	0.5759
543.35306	15062.22667	123	$C_{31}H_{50}P_2$	$[M + Acetate]^-$	0.79		−0.53	0.1760
543.35306	15062.22667	123	$C_{21}H_{49}N_4O_6P$	$[M + Acetate]^-$	0.43		0.17	0.6902
543.35306	15062.22667	123	$C_{16}H_{46}N_{12}OP_2$	$[M + Acetate]^-$	−0.13		0.85	0.0078
543.35306	15062.22667	123	$C_{27}H_{48}O_7$	$[M + Acetate]^-$	−1.47		−0.42	0.3034
543.35306	15062.22667	123	$C_{34}H_{53}OP$	$[M + Cl]^-$	0.47		−0.57	0.1365
543.35306	15062.22667	123	$C_{24}H_{52}N_4O_7$	$[M + Cl]^-$	0.11		−0.23	0.5759
543.35306	15062.22667	123	$C_{19}H_{49}N_{12}O_2P$	$[M + Cl]^-$	−0.46		0.65	0.0803
543.35306	15062.22667	123	$C_{26}H_{59}N_2OP_3$	$[M + Cl]^-$	−0.68		−0.37	0.3662

Table S1. *Cont.*

m/z	Intensity	ID SIP (Figure S5)	Empirical formula (all-^{12}C containing peak)	Ion form	Mass error (ppm)	KEGG compound	*r*-value	*p*-value
543.35306	15062.22667	123	$C_{25}H_{56}N_4O_2S_2$	$[M + Cl]^-$	−1.49		−0.31	0.4532
543.35306	15062.22667	123	$C_{26}H_{44}N_{10}O_3$	$[M - H]^-$	1.01		−0.37	0.3662
543.35306	15062.22667	123	$C_{33}H_{54}O_2P_2$	$[M - H]^-$	0.79		−0.56	0.1470
543.35306	15062.22667	123	$C_{34}H_{48}N_4S$	$[M - H]^-$	0.68		−0.57	0.1365
543.35306	15062.22667	123	$C_{19}H_{52}N_{12}S_3$	$[M - H]^-$	0.61		0.65	0.0803
543.35306	15062.22667	123	$C_{23}H_{53}N_4O_8P$	$[M - H]^-$	0.43		−0.14	0.7497
543.35306	15062.22667	123	$C_{18}H_{50}N_{12}O_3P_2$	$[M - H]^-$	−0.13		0.88	0.0036
543.35306	15062.22667	123	$C_{26}H_{54}N_6P_2S$	$[M - H]^-$	−0.47		−0.37	0.3662
543.35306	15062.22667	123	$C_{29}H_{52}O_9$	$[M - H]^-$	−1.47		−0.49	0.2228
539.35834	25747.61833	124	$C_{25}H_{40}N_{10}$	$[M + Acetate]^-$	1.38		0.86	0.0059
539.35834	25747.61833	124	$C_{22}H_{49}N_4O_5P$	$[M + Acetate]^-$	0.79		0.81	0.0154
539.35834	25747.61833	124	$C_{17}H_{46}N_{12}P_2$	$[M + Acetate]^-$	0.23		−0.37	0.3645
539.35834	25747.61833	124	$C_{23}H_{53}N_4PS_2$	$[M + Acetate]^-$	−0.82		0.89	0.0027
539.35834	25747.61833	124	$C_{28}H_{48}O_6$	$[M + Acetate]^-$	−1.12	[24-epi-Brassinolide, Brassinolide]	0.61	0.1079
539.35834	25747.61833	124	$C_{25}H_{52}N_4O_6$	$[M + Cl]^-$	0.47		0.86	0.0059
539.35834	25747.61833	124	$C_{20}H_{49}N_{12}OP$	$[M + Cl]^-$	−0.1		0.42	0.3020
539.35834	25747.61833	124	$C_{27}H_{59}N_2P_3$	$[M + Cl]^-$	−0.32		0.70	0.0545
539.35834	25747.61833	124	$C_{26}H_{56}N_4OS_2$	$[M + Cl]^-$	−1.14		0.79	0.0209
539.35834	25747.61833	124	$C_{27}H_{44}N_{10}O_2$	$[M - H]^-$	1.38		0.70	0.0545
539.35834	25747.61833	124	$C_{34}H_{54}OP_2$	$[M - H]^-$	1.16		0.27	0.5257
539.35834	25747.61833	124	$C_{24}H_{53}N_4O_7P$	$[M - H]^-$	0.79		0.91	0.0019
539.35834	25747.61833	124	$C_{19}H_{50}N_{12}O_2P_2$	$[M - H]^-$	0.23		0.16	0.7121
539.35834	25747.61833	124	$C_{26}H_{60}N_2OP_4$	$[M - H]^-$	0		0.79	0.0209

Table S1. *Cont.*

m/z	Intensity	ID SIP (Figure S5)	Empirical formula (all-^{12}C containing peak)	Ion form	Mass error (ppm)	KEGG compound	*r*-value	*p*-value
539.35834	25747.61833	124	$C_{25}H_{57}N_4O_2PS_2$	$[M - H]^-$	−0.82		0.86	0.0059
539.35834	25747.61833	124	$C_{30}H_{52}O_8$	$[M - H]^-$	−1.12		0.46	0.2500
553.37380	35429.475	125	$C_{26}H_{42}N_{10}$	$[M + Acetate]^-$	1.01		−0.63	0.0120
553.37380	35429.475	125	$C_{23}H_{51}N_4O_5P$	$[M + Acetate]^-$	0.43		−0.24	0.3960
553.37380	35429.475	125	$C_{18}H_{48}N_{12}P_2$	$[M + Acetate]^-$	−0.12		0.79	0.0004
553.37380	35429.475	125	$C_{24}H_{55}N_4PS_2$	$[M + Acetate]^-$	−1.14		−0.41	0.1285
553.37380	35429.475	125	$C_{29}H_{50}O_6$	$[M + Acetate]^-$	−1.43		−0.71	0.0030
553.37380	35429.475	125	$C_{26}H_{54}N_4O_6$	$[M + Cl]^-$	0.11		−0.63	0.0120
553.37380	35429.475	125	$C_{21}H_{51}N_{12}OP$	$[M + Cl]^-$	−0.44		0.19	0.4862
553.37380	35429.475	125	$C_{28}H_{61}N_2P_3$	$[M + Cl]^-$	−0.66		−0.70	0.0034
553.37380	35429.475	125	$C_{27}H_{58}N_4OS_2$	$[M + Cl]^-$	−1.46		−0.68	0.0053
553.37380	35429.475	125	$C_{28}H_{46}N_{10}O_2$	$[M - H]^-$	1.01		−0.70	0.0034
553.37380	35429.475	125	$C_{25}H_{55}N_4O_7P$	$[M - H]^-$	0.43		−0.54	0.0371
553.37380	35429.475	125	$C_{20}H_{52}N_{12}O_2P_2$	$[M - H]^-$	−0.12		0.42	0.1212
553.37380	35429.475	125	$C_{27}H_{62}N_2OP_4$	$[M - H]^-$	−0.34		−0.68	0.0053
553.37380	35429.475	125	$C_{26}H_{59}N_4O_2PS_2$	$[M - H]^-$	−1.14		−0.63	0.0120
553.37380	35429.475	125	$C_{31}H_{54}O_8$	$[M - H]^-$	−1.43		−0.69	0.0041
563.35818	263391.525	126	$C_{27}H_{40}N_{10}$	$[M + Acetate]^-$	1.04		0.33	0.3842
563.35818	263391.525	126	$C_{24}H_{49}N_4O_5P$	$[M + Acetate]^-$	0.47		0.01	0.9848
563.35818	263391.525	126	$C_{19}H_{46}N_{12}P_2$	$[M + Acetate]^-$	−0.07		−0.90	0.0972
563.35818	263391.525	126	$C_{25}H_{53}N_4PS_2$	$[M + Acetate]^-$	−1.07		0.10	0.7989
563.35818	263391.525	126	$C_{30}H_{48}O_6$	$[M + Acetate]^-$	−1.36		0.76	0.0169
563.35818	263391.525	126	$C_{27}H_{52}N_4O_6$	$[M + Cl]^-$	0.16		0.33	0.3842

Table S1. *Cont.*

m/z	Intensity	ID SIP (Figure S5)	Empirical formula (all-^{12}C containing peak)	Ion form	Mass error (ppm)	KEGG compound	*r*-value	*p*-value
563.35818	263391.525	126	$C_{22}H_{49}N_{12}OP$	$[M+Cl]^{-}$	−0.38		−0.68	0.0899
563.35818	263391.525	126	$C_{29}H_{59}N_{2}P_{3}$	$[M+Cl]^{-}$	−0.59		0.61	0.0786
563.35818	263391.525	126	$C_{20}H_{52}N_{10}O_{4}S$	$[M+Cl]^{-}$	−1.05		−0.92	0.0288
563.35818	263391.525	126	$C_{28}H_{56}N_{4}OS_{2}$	$[M+Cl]^{-}$	−1.38		0.47	0.2042
563.35818	263391.525	126	$C_{29}H_{44}N_{10}O_{2}$	$[M-H]^{-}$	1.04		0.61	0.0786
563.35818	263391.525	126	$C_{26}H_{53}N_{4}O_{7}P$	$[M-H]^{-}$	0.47		0.21	0.5919
563.35818	263391.525	126	$C_{21}H_{50}N_{12}O_{2}P_{2}$	$[M-H]^{-}$	−0.07		−0.91	0.0119
563.35818	263391.525	126	$C_{28}H_{60}N_{2}OP_{4}$	$[M-H]^{-}$	−0.28		0.47	0.2042
563.35818	263391.525	126	$C_{27}H_{57}N_{4}O_{2}PS_{2}$	$[M-H]^{-}$	−1.07		0.33	0.3842
563.35818	263391.525	126	$C_{32}H_{52}O_{8}$	$[M-H]^{-}$	−1.36		0.98	0.0000

Figure S5. Stable isotope patterns located in mass spectra collected from ^{13}C-labeled cultures of *Alexandrium tamarense*.

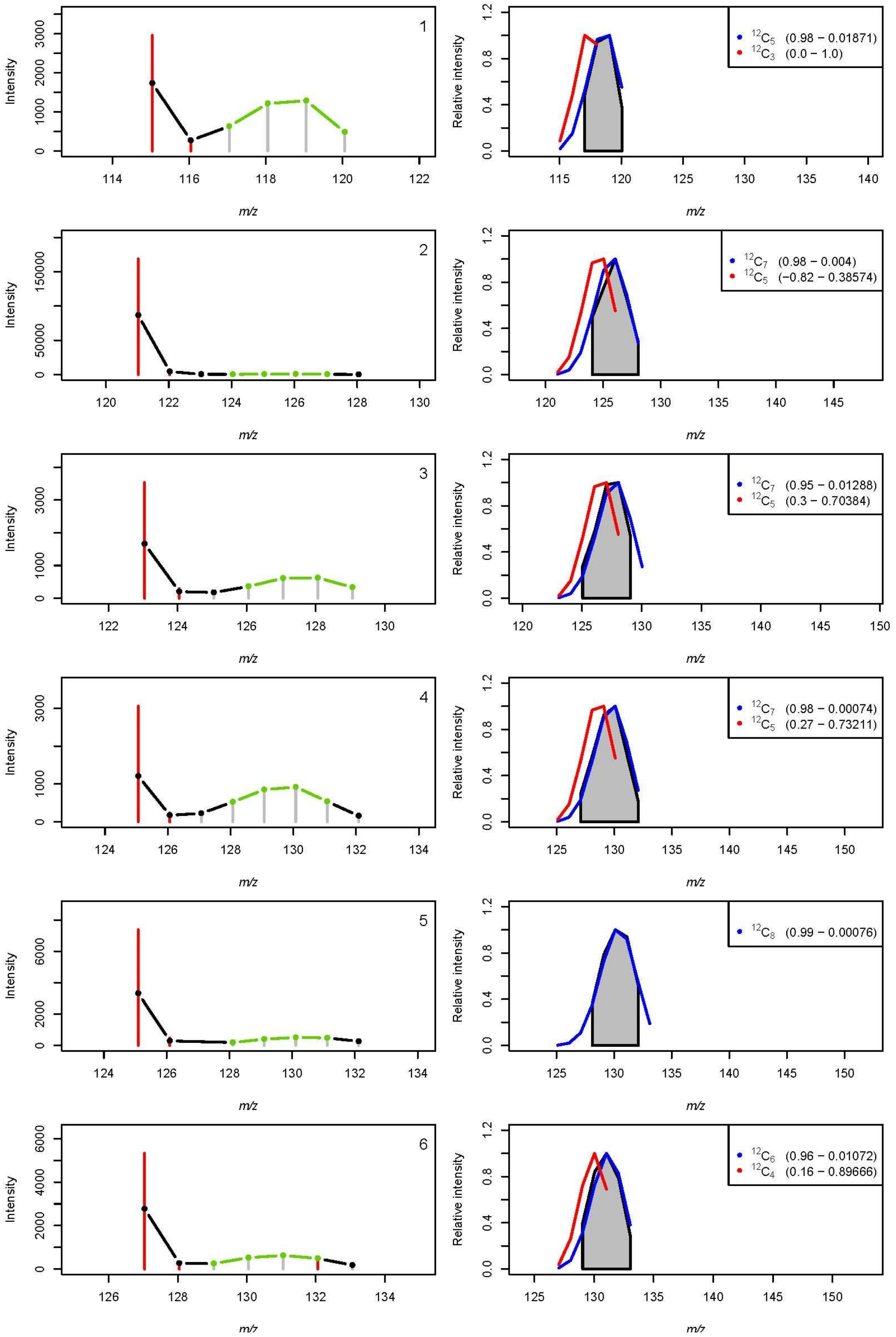

Figure S5. *Cont.*

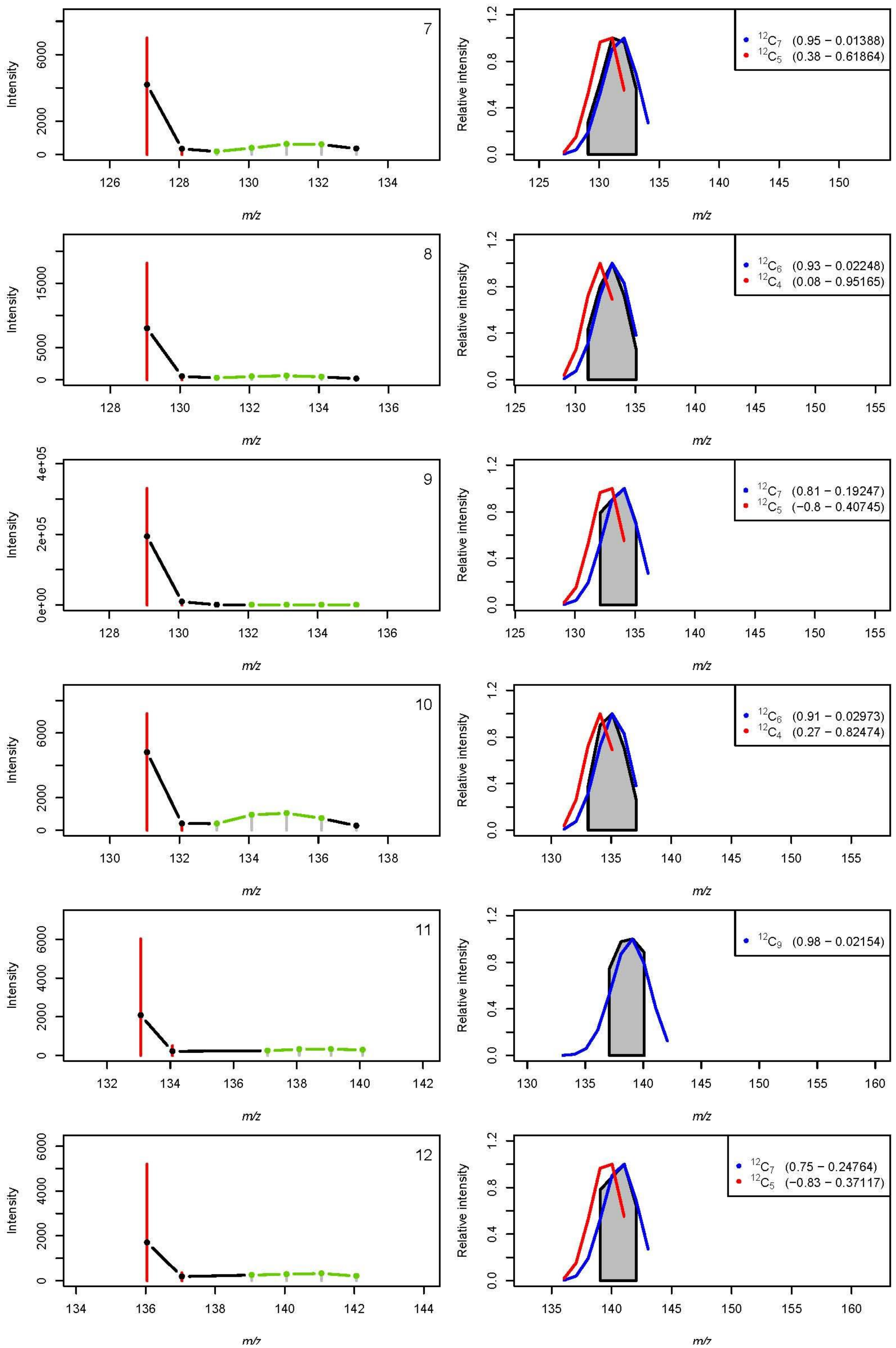

Figure S5. *Cont.*

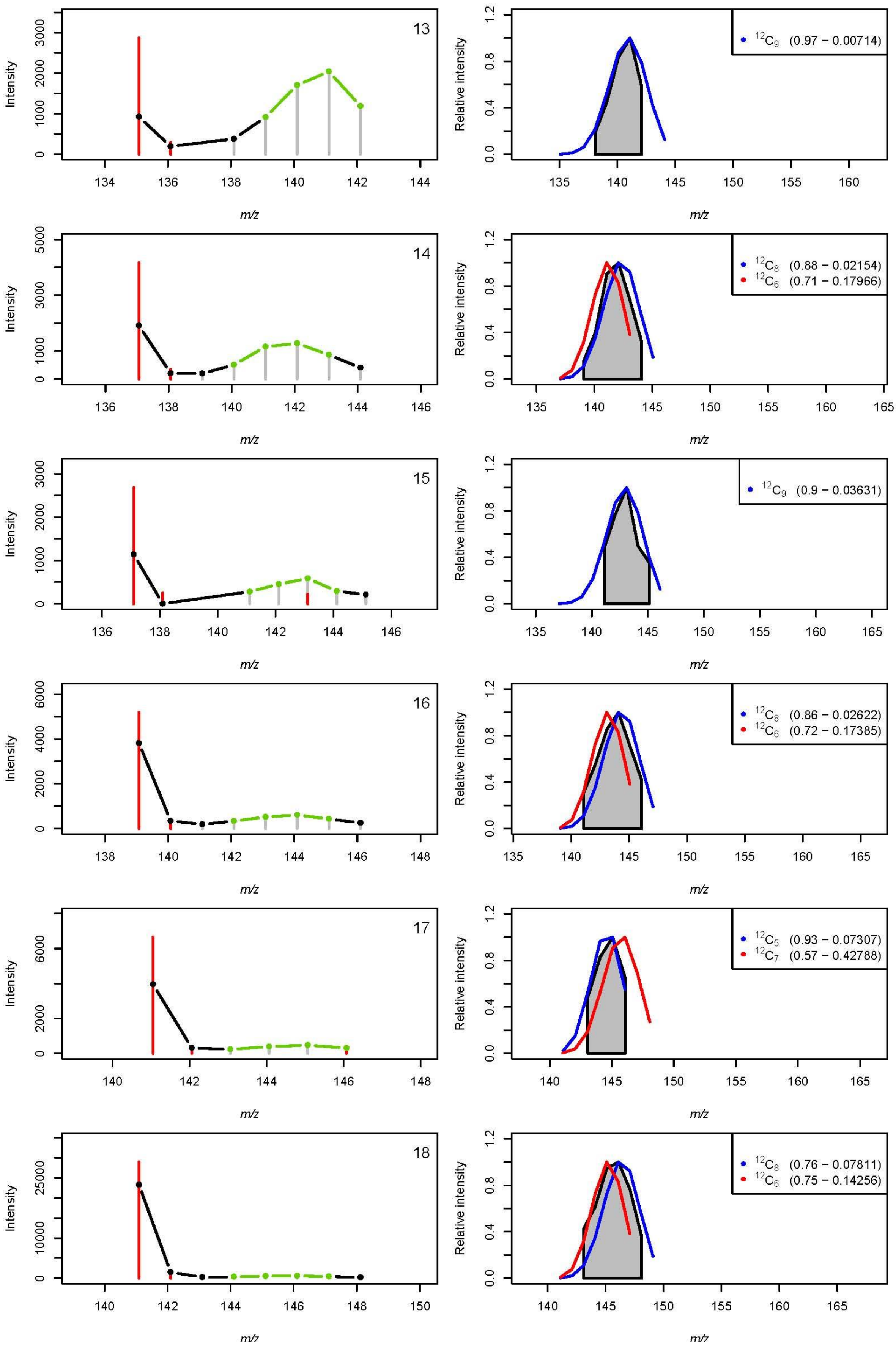

Figure S5. *Cont.*

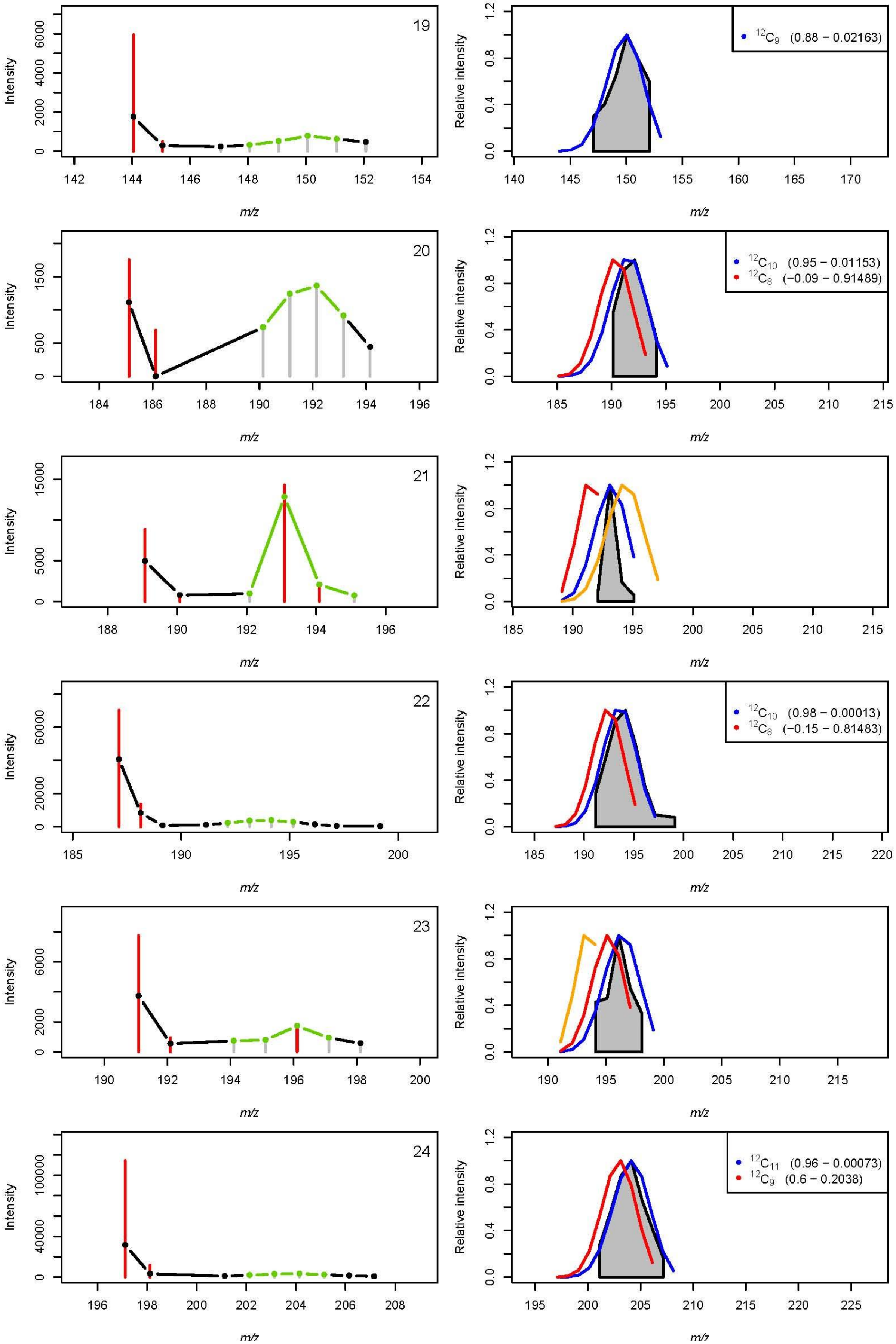

Figure S5. *Cont.*

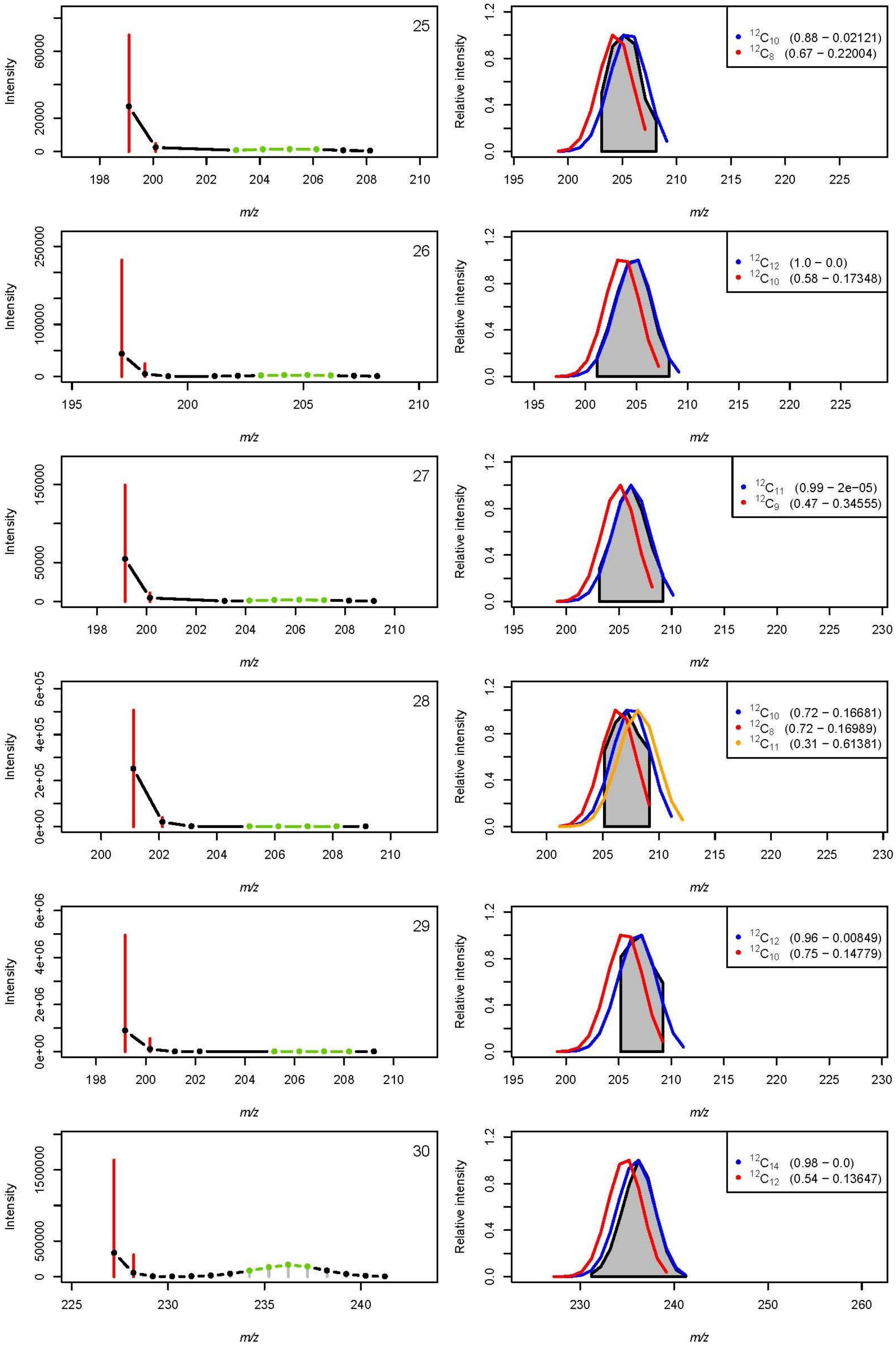

Figure S5. *Cont.*

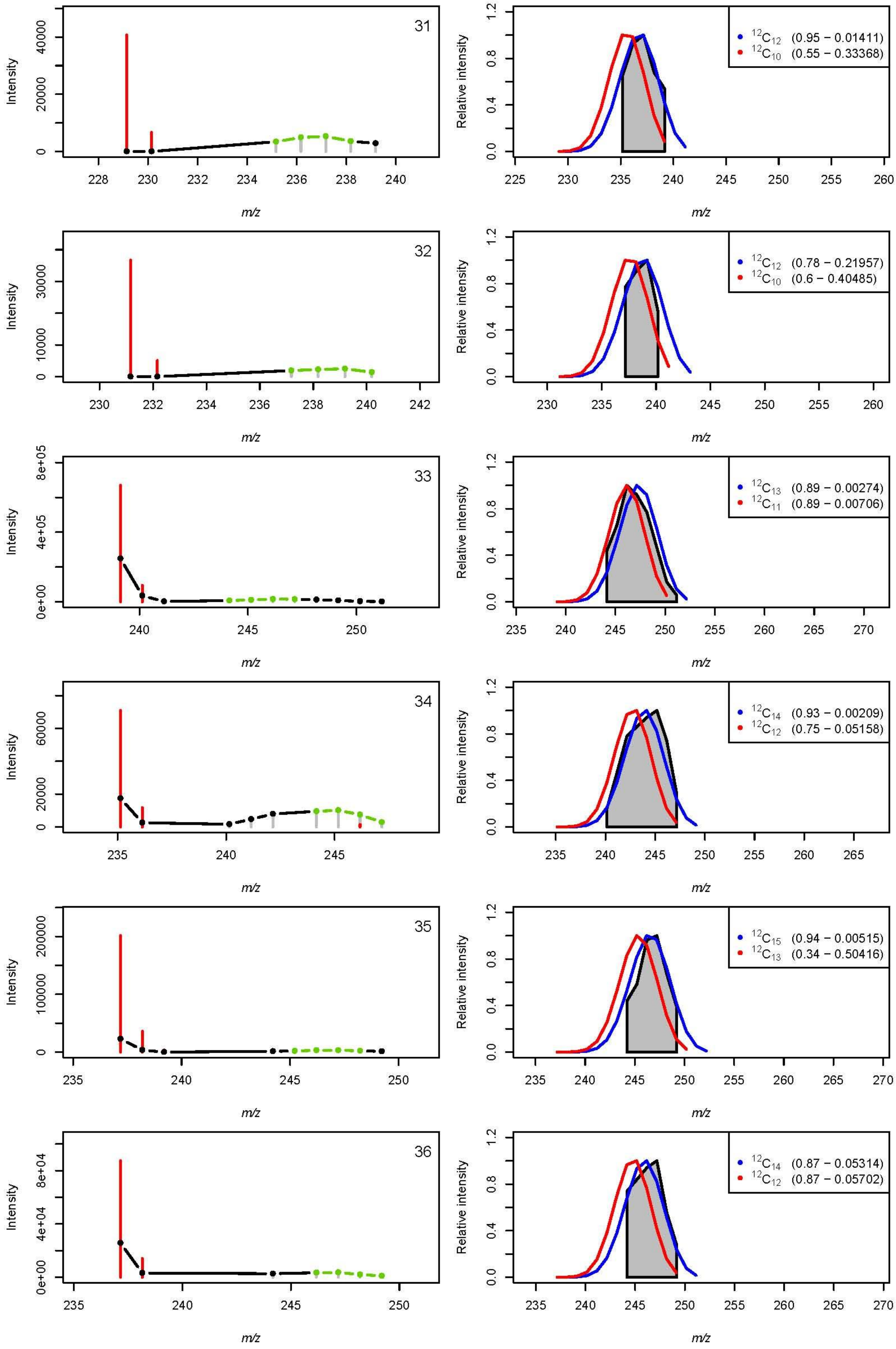

Figure S5. *Cont.*

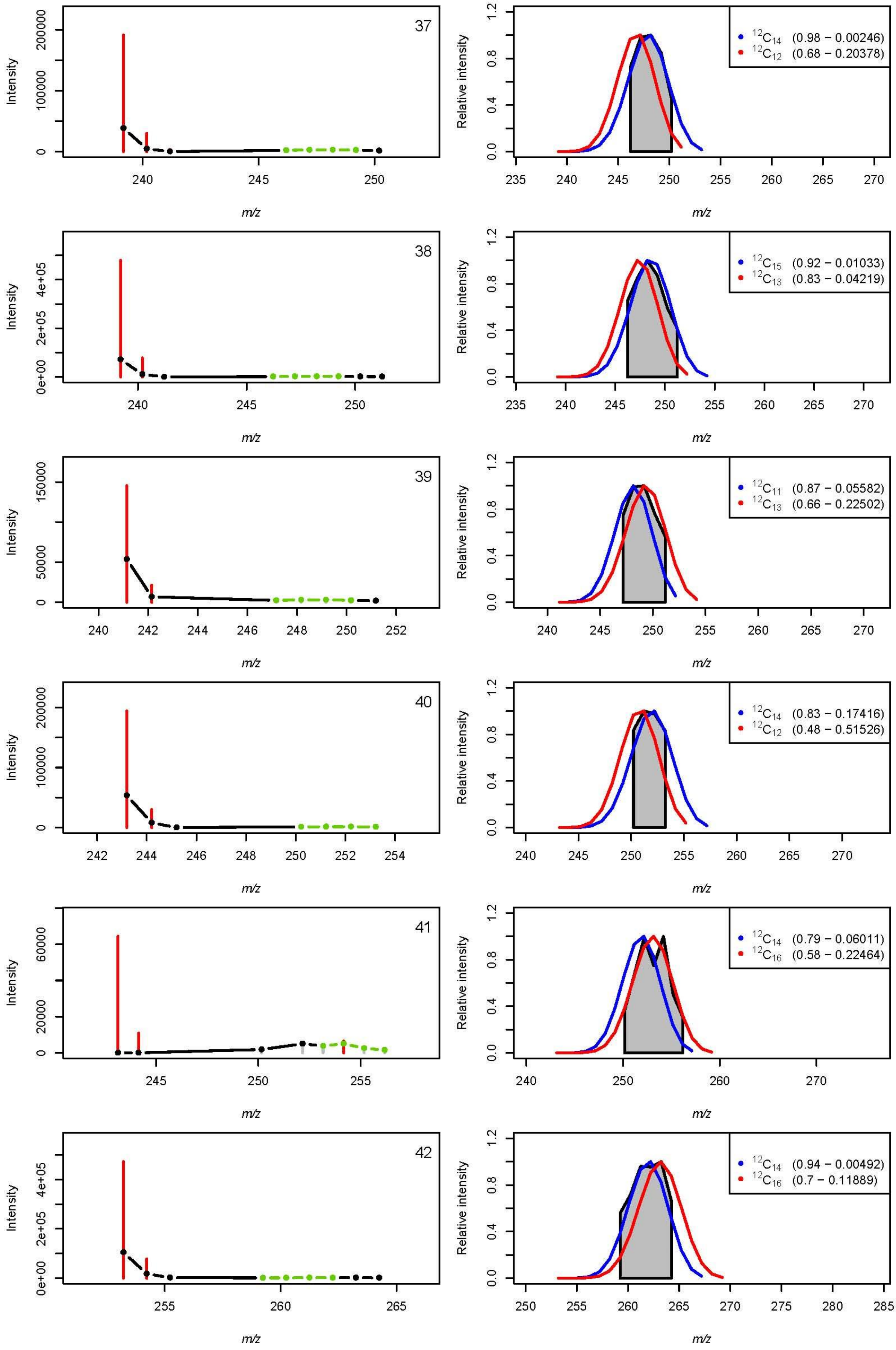

Figure S5. *Cont.*

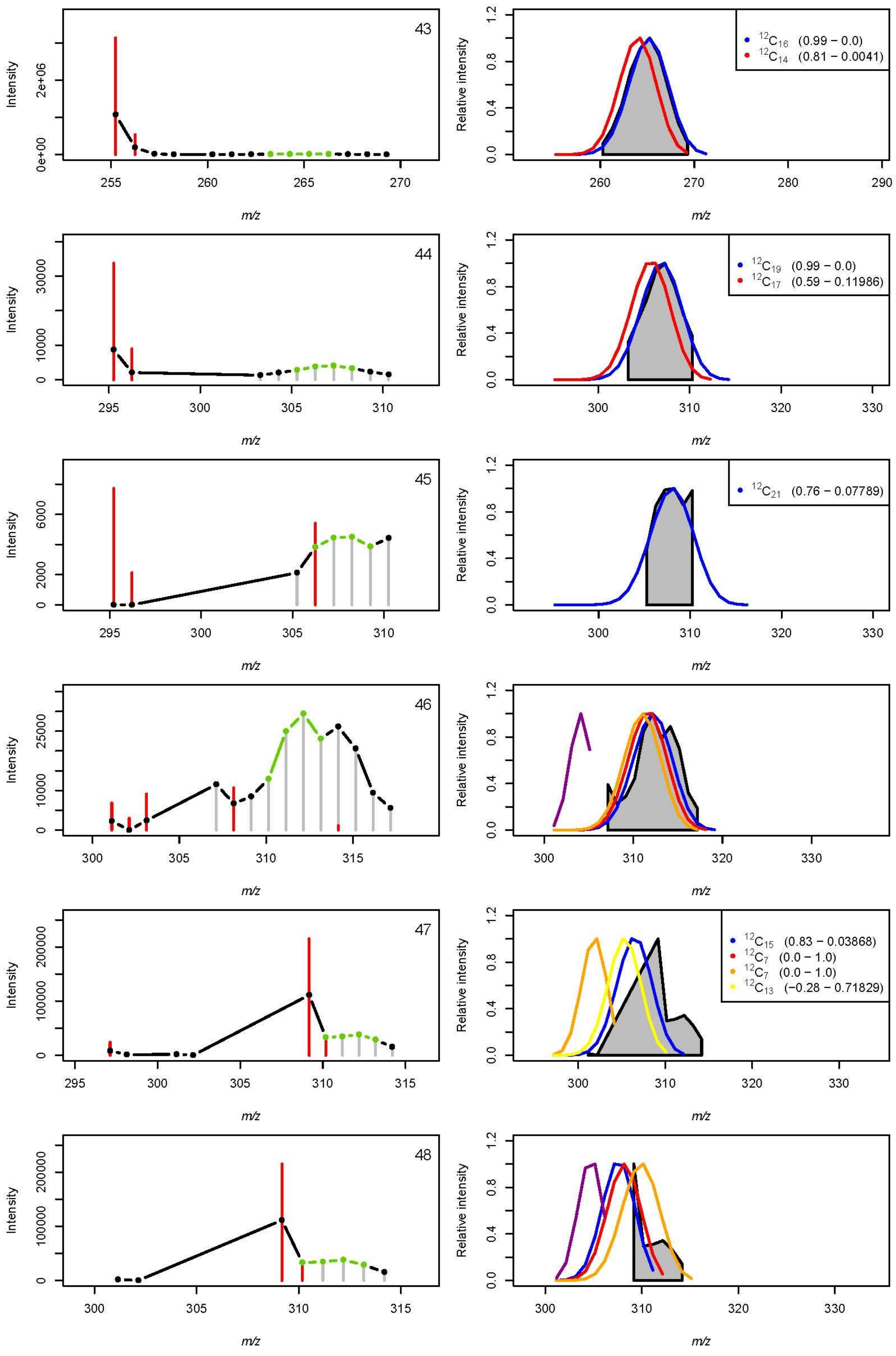

Figure S5. *Cont.*

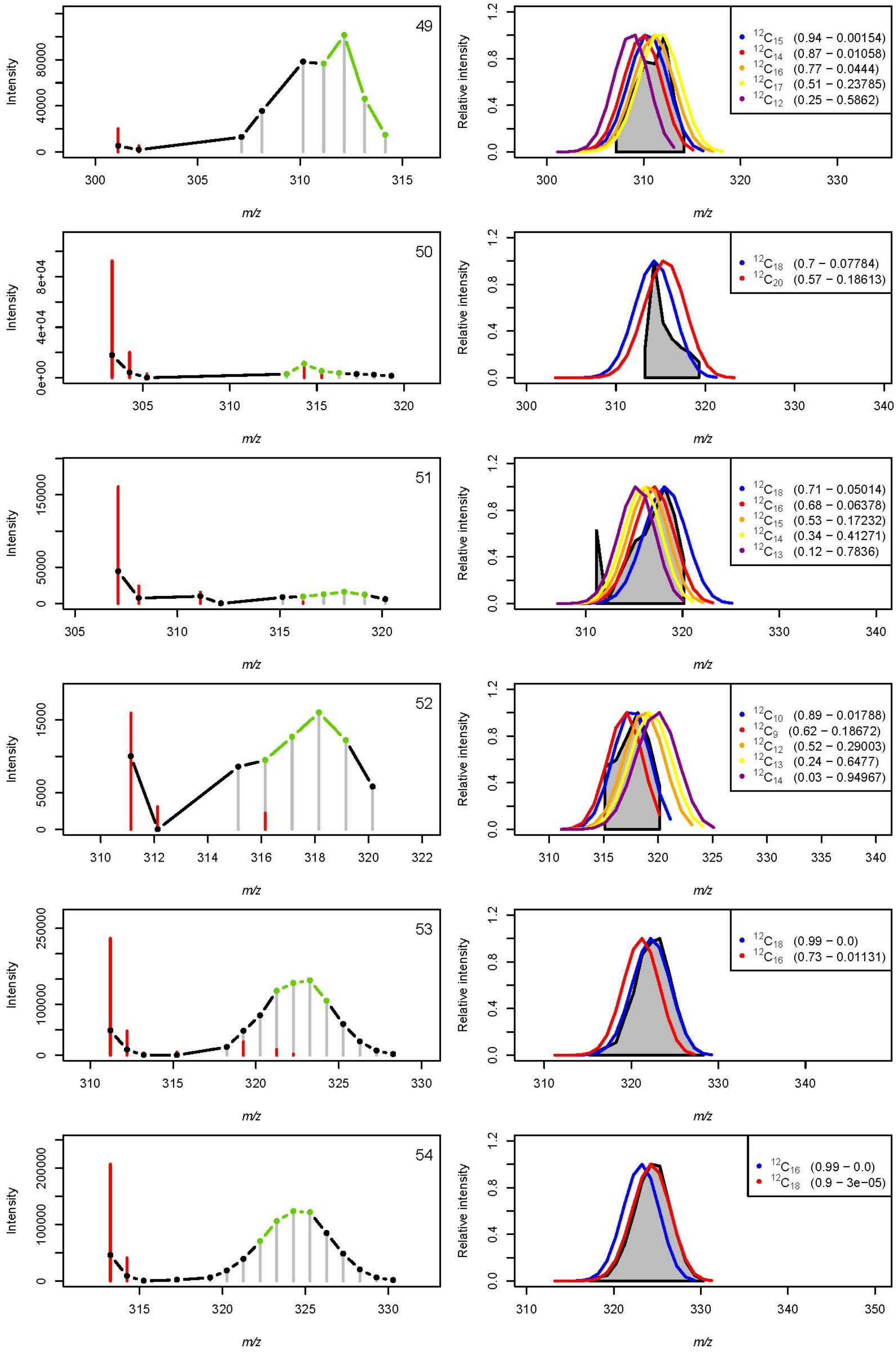

Figure S5. *Cont.*

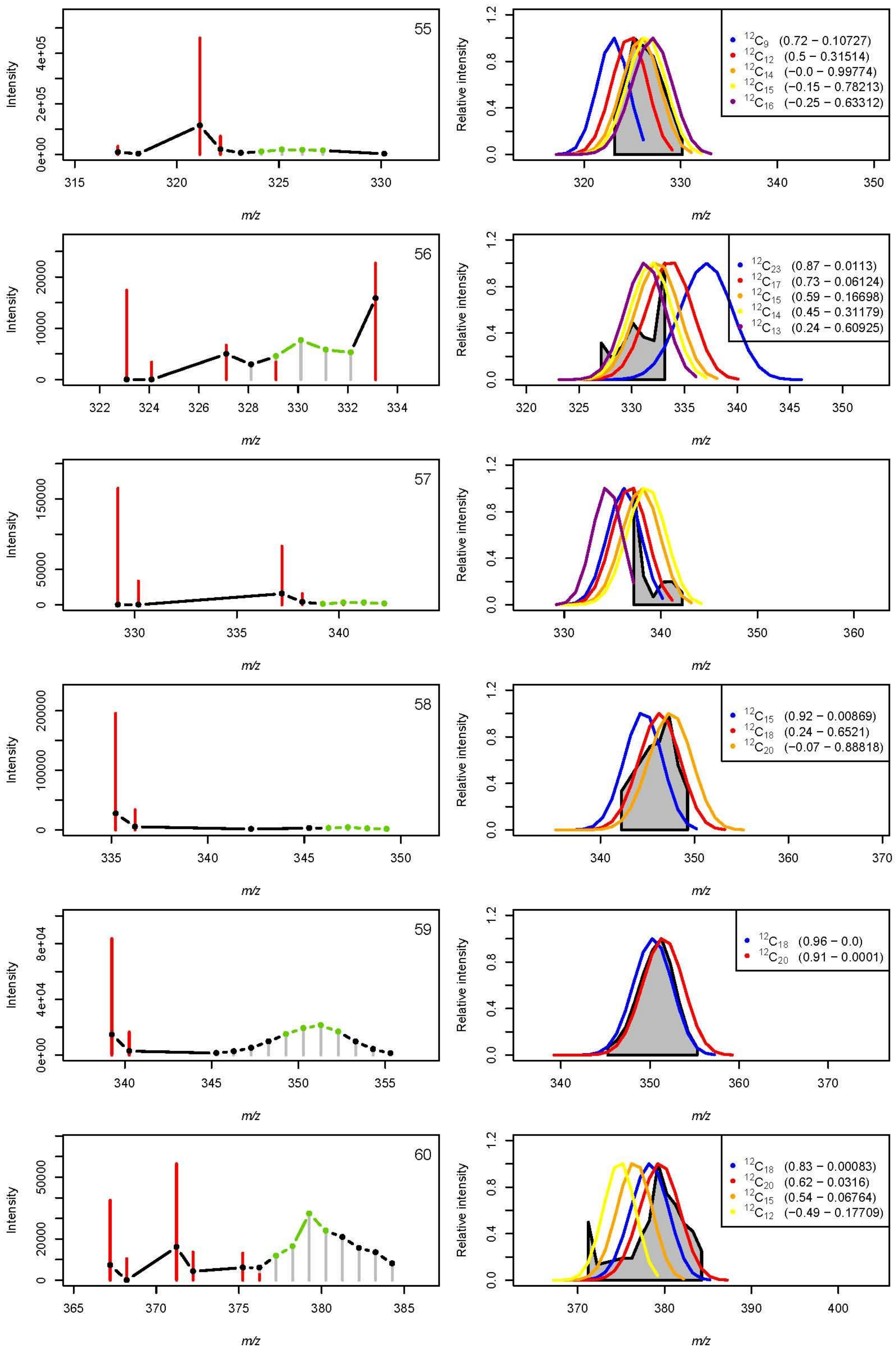

Figure S5. *Cont.*

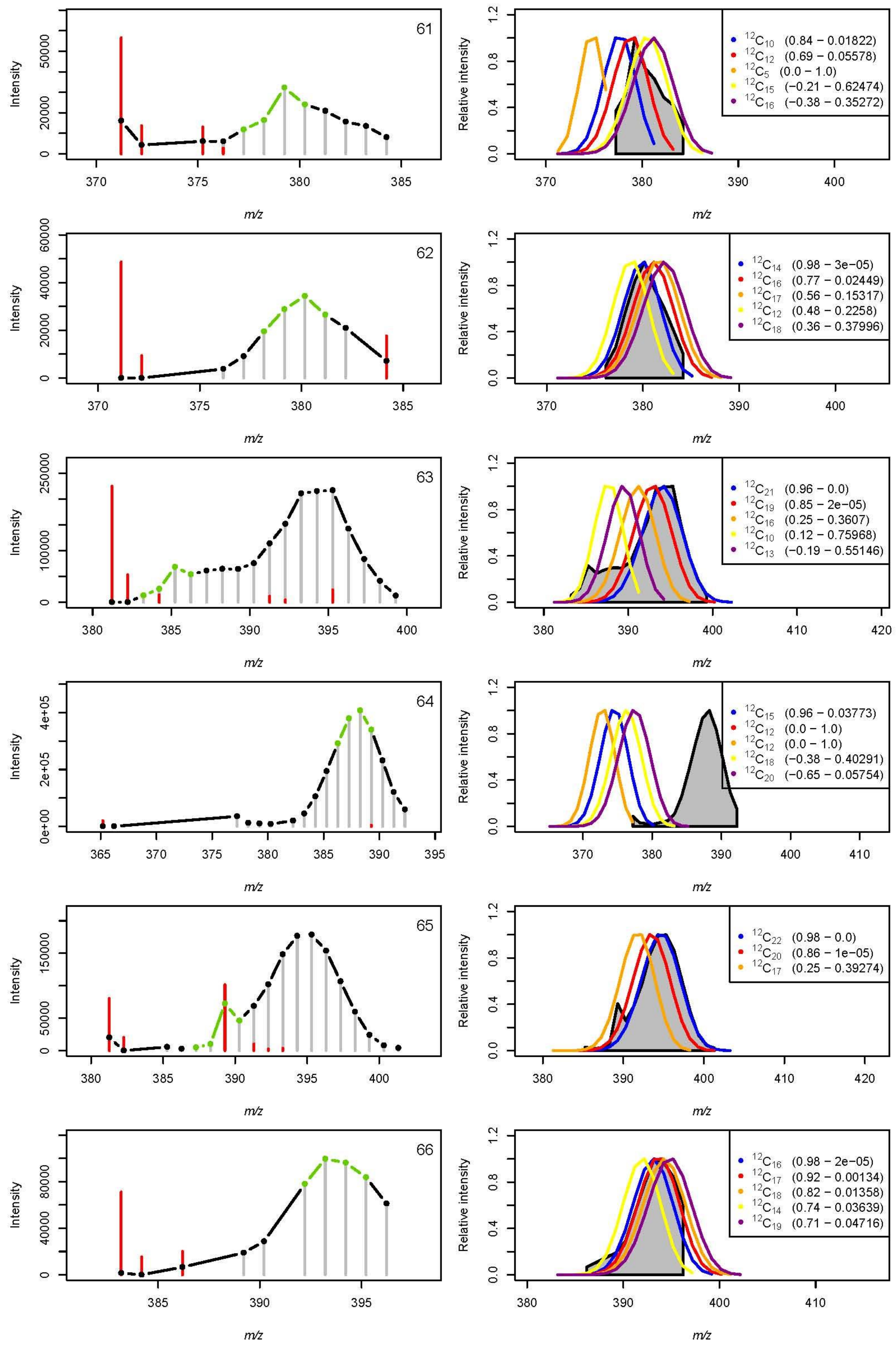

Figure S5. *Cont.*

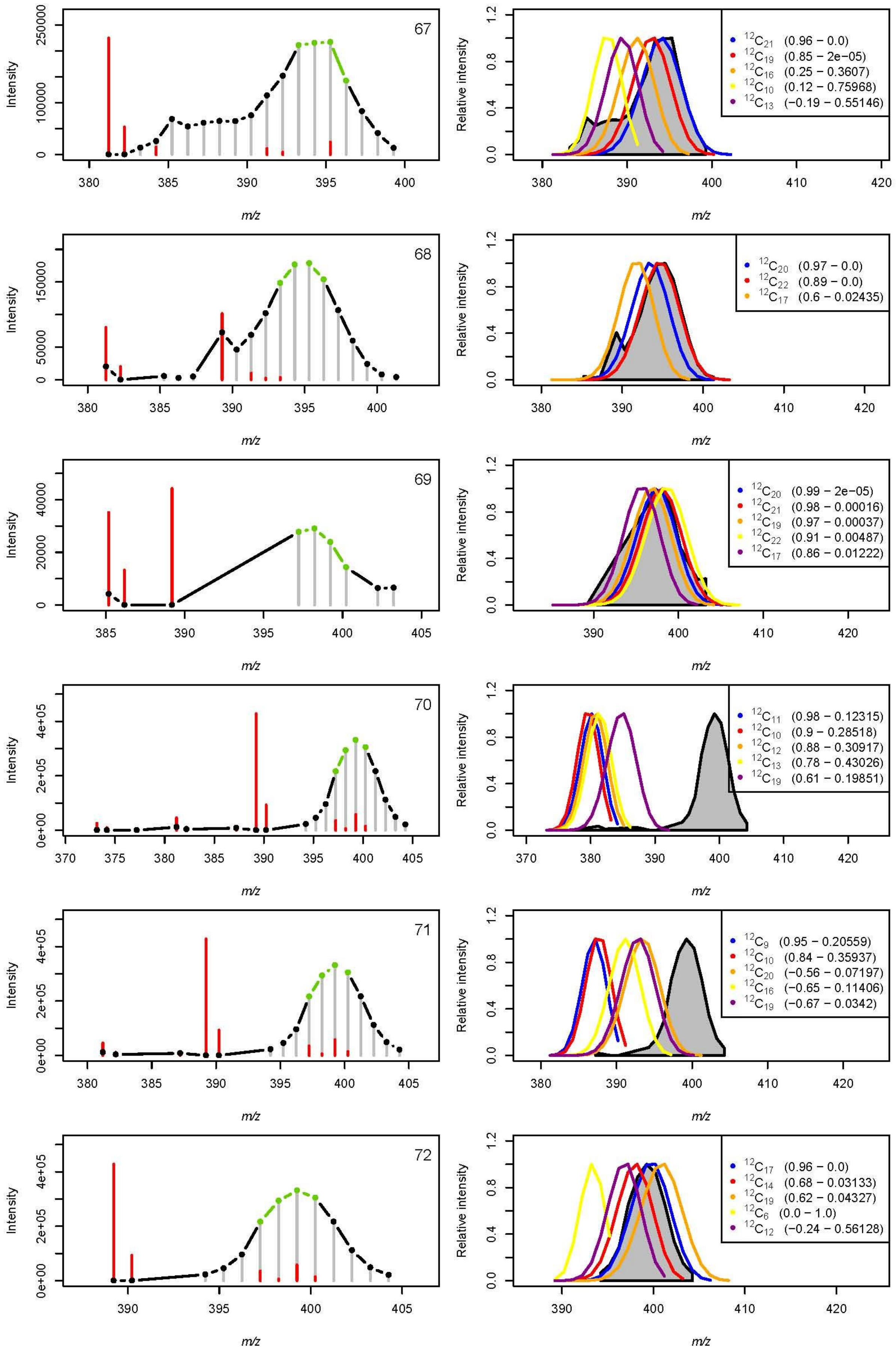

Figure S5. *Cont.*

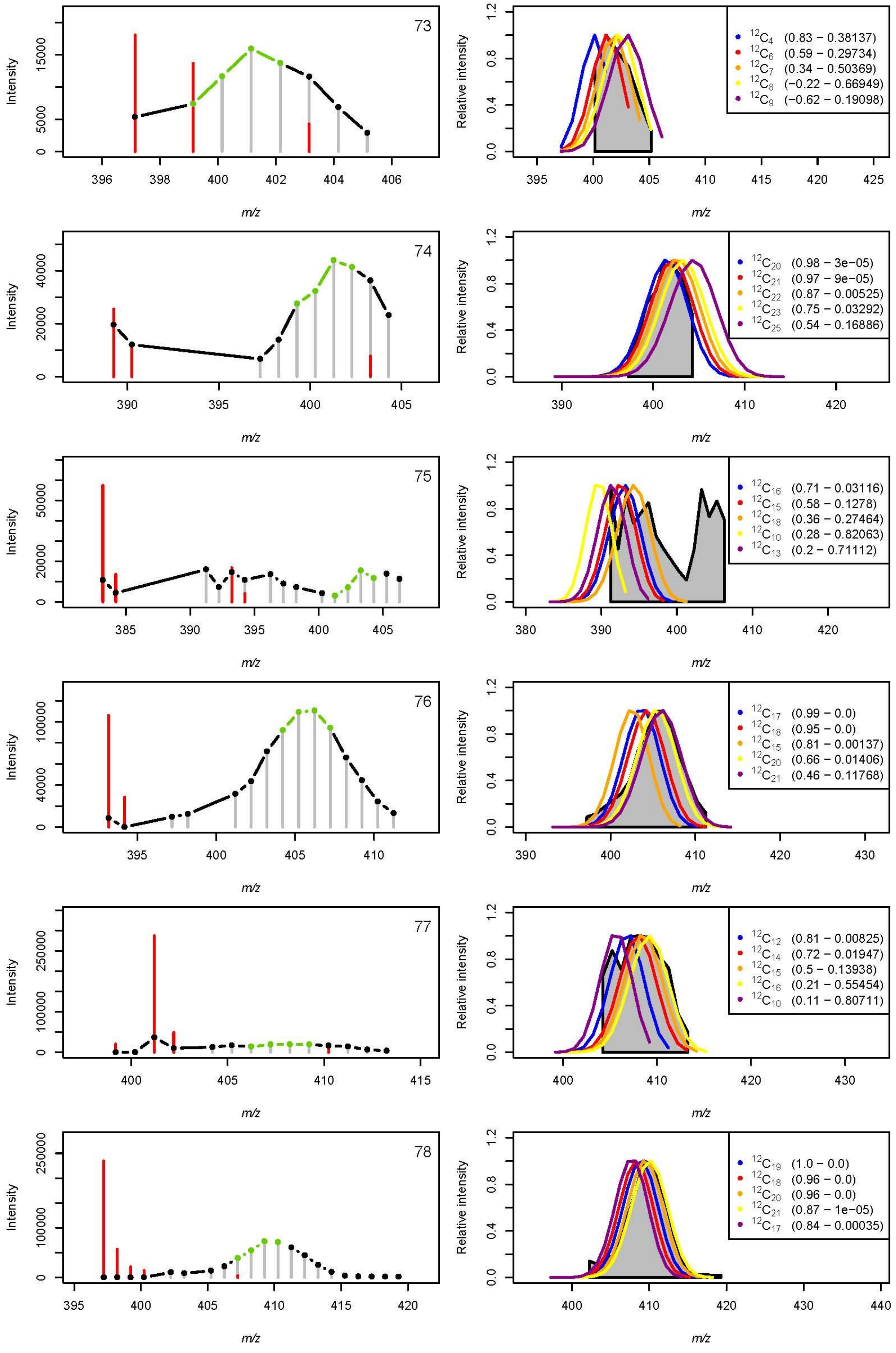

Figure S5. *Cont.*

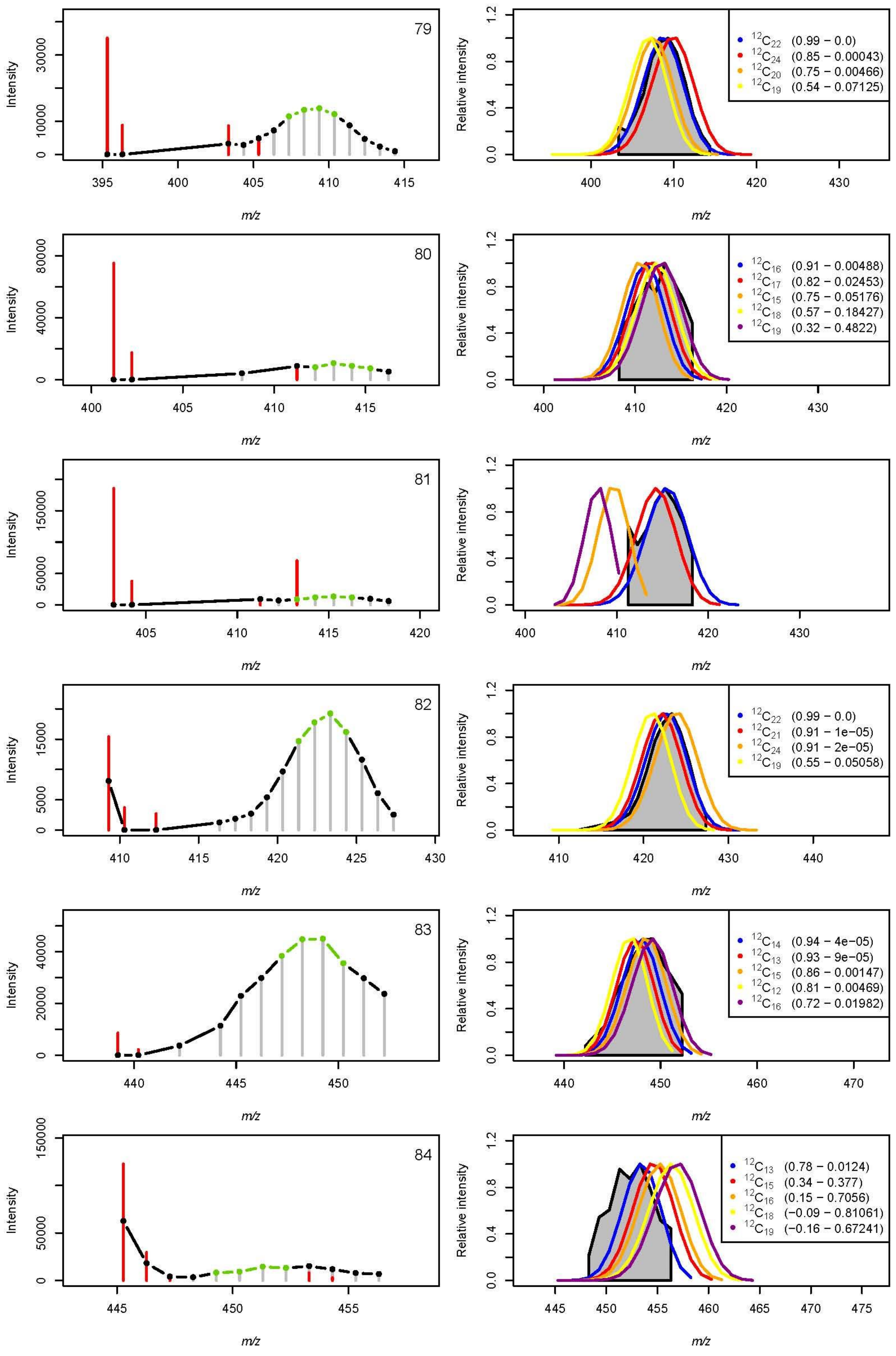

Figure S5. *Cont.*

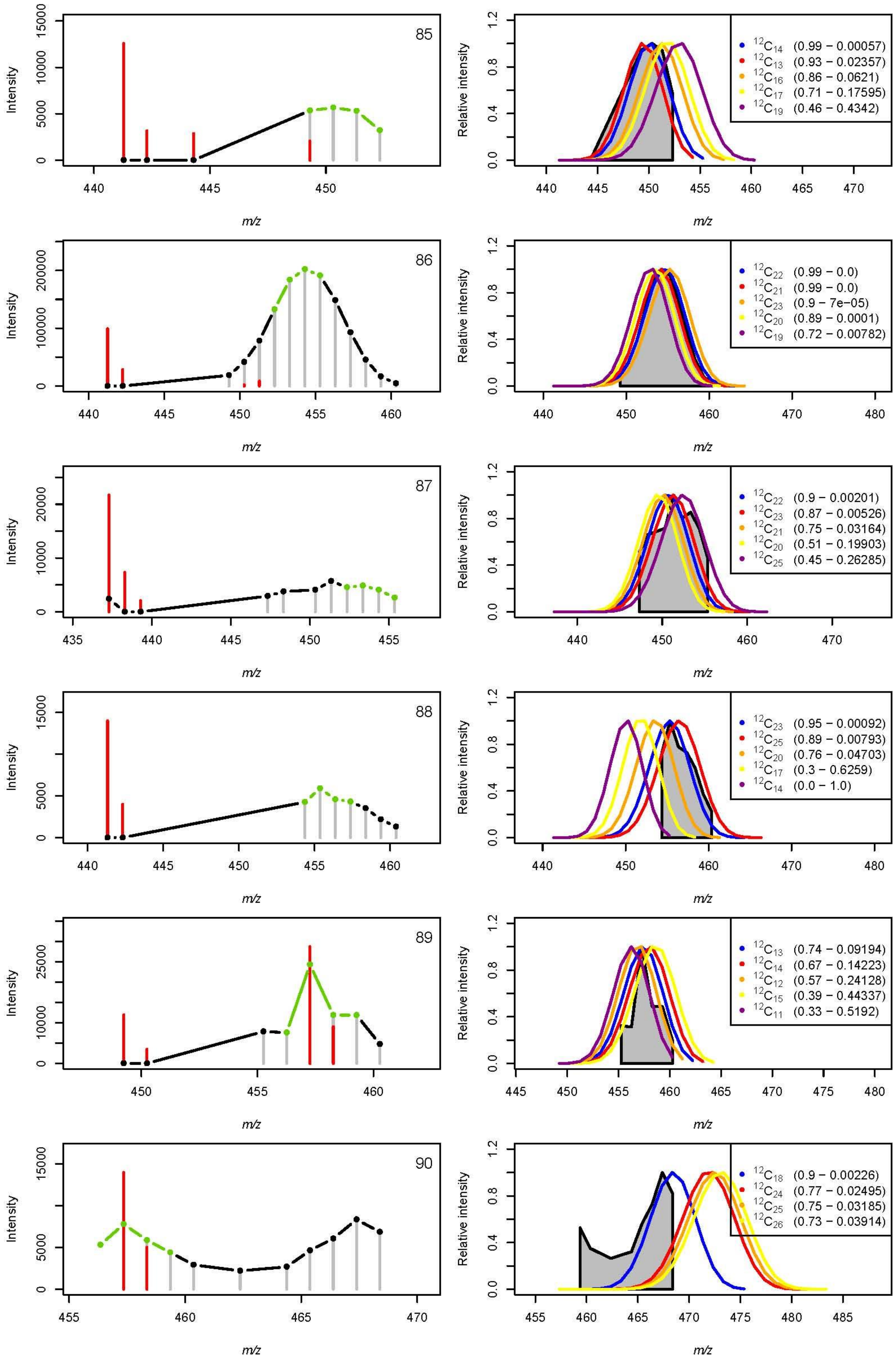

Figure S5. *Cont.*

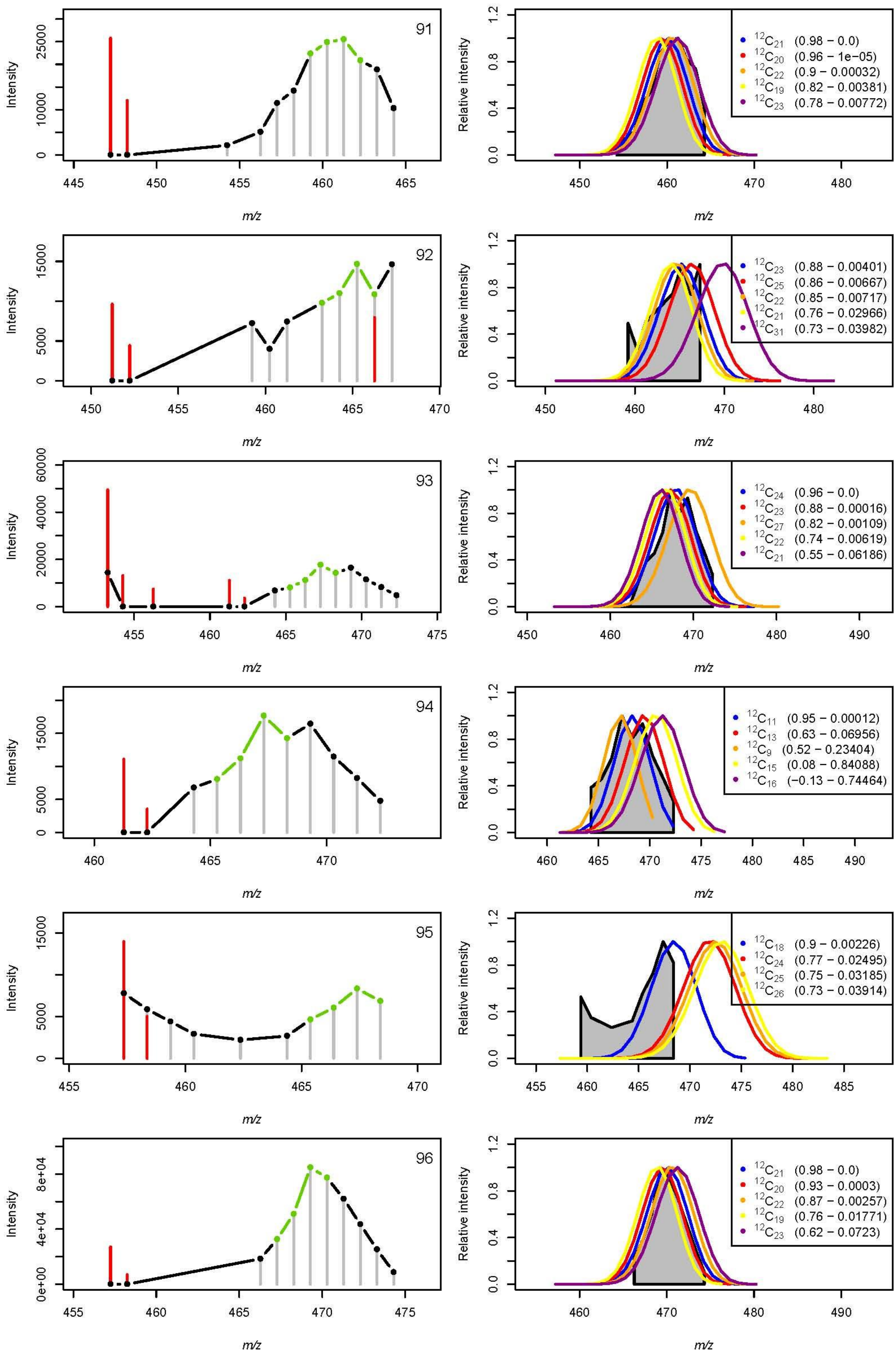

Figure S5. *Cont.*

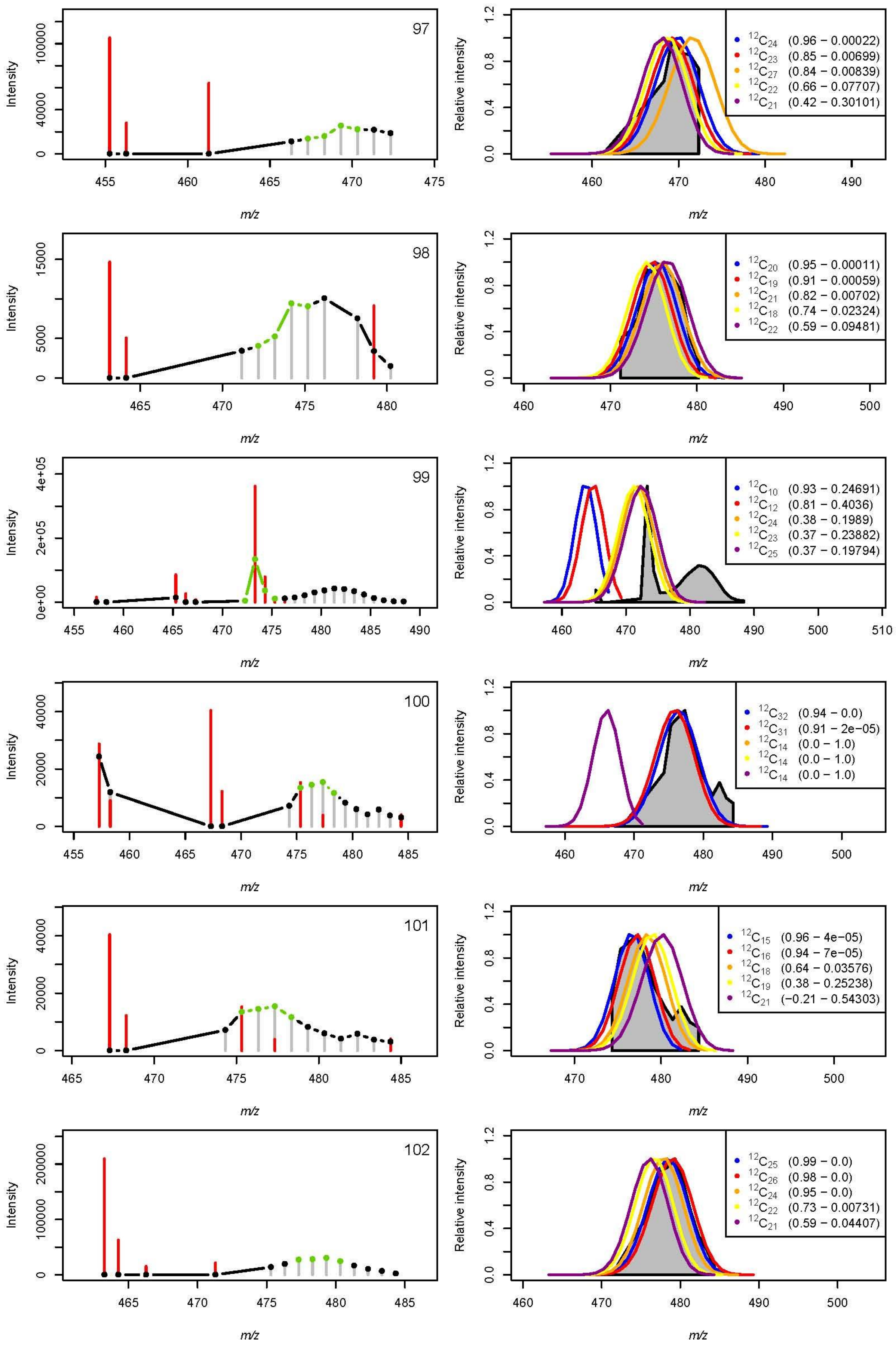

Figure S5. *Cont.*

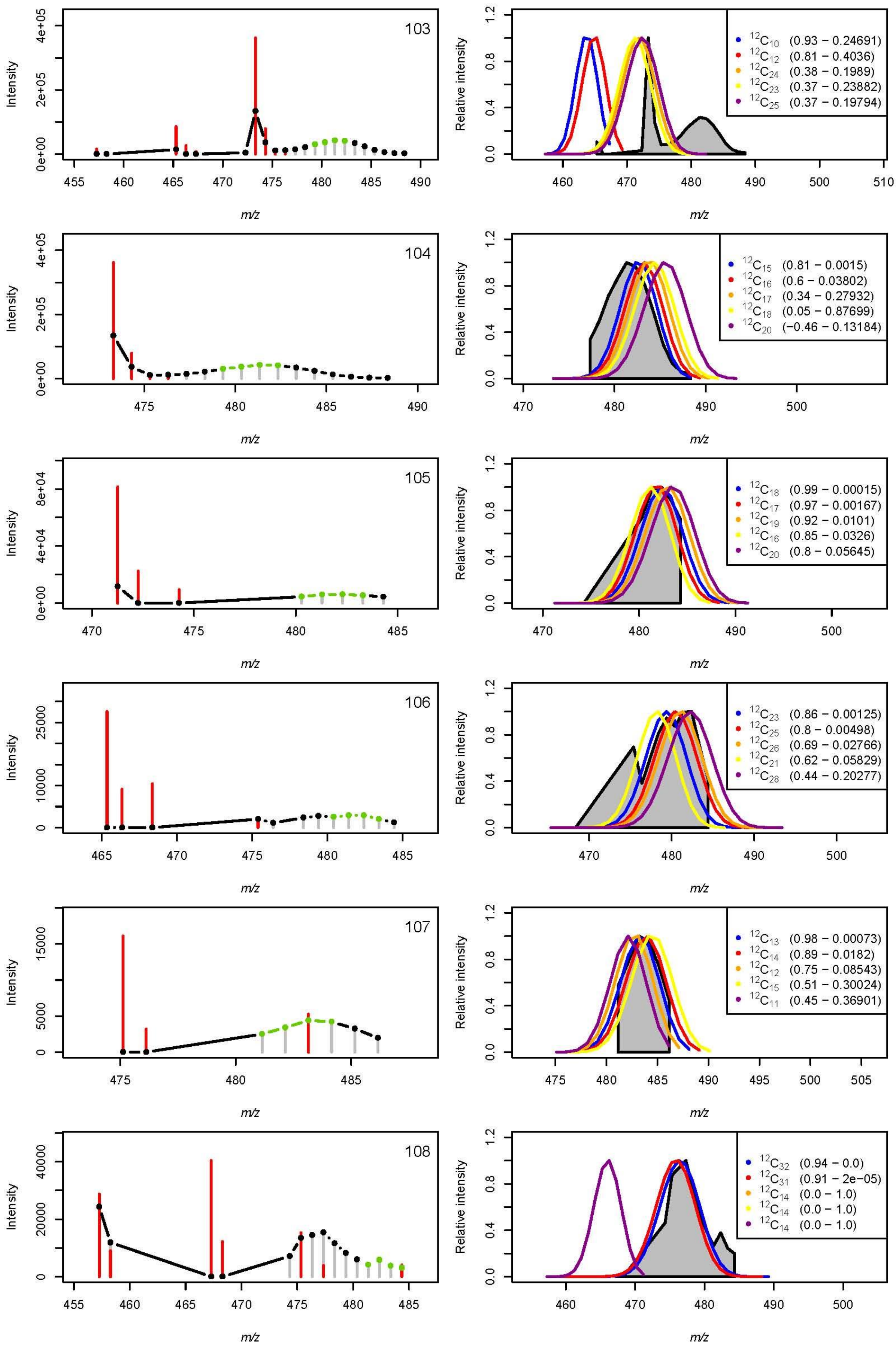

Figure S5. *Cont.*

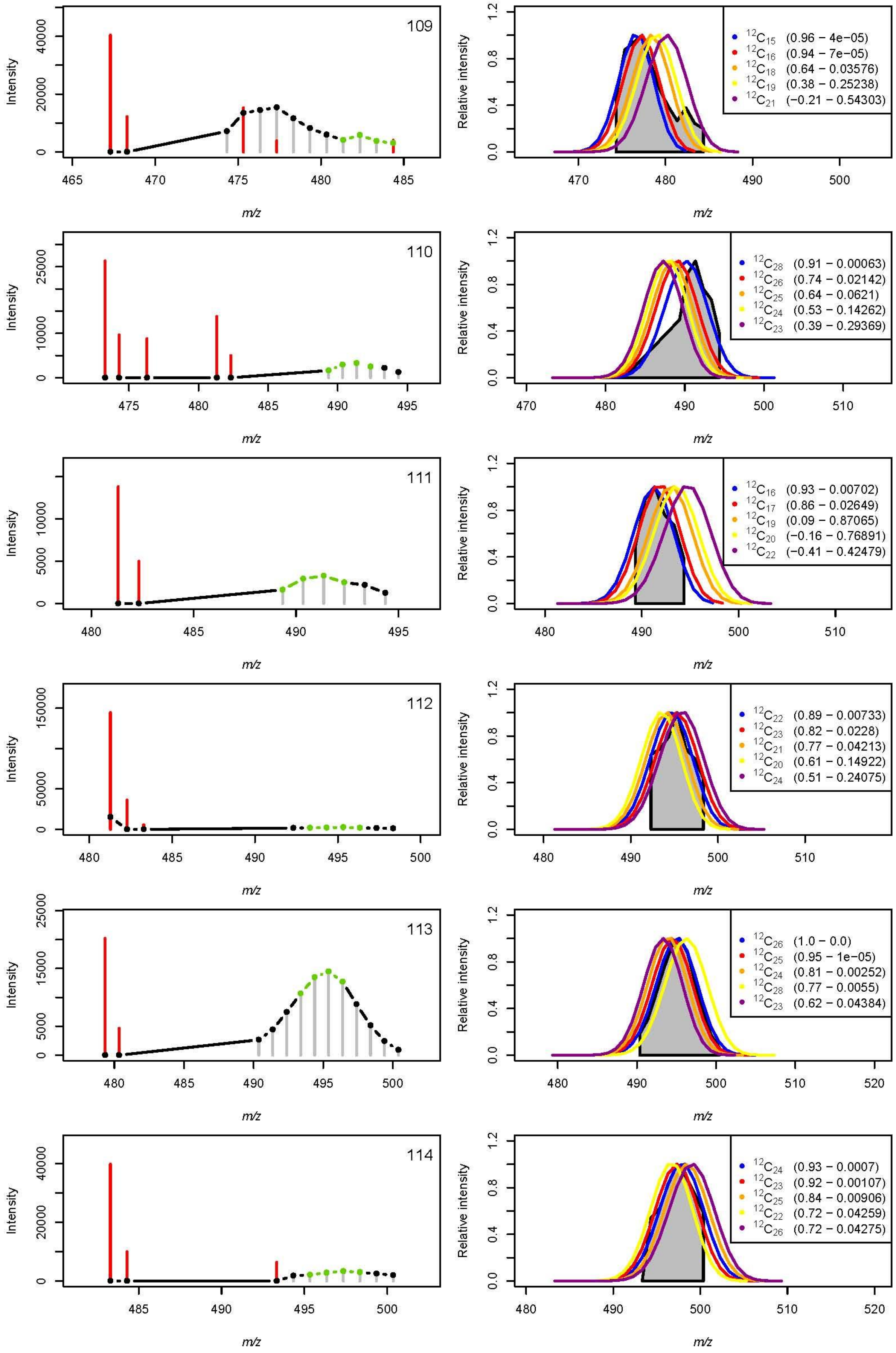

Figure S5. *Cont.*

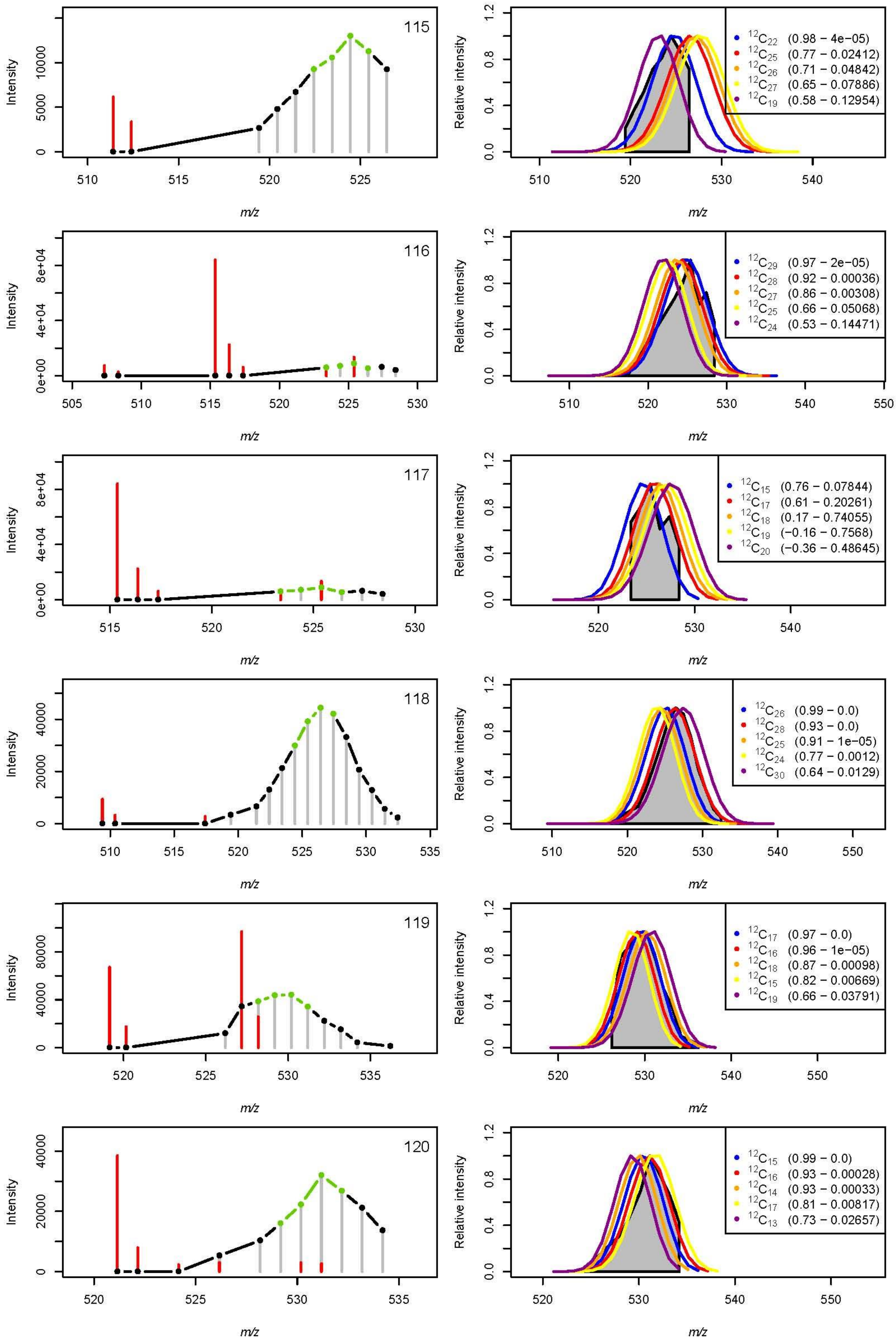

Figure S5. *Cont.*

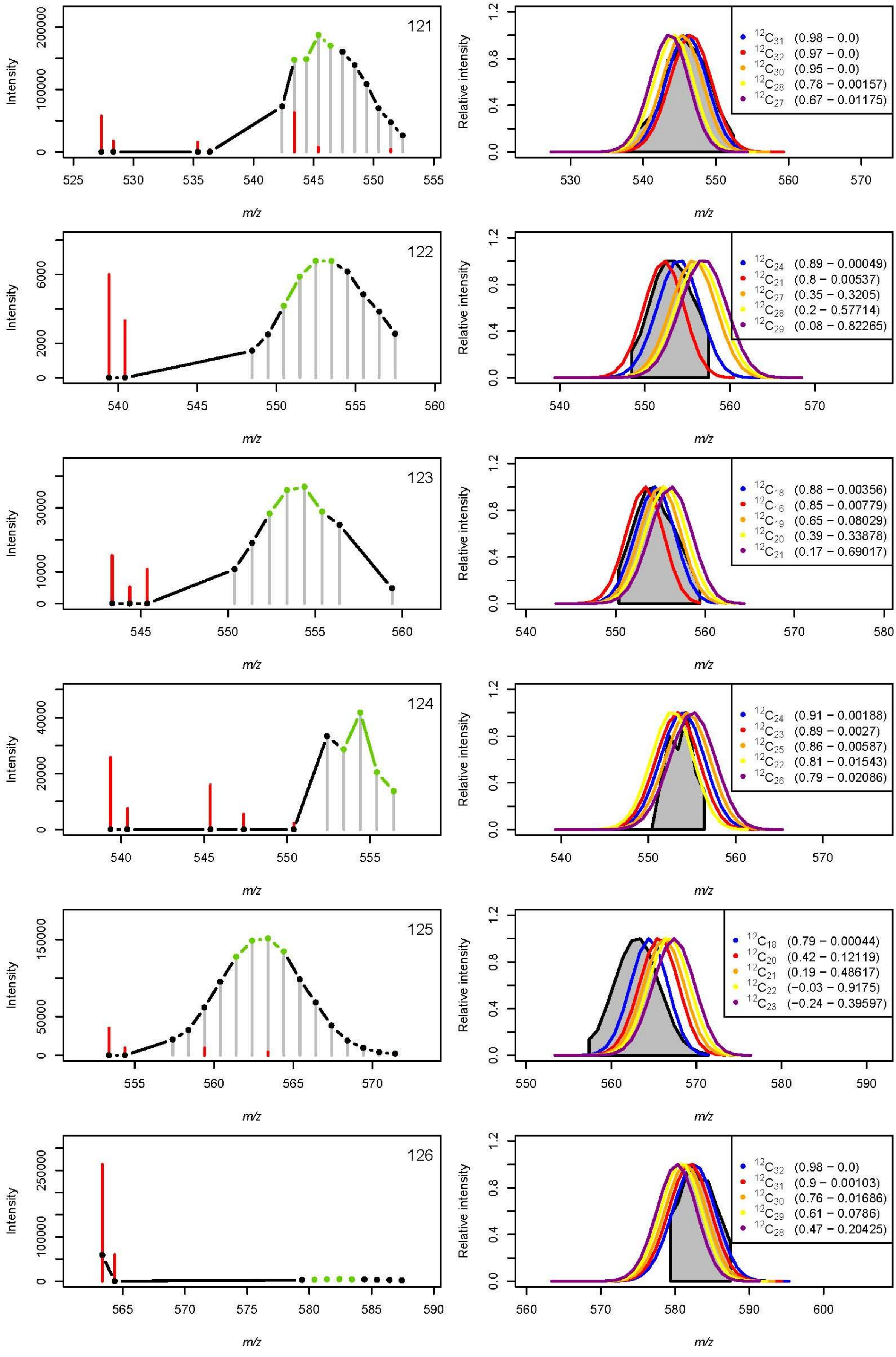

Supplement: Supplementary File 1 — Supplementary Materials (PDF, 8522 KB) [file marinedrugs-11-04158-s001.pdf]
